# Effectiveness of person‐ and family‐centred care transition interventions on patient‐ oriented outcomes: A systematic review

**DOI:** 10.1002/nop2.677

**Published:** 2020-11-19

**Authors:** Chantal Backman, Julie Chartrand, Michelle Crick, Robin Devey Burry, Orvie Dingwall, Beverley Shea

**Affiliations:** ^1^ School of Nursing Faculty of Health Sciences University of Ottawa Ottawa Canada; ^2^ Clinical Epidemiology Program Ottawa Hospital Research Institute Ottawa Canada; ^3^ Bruyère Research Institute Ottawa Canada; ^4^ Neil John Maclean Health Sciences Library University of Manitoba Winnipeg Manitoba Canada

**Keywords:** care transitions, nurses, nursing, patient outcomes, person‐ and family‐centred care, systematic review

## Abstract

**Aim:**

The aim was to critically analyse the body of evidence regarding the effectiveness of PFCC transition interventions on the quality of care and the experience of patients.

**Design:**

We conducted a systematic review using the Cochrane Handbook's guidelines and adhered to a standardized reporting format: Preferred Reporting Items for Systematic Reviews and Meta‐Analyses (PRISMA).

**Methods:**

Four databases and grey literature were searched. Following a two‐step screening process, data from the eligible studies were extracted. Risk of bias and quality of the studies were also assessed. Narrative synthesis and vote counting were used for the data analysis.

**Results:**

A total of 28 articles met our inclusion criteria. Interventions varied in regards to the extent of the PFCC focus and the comprehensiveness of the transition of care. Educating patients to promote self‐management was the most commonly included component and it was described in all 28 interventions.

## 1 INTRODUCTION

Care transitions from hospital to home can pose significant risks to patients, increasing the probability of adverse events and suboptimal patient outcomes, as well as increasing the risk of emergency department visits or hospital readmissions (Anthony et al., 2005; Greenwald et al., 2007). Not only can flawed transitions in care result in negative patient outcomes, they also can result in decreased patient experience and increased economic pressure on the healthcare system (Forster et al., 2003; Kripalani et al., 2007; Laugaland et al., 2012; McMurray et al., 2013).

## 2 BACKGROUND

Person‐ and family‐centred care (PFCC) is defined as care that is “grounded in mutually beneficial partnerships among health care providers, patients and families” (Institute for Patient‐ and Family‐Centered Care^2019^, n.d.). Sidani et al. (2014) have conceptualized PFCC as comprising elements of holistic care, collaborative care and responsive care. Patient and family engagement is integral to a PFCC approach and it has been identified as a key domain in an ideal transition from the hospital to the community environment (Burke et al., 2013) and as essential to improving overall patient care in our healthcare system (Carman et al., 2013; Manafo et al., 2018). This is supported by prior research that has found that patients who are more involved in the decision‐making process related to their care have reduced anxiety and stress (Anderson et al., 1995; Veilleux et al., 2018, are better able to manage complex chronic conditions (Coleman et al., 2009; Epstein et al., 2010; Osborn & Squires, 2011) and have lower healthcare use (Charmel & Frampton, 2008; Hughes et al., 2018).

Although many PFCC transition interventions have been trialed (Anthony et al., 2005; Greenwald et al., 2007; Laugaland et al., 2012), care transitions from hospital to home continue to be fragmented and pose high safety risks (Backman et al., 2018; Forster et al., 2003; Werner et al., 2018). There is a need for additional research to review the evidence on PFCC transition interventions. Although there have been several reviews examining care transitions from hospital to home (Black & Duval, 2019; Dusek et al., 2015; Allen et al., 2014; Hansen et al., 2011, only one, to our knowledge, has focused specifically on the effectiveness of PFCC transition processes (Desai et al., 2015). In this study, Desai et al. (2015) reviewed *N* = 16 trials of adult and paediatric hospital to home transition interventions. The authors observed an association between tailored discharge education and patient outcomes in four paediatric emergency department to home studies. The authors also reported favourable patient outcomes for multifaceted interventions in the adult population that included a transition needs assessment (*N* = 4) or an individualized transition record (*N* = 6). However, this paper limited its search to studies conducted in one country, the United States, potentially limiting the number of studies in the review and their ability to draw conclusions about the effectiveness of PFCC focused interventions used worldwide. In the present review, we expanded the search to include other countries and we broadened the search terms. Our research question was: What is the effect of PPFC interventions on the quality of care and the experience of patients during the transition from hospital to home?

## 3 METHODS

### 3.1 Research design and methodology

We conducted a systematic review (SR) of studies reporting the results of PFCC transition interventions on the quality of care and the experience of patients during care transitions between the hospital and home. This review was conducted as part of a larger research project from a published protocol (Backman et al., 2017). We prepared this report in accordance with the PRISMA (Preferred Reporting Items for Systematic Reviews and Meta‐Analyses) guidelines (Moher et al., 2009), available in Supplementary Material: Table S1. This SR is registered with PROSPERO. Ethical approval was not applicable.

### 3.2 Changes to protocol

In this paper, due to the large amount of data, we focused specifically on the patient‐oriented outcomes (e.g. condition‐specific knowledge, self‐care behaviours, functional status, adverse events, quality of life, medication adherence, adherence to follow‐up and satisfaction). The health system‐specific outcomes (e.g. 30‐day emergency department visits and 30‐day readmissions) will be reported on in a subsequent paper.

### 3.3 Study selection and eligibility criteria

Eligible articles were those that met the inclusion and exclusion criteria as follows:

#### 3.3.1 Population

The population of interest was the adult population (18 years of age or older).

#### 3.3.2 Interventions

Interventions of interest were any PFCC transition interventions (e.g. individualized discharge plan, individualized transition record, postdischarge telephone follow‐up, home visits, person‐ and family‐tailored discharge information and transition need assessment) that included at least one element from each of the components (i.e. holistic care, collaborative care and responsive care) of the PFCC framework (Sidani et al., 2014).

#### 3.3.3 Context

The context was care transitions between the hospital and home.

#### 3.3.4 Outcomes (patient‐oriented)

The outcomes were knowledge (condition‐specific), self‐care behaviours, functional status, adverse events, quality of life, medication adherence, adherence to follow‐up and satisfaction.

#### 3.3.5 Study designs

Randomized controlled trials were included. Studies that were non‐randomized experimental studies (e.g. cohort, case–control, controlled before and after, interrupted time series and controlled trials not using full randomization), qualitative, editorials, commentaries or study protocols were excluded from this review. PFCC transition interventions from emergency departments to home were also excluded. Studies related to obstetrics and gynaecology care and psychiatric or mental health services were also excluded.

### 3.4 Search strategy

Eligible articles were identified through a systematic search of the MEDLINE, CINAHL, Cochrane Central Register of Controlled Trials and the Cochrane Consumers and Communication Group databases, from inception to 25 November 2016. The search used terms on PFCC and care transitions. The search strategy was developed by an experienced health sciences information specialist, was externally peer reviewed by an experienced librarian using the Peer Review of Electronic Search Strategies checklist (McGowan et al., 2016), then was updated to reflect the included studies. The MEDLINE search strategy is provided in Table S2. Key journals and grey literature websites were also searched. Review data were managed using Covidence systematic review software for screening, full‐text review, data extraction and risk of bias (Covidence systematic review software^2019^).

### 3.5 Screening

Records were independently screened by two reviewers with conflicts resolved by consensus. Marginally relevant records and records with insufficient information to determine eligibility were retained. Full texts of retained studies were independently assessed by two reviewers with discrepancies resolved by consensus. Screening criteria were piloted and modified as required.

### 3.6 Data extraction

Two reviewers independently extracted data into a standardized form, that had previously been piloted. Data extracted included the full reference, objectives, target population, description of the intervention and control, PFCC elements, outcome measures, design, length of postintervention follow‐up period and study results.

The PFCC elements (Sidani et al., 2014) reported in each intervention were described. Trials required a minimum of one element from each of the holistic care, collaborative care and responsive care components for inclusion in the review; the full list was included to provide an indication of the extent of the PFCC focus of the interventions. Similarly, interventions were matched by the components of an ideal transition as described by Burke et al. (2013). This was done to provide an indication of the comprehensiveness of the intervention for care transitions.

### 3.7 Risk of bias assessment

Study quality was assessed using the Cochrane Risk of Bias tool (Higgins et al., 2011). Two reviewers independently assessed the risk of bias of each included study with respect to the following seven domains: sequence generation, allocation concealment, blinding of participants and personnel, blinding of outcome assessment, missing outcome data, selective outcome reporting and other sources of bias. Disagreements were resolved by discussion and by consulting a third reviewer when necessary. Studies were evaluated as “low” (unlikely plausible risk of bias that could alter confidence in the results), “unclear” (plausible bias that raised a doubt of the validity of the results) or “high” (plausible bias that seriously weakened the confidence in the results) as per the criteria.

### 3.8 Data analysis

A meta‐analysis was not conducted due to the heterogeneity of the results, the diversity of outcomes reported and the variety of testing conditions under which accuracy measures were examined and reported among the studies included in the review. Therefore, we conducted a narrative synthesis and used a vote‐counting approach for data analysis (Grimshaw et al., 2003). For vote counting, we counted the number of studies that resulted in statistically significant effects as well as the number of studies that showed non‐significant effects for each outcome of interest (Grimshaw et al., 2003). To determine if a PFCC transition intervention was related to a specific outcome, we required a significant relationship to be present in a minimum of three articles between the PFCC transition intervention and the individual outcome (Godin et al., 2008). To supplement this approach, tables were developed to illustrate the magnitude of the effect for statistically significant results (Grimshaw et al., 2003).

## 4 RESULTS

### 4.1 Study selection

We screened 6,127 unique titles and abstracts for potential eligibility after removing duplicates. Following screening, we excluded 5,571 articles and reviewed the remaining 556 full text. A total of 28 trials met the final inclusion criteria. The screening and selection process using a PRISMA flow chart is illustrated in Fig S1. Excluded studies (*N* = 528) and reasons for exclusion are provided in the Table S3. Studies were excluded most often because they had the wrong outcome or intervention, or the wrong population.

### 4.2 Description of studies

Table 1 provides details of each trial included in this review. The included studies were published between 1996 and 2017 in a variety of countries: United States (*N* = 14), Australia (*N* = 3), Netherlands (*N* = 2), China (*N* = 2), Canada (*N* = 1), Philippines (*N* = 1), Taiwan (*N* = 1), Slovenia (*N* = 1), Italy (*N* = 1), Brazil (*N* = 1) and Norway (*N* = 1).

**Table 1:** Study characteristics (*N* = 28)

Study/year/location	Population	Description of Intervention	PFCC elements (Sidani et al., 2014)	Components of Ideal transition care (Burke et al., 2013)	Outcomes
Bostrom (1996) United States	Adults	**Telephone Nursing Care Link Project** Nurse‐initiated telephone follow‐up 2–3 days postdischarge and as required Patient‐initiated telephone follow‐up using educational brochure as a guide.	**5‐LOW** **Holistic (2)** Identify patient's concerns and/or needs Monitor or reassess patient's needs **Collaborative (1)** Answer questions patient may have about his/her care **Responsive (2)** Identify changes in patient's condition or feeling and act upon them Make sure patient has what he/she needs with regards to his/her health care	**6‐LOW** Discharge planning Availability, timeliness, clarity and organization of information Educating patients, promoting self‐management Coordinating care among team member Monitoring and managing symptoms after discharge Follow‐up with outpatient providers	Satisfaction with education
Kangovi, S (2014) United States	Adults (18–64 years)	**IMPaCT‐ Patient‐Centered Community Health Worker Intervention** In‐hospital goal setting with Community Health Worker (CHW) with high‐risk, low‐SES patients CHW liaison with healthcare team regarding goals set by pt. Tailored postdischarge telephone support, encouragement and care coordination Standardized protocols implemented for CHW role	**10‐MOD** **Holistic (1)** Assess patient's health values and goals **Collaborative (6)** Support patient decision‐making—share information in a complete and unbiased way regarding condition, prognosis, treatment Promote discussion with patient to find a common understanding of what the problem is Assess patient's preferences for treatment or self‐management Provide the chosen treatment option or self‐management strategy Provide instructions to patient on how to apply treatment option or self‐management strategy in daily life Provide support, as needed, to patient for the application of treatment option or self‐management strategy in daily life **Responsive (3)** Make sure patient has what he/she needs with regards to his/her health care Make sure patient has what he/she needs with regards to community resources Comfort the patient when needed	**8‐LOW** Discharge planning Complete communication of information Availability, timeliness, clarity and organization of information Educating patients, promoting self‐management Enlisting help of social and community supports Coordinating care among team member Monitoring and managing symptoms after discharge Follow‐up with outpatient providers	Patient activation Medication adherence Mental health Physical health Discharge communication Satisfaction
Naylor, M. D. (1999) United States	Adults	**Comprehensive discharge planning and home follow‐up** APN visits with pts and caregivers every 48hrs from admission to discharge At least 2 at home visits (first within 48hrs postdischarge) by APN Weekly follow‐up calls by APN	**12‐MOD** **Holistic (3)** Assess patient's health values and goals Identify patient's concerns and/or needs Monitor or reassess patient's needs **Collaborative (6)** Promote discussion with patient to find a common understanding of what the problem is Explain to patient the treatment options and self‐management strategies available to manage the problem Answer questions patient may have about his/her care Assess patient's preferences for treatment or self‐management Provide the chosen treatment option or self‐management strategy Incorporate the patient and family in patient care **Responsive (3)** Modify the type, mode of delivery or dose of treatment or self‐management strategy to be consistent with patient's needs and preferences Make sure patient has what he/she needs with regards to his/her health care Make sure patient has what he/she needs with regards to community resources	**9‐MOD** Discharge planning Complete communication of information Availability, timeliness, clarity and organization of information Medication safety Educating patients, promoting self‐management Enlisting help of social and community supports Coordinating care among team member Monitoring and managing symptoms after discharge Follow‐up with outpatient providers	Functional status Depression Satisfaction
Altfeld, S.J. (2013) United States	Older Adults (≥65 years)	**Enhanced Discharge Planning Program** Telephone follow‐up by social worker postdischarge (within 2 working days) Follow‐up by social worker with service providers if additional services required	**9‐LOW** **Holistic (4)** Comprehensively assess patients’ condition including physical, emotional, social and spiritual domains of health Identify patient's concerns and/or needs Monitor or reassess patient's needs Provide interventions/services to patient that address all domains of health including physical comfort and emotional support **Collaborative (1)** Answer questions patient may have about his/her care **Responsive (4)** Respond to patient's needs, beliefs, values and preferences Take time to answer patient questions Make sure patient has what he/she needs with regards to his/her health care Make sure patient has what he/she needs with regards to community resources	**7‐LOW** Discharge planning Availability, timeliness, clarity and organization of information Educating patients, promoting self‐management Enlisting help of social and community supports Coordinating care among team member Monitoring and managing symptoms after discharge Follow‐up with outpatient providers	Patient stress Caregiver stress Patient assessment of health Physician communication
Chan (2015) United States	Older Adults, (≥55)	**Nurse‐led Hospital‐based Care Transition Intervention** 2 nurse visits in hospital (2nd within 24hrs of discharge) 2 postdischarge phone calls by NP Participant access to NP telephone support within 24hrs of call	**6‐LOW** **Holistic (1)** Provide information on disease and self‐management to patient **Collaborative (3)** Promote discussion with patient to find a common understanding of what the problem is Answer questions patient may have about his/her care Incorporate the patient and family in patient care **Responsive (2)** Identify changes in patient's condition or feeling and act upon them Make sure patient has what he/she needs with regards to community resources	**9‐MOD** Discharge planning Complete communication of information Availability, timeliness, clarity and organization of information Medication safety Educating patients, promoting self‐management Enlisting help of social and community supports Coordinating care among team member Monitoring and managing symptoms after discharge Follow‐up with outpatient providers	Transitional care experience Discharge communication
Courtney (2009) Australia	Older Adults (≥65)	**Multifaceted transitional care intervention** Individually tailored nurse‐led 24‐week intervention Home visit and regular telephone follow‐up calls (weekly first 4 wks, monthly for further 5 mths)	**6‐LOW** **Holistic (4)** Assess patient's health values and goals Identify patient's concerns and/or needs Monitor or reassess patient's needs Provide information regarding health promotion, illness prevention or lifestyle change to patient **Collaborative (1)** Answer questions patient may have about his/her care **Responsive (1)** Make sure patient has what he/she needs with regards to his/her health care	**4‐LOW** Discharge planning Availability, timeliness, clarity and organization of information Educating patients, promoting self‐management Monitoring and managing symptoms after discharge	Quality of life
Li, H. (2012) United States	Adults (≥21 years) who were primary caregivers for a hospitalized older adult (≥65 years)	**An intervention program (CARE: Creating Avenues for Relative Empowerment)** Nurse‐led two‐session empowerment‐educational program within 1–2 days of admission Assistance with development of care plan based on their abilities and preferences Education material provided on variety of topics e.g. management of behaviour, hospital to home transition and self‐care	**3‐LOW** **Holistic (1)** Provide information on disease and self‐management to patient **Collaborative (1)** Incorporate the patient and family in patient care **Responsive (1)** Make sure patient has what he/she needs with regards to his/her health care	**4‐LOW** Discharge planning Availability, timeliness, clarity and organization of information Educating patients, promoting self‐management Monitoring and managing symptoms after discharge	Depressive symptoms Patient's cognitive status Functional status
Cajanding (2017) Philippines	Adults, Cardiac	**Structured Discharge Planning Program** Cardiovascular nurse practitioner led three 30 to 45 min. daily sessions on problem‐solving, goal setting and action planning. A disease‐specific handbook with information on the program, aims of therapy, checklist and FAQs was provided.	**8‐LOW** **Holistic (3)** Identify patient's concerns and/or needs Provide information regarding health promotion, illness prevention or lifestyle change to patient Provide information on disease and self‐management to patient **Collaborative (3)** Explain to patient the treatment options and self‐management strategies available to manage the problem Answer questions patient may have about his/her care Assess patient's preferences for treatment or self‐management **Responsive (2)** Provide support, as needed, to patient for the application of treatment option or self‐management strategy in daily life Respond to patient's needs, beliefs, values and preferences	**3‐LOW** Discharge planning Availability, timeliness, clarity and organization of information Educating patients, promoting selfmanagement	Cardiac self‐efficacy Functional status Satisfaction
Davis,K.K. (2012) United States	Adults (≥21), Cardiac	**Targeted self‐care teaching intervention using principles of cognitive training** In‐hospital cognitive training‐based intervention delivered by case manager Tailored to assist in integration of self‐care into personal routine and environment. An educational booklet and recording of teaching session was provided to pts with self‐care supplies for discharge CM follow‐up by phone call within 72 hr of discharge	**4‐LOW** **Holistic (2)** Provide information regarding health promotion, illness prevention or lifestyle change to patient Provide information on disease and self‐management to patient **Collaborative (1)** Answer questions patient may have about his/her care **Responsive (1)** Make sure patient has what he/she needs with regards to his/her health care	**5‐LOW** Discharge planning Availability, timeliness, clarity and organization of information Medication safety Educating patients, promoting self‐management Monitoring and managing symptoms after discharge	Heart failure Knowledge Self‐care Depression
Hanssen, T. A (2007) Norway	Adults Cardiac	**Telephone follow‐up intervention** Nurse‐led regular telephone follow‐up during 6 mths postdischarge Individual approach to personal needs, support with coping, changes in lifestyle, risk‐factor management	**9‐LOW** **Holistic (3)** Assess patient's health values and goals Identify patient's concerns and/or needs Provide information on disease and self‐management to patient **Collaborative (4)** Promote discussion with patient to find a common understanding of what the problem is Answer questions patient may have about his/her care Provide instructions to patient on how to apply treatment option or self‐management strategy in daily life Provide support, as needed, to patient for the application of treatment option or self‐management strategy in daily life **Responsive (2)** Identify changes in patient's condition or feeling and act upon them Comfort the patient when needed	**4‐LOW** Discharge planning Availability, timeliness, clarity and organization of information Educating patients, promoting self‐management Monitoring and managing symptoms after discharge	Physical activity Smoking cessation Quality of life
Harrison, M. B. (2002) Canada	Adults Cardiac	**Transitional Care Intervention** Counselling and education including workbook and education plan Communication with home care nurse of pt. status and care needs through nursing transfer letter Follow‐up call from hospital nurse within 24hrs of discharge	**4‐LOW** **Holistic (1)** Provide information on disease and self‐management to patient **Collaborative (2)** Provide support, as needed, to patient for the application of treatment option or self‐management strategy in daily life Incorporate the patient and family in patient care **Responsive (1)** Make sure patient has what he/she needs with regards to community resources	**5‐LOW** Discharge planning Complete communication of information Availability, timeliness, clarity and organization of information Educating patients, promoting self‐management Follow‐up with outpatient providers	Quality of life
Holmes‐Rovner, M. (2008) United States	≥ 21 years Cardiac	**Outpatient telephone coaching** Six‐session weekly outpatient health behaviour change telephone‐based counselling intervention over three months. Information booklet and goal worksheets provided to pts and family	**5‐LOW** **Holistic (3)** Assess patient's health values and goals Provide information regarding health promotion, illness prevention or lifestyle change to patient Provide information on disease and self‐management to patient **Collaborative (1)** Provide support, as needed, to patient for the application of treatment option or self‐management strategy in daily life **Responsive (1)** Modify the type, mode of delivery or dose of treatment or self‐management strategy to be consistent with patient's needs and preferences	**1‐LOW** Educating patients, promoting self‐management	Physical activity Weight loss Smoking cessation Functional status Quality of life
Jaarsma, T. (2000) Netherlands	≥50 years Cardiac	**A supportive‐educative programme** Nurse‐led education and support intervention tailored to the individual to improve heart failure‐related self‐care behaviour. Telephone follow‐up within 1 week postdischarge, pts able to call with questions if needed Home visit Needs assessment provided to home care if required	**6‐LOW** **Holistic (3)** Assess patient's understanding of the presenting problem (i.e. cause, nature) Identify patient's concerns and/or needs Provide information on disease and self‐management to patient **Collaborative (2)** Answer questions patient may have about his/her care Incorporate the patient and family in patient care **Responsive (1)** Make sure patient has what he/she needs with regards to his/her health care	**5‐LOW** Discharge planning Availability, timeliness, clarity and organization of information Educating patients, promoting self‐management Enlisting help of social and community supports Monitoring and managing symptoms after discharge	Heart Failure Self‐care
Jerant et al. (2003) United States	≥40 years Cardiac	**Telenursing to reduce hospitalization for heart failure** Two telenursing interventions (video‐based or telephone‐based) trialed and compared to usual care Patients received in‐person visits at baseline and 60 days	**5‐ LOW** **Holistic (3)** Assess patient's health values and goals Identify patient's concerns and/or needs Provide information on disease and self‐management to patient **Collaborative (1)** Answer questions patient may have about his/her care **Responsive (1)** Make sure patient has what he/she needs with regards to his/her health care	**6‐LOW** Discharge planning Availability, timeliness, clarity and organization of information Medication safety Educating patients, promoting self‐management Monitoring and managing symptoms after discharge Follow‐up with outpatient providers	Quality of life Satisfaction
Laramee, A.S. (2003) United States	Adults Cardiac	**Case management intervention** 12‐week multicomponent intervention Early discharge planning and coordination of care pre‐ and postdischarge by case manager Enhanced telephone follow‐up and surveillance including 1 call within 3 days of discharge, weekly in the first month and biweekly thereafter	**10‐MOD** **Holistic (3)** Identify patient's concerns and/or needs Provide information regarding health promotion, illness prevention or lifestyle change to patient Provide information on disease and self‐management to patient **Collaborative (5)** Promote discussion with patient to find a common understanding of what the problem is Answer questions patient may have about his/her care Assess patient's preferences for treatment or self‐management Provide the chosen treatment option or self‐management strategy Provide instructions to patient on how to apply treatment option or self‐management strategy in daily life **Responsive (2)** Make sure patient has what he/she needs with regards to his/her health care Make sure patient has what he/she needs with regards to community resources	**9‐MOD** Discharge planning Complete communication of information Availability, timeliness, clarity and organization of information Medication safety Educating patients, promoting self‐management Enlisting help of social and community supports Coordinating care among team member Monitoring and managing symptoms after discharge Follow‐up with outpatient providers	Adherence to plan Satisfaction
Lenz, E. R. (2000) United States	Adults Cardiac	**Family‐focused staged psychoeducational intervention** Nurse‐led staged, postoperative psychoeducational intervention Pts viewing of the videotape in hospital and received a videotape copy of the discharge instructions for home viewing Individualized pre‐discharge counselling session with the patient and one family member Follow‐up calls every wk for 6 wks, then biweekly for the next 6 wks Group session with dietician and psychiatric nurse specialist 4 wks postdischarge Usual care included: viewing the discharge instructional videotape, handout and a home visit	**7‐LOW** **Holistic (3)** Provide interventions/services to patient that address all domains of health including physical comfort and emotional support Provide information regarding health promotion, illness prevention or lifestyle change to patient Provide information on disease and self‐management to patient **Collaborative (2)** Explain to patient the treatment options and self‐management strategies available to manage the problem Incorporate the patient and family in patient care **Responsive (2)** Identify changes in patient's condition or feeling and act upon them Comfort the patient when needed	7**‐LOW** Discharge planning Complete communication of information Availability, timeliness, clarity and organization of information Educating patients, promoting self‐management Enlisting help of social and community supports Monitoring and managing symptoms after discharge Follow‐up with outpatient providers	Perioperative complications Depressive symptoms Functional status Patient satisfaction
Naylor, M. D. (2004) United States	Older Adults (≥65 years), Cardiac	**Transitional Care intervention** 3 mth APN directed discharge planning and home follow‐up protocol Initial APN visit within 24hrs of hospital admission then daily until discharge A minimum of 8 APN home visits, first within 24hrs postdischarge, bimonthly in 2nd and 3rd mths Telephone availability 7 days a week Pt teaching was audiotaped and provided to pts and caregivers to review	**14‐MOD** **Holistic (6)** Comprehensively assess patients’ condition including physical, emotional, social and spiritual domains of health Assess patient's health values and goals Identify patient's concerns and/or needs Provide interventions/services to patient that address all domains of health including physical comfort and emotional support Provide information regarding health promotion, illness prevention or lifestyle change to patient Provide information on disease and self‐management to patient **Collaborative (5)** Explore and respect patient's beliefs about the problem and specific health concerns Promote discussion with patient to find a common understanding of what the problem is Explain to patient the treatment options and self‐management strategies available to manage the problem Provide instructions to patient on how to apply treatment option or self‐management strategy in daily life Incorporate the patient and family in patient care **Responsive (3)** Identify changes in patient's condition or feeling and act upon them Make sure patient has what he/she needs with regards to his/her health care Make sure patient has what he/she needs with regards to community resources	**9‐MOD** Discharge planning Complete communication of information Availability, timeliness, clarity and organization of information Medication safety Educating patients, promoting self‐management Enlisting help of social and community supports Coordinating care among team member Monitoring and managing symptoms after discharge Follow‐up with outpatient providers	Quality of life Functional status Satisfaction
Nucifora, G (2006) Italy	Adults Cardiac	**Heart failure management programme** Nurse‐led education programme, facilitated telephone communication and follow‐up visits with an internist at 15 days, 1 and 6 months Pre‐discharge education Telephone follow‐up by nurse 3 to 5 days after discharge Pts had telephone access to study nurse if needed Outpatient visits by internal medicine doctors at 15 days, 1 and 6 months after discharge.	**5‐LOWàHolistic (3)** Identify patient's concerns and/or needs Provide information regarding health promotion, illness prevention or lifestyle change to patient Provide information on disease and self‐management to patient **Collaborative (1)** Answer questions patient may have about his/her care **Responsive (1)** Identify changes in patient's condition or feeling and act upon them	**8‐LOW** Discharge planning Complete communication of information Availability, timeliness, clarity and organization of information Medication safety Educating patients, promoting self‐management Coordinating care among team member Monitoring and managing symptoms after discharge Follow‐up with outpatient providers	Compliance with self‐care behaviours Medication use Quality of life Clinical status
Zhao and Wong (2009) China	Older Adults (≥60 years), Cardiac	**Discharge planning intervention** Assessment, health education and consultation before discharge Discharge plan, patient referral, continued education and consultation during follow‐up after discharge Home visits within 2 days postdischarge Home visits within 2 days postdischarge and at 3 wks Telephone follow‐ups at wks and wks postdischarge	**11‐MOD** **Holistic (5)** Assess patient's understanding of the presenting problem (i.e. cause, nature) Identify patient's concerns and/or needs Monitor or reassess patient's needs Provide information regarding health promotion, illness prevention or lifestyle change to patient Provide information on disease and self‐management to patient **Collaborative (5)** Support patient decision‐making—share information in a complete and unbiased way regarding condition, prognosis, treatment Explore and respect patient's beliefs about the problem and specific health concerns Promote discussion with patient to find a common understanding of what the problem is Explain to patient the treatment options and self‐management strategies available to manage the problem Incorporate the patient and family in patient care **Responsive (1)** Make sure patient has what he/she needs with regards to community resources	**7‐LOW** Discharge planning Availability, timeliness, clarity and organization of information Educating patients, promoting self‐management Enlisting help of social and community supports Coordinating care among team member Monitoring and managing symptoms after discharge Follow‐up with outpatient providers	Self‐reported understanding of: CHD risk factors, CHD diet, CHD medication, CHD physical exercise Adherence to: health‐related lifestyle behaviour, diet, medications, physical exercise
Boter (2004) Netherlands	Adults, Stroke	**Outreach Nursing Support Program** Three nurse‐initiated telephone contacts (1 to 4; 4 to 8; and 18 to 24 weeks after discharge) and a visit to the patients in their homes (10 to 14 weeks after discharge). Educational brochures provided when required	**10‐MOD** **Holistic (3)** Identify patient's concerns and/or needs Monitor or reassess patient's needs Provide information on disease and self‐management to patient **Collaborative (5)** Promote discussion with patient to find a common understanding of what the problem is Explain to patient the treatment options and self‐management strategies available to manage the problem Answer questions patient may have about his/her care Assess patient's preferences for treatment or self‐management Provide instructions to patient on how to apply treatment option or self‐management strategy in daily life **Responsive (2)** Make sure patient has what he/she needs with regards to his/her health care Comfort the patient when needed	**5‐LOW** Discharge planning Availability, timeliness, clarity and organization of information Educating patients, promoting self‐management Monitoring and managing symptoms after discharge Follow‐up with outpatient providers	Quality of Life Anxiety Caregiver strain Caregivers social support Dissatisfaction with care
Hill, A‐*M* (2013) Australia	≥60 years, Stroke	**A tailored education package consisting of multimedia falls prevention information with trained physiotherapist follow‐up** Patients viewed video and written materials in‐hospital Patients received 2 to 5 follow‐up bedside discussion sessions with educator Follow‐up phone call by educator 2 wks after discharge	**6‐LOW** **Holistic (2)** Provide information regarding health promotion, illness prevention or lifestyle change to patient Provide information on disease and self‐management to patient **Collaborative (3)** Explore and respect patient's beliefs about the problem and specific health concerns Explain to patient the treatment options and self‐management strategies available to manage the problem Provide support, as needed, to patient for the application of treatment option or self‐management strategy in daily life **Responsive (1)** Identify changes in patient's condition or feeling and act upon them	**1‐LOW** Educating patients, promoting self‐management	Self‐perceived risk and knowledge about falls Engagement in falls prevention strategies Falls and fall injuries
Hoffmann, T. (2015) Australia	Adults Stroke	**Brief interventions for managing depression and anxiety symptoms** 2 interventions were designed with 8 face‐to‐face 1‐hr sessions (2 prior to discharge) **1‐Coping skills intervention:** Clinical psychologist delivered activities to prepare individuals for discharge and to adjust postdischarge (Five of the eight sessions were individually tailored). **2‐ Self‐management intervention:** Occupational therapist delivered individualized educational activities aimed at assisting individuals to learn problem‐solving skills, communicate with health professionals and adjust to life poststroke. **3‐ Usual care:** Usual multidisciplinary assessment and treatment including education and advice before and throughout the discharge process	**7‐LOW** **Holistic (3)** Assess patient's health values and goals Identify patient's concerns and/or needs Provide information on disease and self‐management to patient **Collaborative (2)** Promote discussion with patient to find a common understanding of what the problem is Explain to patient the treatment options and self‐management strategies available to manage the problem **Responsive (2)** Respond to patient's needs, beliefs, values and preferences Modify the type, mode of delivery or dose of treatment or self‐management strategy to be consistent with patient's needs and preferences	**3‐LOW** Discharge planning ‐Educating patients, promoting self‐management Coordinating care among team member	Stroke knowledge Depression Anxiety
Chow (2010) China	Adults, Renal	**Nurse‐led Case Management Programme** Discharge planning protocol and a standardized 6‐week nurse‐initiated telephone follow‐up regimen Participation of pts and family members in discharge planning Pre‐discharge pt assessment and individualized education programme Community nurse home visits with frequency determined by clinical judgement	**19‐HIGH** **Holistic (5)** Comprehensively assess patients’ condition including physical, emotional, social and spiritual domains of health Assess patient's health values and goals Identify patient's concerns and/or needs Provide information regarding health promotion, illness prevention or lifestyle change to patient Provide information on disease and self‐management to patient **Collaborative (10)** Support patient decision‐making—share information in a complete and unbiased way regarding condition, prognosis, treatment Explore and respect patient's beliefs about the problem and specific health concerns Promote discussion with patient to find a common understanding of what the problem is Explain to patient the treatment options and self‐management strategies available to manage the problem Answer questions patient may have about his/her care Assess patient's preferences for treatment or self‐management Provide the chosen treatment option or self‐management strategy Provide instructions to patient on how to apply treatment option or self‐management strategy in daily life Provide support, as needed, to patient for the application of treatment option or self‐management strategy in daily life Incorporate the patient and family in patient care **Responsive (4)** Respond to patient's needs, beliefs, values and preferences Modify the type, mode of delivery or dose of treatment or self‐management strategy to be consistent with patient's needs and preferences Identify changes in patient's condition or feeling and act upon them Make sure patient has what he/she needs with regards to community resources	**5‐LOW** Discharge planning Availability, timeliness, clarity and organization of information Educating patients, promoting self‐management Enlisting help of social and community supports Monitoring and managing symptoms after discharge	Quality of life
Gonçalves, N. (2016) Brazil	Adults (≥18), Burn victims	**Educational programme with telephone reinforcement** Individualized educational programme Tailored educational material regarding a variety of topics pertaining to self‐care Follow‐up phone calls every 4–6 weeks for 6 months postdischarge	**7‐LOW** **Holistic (3)** Identify patient's concerns and/or needs Monitor or reassess patient's needs Provide information on disease and self‐management to patient **Collaborative (4)** Promote discussion with patient to find a common understanding of what the problem is Explain to patient the treatment options and self‐management strategies available to manage the problem Answer questions patient may have about his/her care Provide instructions to patient on how to apply treatment option or self‐management strategy in daily life **Responsive (2)** Identify changes in patient's condition or feeling and act upon them Comfort the patient when needed	**2‐LOW** Educating patients, promoting self‐management Monitoring and managing symptoms after discharge	Perceived self‐efficacy Health status Anxiety Stress Depression
Guihan, M.(2014) United States	Adults (≥18 years), Veterans with spinal cord injury (SCI) hospitalized for severe pressure ulcers	**Multicomponent motivational interviewing (MI)/self‐management (SM) to Improve Skin Care Behaviours** 7 small group (7–10 participants) teaching sessions by phone on self‐management 8 motivational interviewing (MI) based counselling calls over 24 weeks The education control intervention was equivalent to the SM + MI intervention in regards to number, timing and delivery of sessions	**4‐LOW** **Holistic (2)** Provide information regarding health promotion, illness prevention or lifestyle change to patient Provide information on disease and self‐management to patient **Collaborative (1)** Explain to patient the treatment options and self‐management strategies available to manage the problem **Responsive (1)** Make sure patient has what he/she needs with regards to his/her health care	**2‐LOW** Educating patients, promoting self‐management Monitoring and managing symptoms after discharge	Skin care behaviours Skin status
Huang T.T. (2005) Taiwan	≥65 years, hip fracture	**Discharge planning intervention** Nurse‐led 3 mth discharge planning intervention Visited patients within 48 hr of admission and at least every 48 hr during hospitalization. Nurse home visit 3–7 days after discharge and was available by telephone seven days per week (8 a.m.–8 p.m.). Weekly follow‐up calls to pts or caregivers by nurse thereafter	**7‐LOW** **Holistic (4)** Identify patient's concerns and/or needs Monitor or reassess patient's needs Provide information regarding health promotion, illness prevention or lifestyle change to patient Provide information on disease and self‐management to patient **Collaborative (1)** Incorporate the patient and family in patient care **Responsive (2)** Make sure patient has what he/she needs with regards to his/her health care Make sure patient has what he/she needs with regards to community resources	**8‐LOW** Discharge planning Availability, timeliness, clarity and organization of information Medication safety Educating patients, promoting self‐management Enlisting help of social and community supports Coordinating care among team member Monitoring and managing symptoms after discharge ‐Follow‐up with outpatient providers	Survival Quality of life Falls
Lainscak et al. (2013) Slovenia	Adults COPD	**Discharge Coordinator Intervention** The discharge coordinator (DC) visited pt and caregiver in hospital to assess patient situation and homecare needs. Care plan communicated by DC to community care/home care nurse and other home care providers as required to ensure goal attainment Follow‐up phone call 48 hr postdischarge by DC Home visit 7–10 days postdischarge. Usual care included routine patient education with written and verbal information about COPD, supervised inhaler use, respiratory physiotherapy as indicated and disease‐related communication between medical staff with patients and their caregivers.	**6‐LOW** **Holistic (3)** Assess patient's health values and goals Identify patient's concerns and/or needs Monitor or reassess patient's needs **Collaborative (2)** Promote discussion with patient to find a common understanding of what the problem is Incorporate the patient and family in patient care **Responsive (1)** Make sure patient has what he/she needs with regards to his/her health care	**8‐LOW** Discharge planning Complete communication of information Availability, timeliness, clarity and organization of information Educating patients, promoting self‐management Enlisting help of social and community supports Coordinating care among team member Monitoring and managing symptoms after discharge ‐Follow‐up with outpatient providers	Quality of life
McCorkle, R. (2000) United States	Older Adult (≥60 years) Cancer	**Specialized home care intervention** APN led 4 wk intervention APNs telephoned patients in the intervention group within 24 hr after discharge to schedule their first visit Nurses liaised between healthcare settings, providers and patients and families, regarding the provision of technical and psychological support. APNs available by pager 24 hr a day if needed	**9‐LOW** **Holistic (5)** Comprehensively assess patients’ condition including physical, emotional, social and spiritual domains of health Identify patient's concerns and/or needs Monitor or reassess patient's needs Provide interventions/services to patient that address all domains of health including physical comfort and emotional support Provide information on disease and self‐management to patient **Collaborative (1)** Incorporate the patient and family in patient care **Responsive (3)** Modify the type, mode of delivery or dose of treatment or self‐management strategy to be consistent with patient's needs and preferences Make sure patient has what he/she needs with regards to his/her health care Make sure patient has what he/she needs with regards to community resources	**8‐LOW** Discharge planning Complete communication of information Availability, timeliness, clarity and organization of information Educating patients, promoting self‐management Enlisting help of social and community supports Coordinating care among team member Monitoring and managing symptoms after discharge Follow‐up with outpatient providers	Survival Depressive symptoms Symptom distress Enforced social dependency

The interventions varied in the number of PFCC elements that they incorporated ranging from four to 19 of the possible 27 PFCC elements (Sidani et al., 2014). Twenty‐one studies reported nine or fewer PFCC elements, six studies reported 10–18 elements and one study reported 19 items from the PFCC elements.

The interventions also varied with regard to the comprehensiveness of the transition of care. Out of the 10 critical components of an ideal transition in care (Burke et al., 2013), interventions were reported as involving 1–9 of the components. Educating patients to promote self‐management was the most commonly included component, and it was described in all 28 interventions. Discharge planning was described in 24 trials, followed by availability, timeliness clarity and organization of information (*N* = 23) and monitoring and managing symptoms after discharge (*N* = 23). Follow‐up with outpatient providers was included in 16 trials, coordinating care among team members (*N* = 13) and enlisting help of social and community supports were mentioned in 13 trials and complete communication of information was reported in 10 trials. Medication safety was reported in 8 trials and advance care planning was not described in any studies included in this review. Finally, thirteen studies (*N* = 13) included family and caregivers in the intervention either by targeting education, ensuring their presence, assessing their needs or connecting with them during follow‐ups.

### 4.3 Patient‐oriented outcomes

Patient‐oriented outcomes are reported in Table 2. Overall, 10 out of the 28 studies (35.7%) examined the relationship between PFCC transition interventions and self‐care behaviours, 23 out of the 28 studies (82.1%) examined the link between PFCC transition interventions and health outcomes/ health status (quality of life, condition‐specific health status, depression and anxiety/stress, falls, caregiver related measures), 11 out of the 28 studies (39.3%) looked at the link between PFCC and satisfaction with care and 6 out of the 28 (21.4%) studies examined the relationship between PFCC transition interventions and condition‐specific knowledge or self‐efficacy.

**Table 2:** Patient‐Oriented Outcomes

Study/year/location	Population/Sample size	Knowledge (self‐efficacy)	Self‐care behaviours	Health outcomes/Health Status	Satisfaction
Bostrom (1996) United States	Adult NIC: 445 PIC: 494 C: 474				**Satisfaction with healthcare education (30days)** No significant differences in the mean scores between the three groups in any of the categories: medications (*F*=0.508, *p *> .5), activities of daily living (*F*=0.063, *p *> .9), community and follow‐up (*F*=0.030, *p *> .9), feelings related to condition (*F*=0.862, *p *> .4), treatment and complications (*F*=0.260, *p *> .5), enhancing quality of life (*F*=0.530, *p *> .5) and skin care (*F*=0.540, *p *> .5)
Kangovi, S (2014) United States	Adults (18–64 years); uninsured or insured by Medicaid, general medicine ward I: 222 C: 224		**Patient activation (2weeks)** Intervention patients were more likely to report improved (3.4 versus 1.6; *p* = .05). **Medication adherence (2weeks)** No differences (63.7% versus 59.3%, *p *= .37)	**Mental health (2weeks)** Intervention patients reported higher improvement in mental health (6.7 versus 4.5; *p* = .02) **Physical health (2weeks)** No differences in physical health (5.5 versus 4.8, *p *= .62)	**Discharge communication (2weeks)** Higher quality discharge communication in intervention group (91.3% versus 78.7%; *p *= .002; adjusted OR, 2.94; 95% CI, 1.5–5.8) **Satisfaction with medical care (2weeks)** No differences (3.4 versus 3.4; *p *= .85)
Naylor, M. D. (1999) United States	Adults, High risk for poor postdischarge outcomes I: 177 C: 186			**Functional status** No significant group differences (I: 22.4, *SD* = 8.1, C:22.6, *SD* = 8.4, *p *= .86) **Depression** No significant group differences (I: 12.1, *SD* = 10, C:10.7, *SD* = 9.8, *p *= .26)	**Satisfaction (2, 6, 12, 24weeks)** No significant group differences (*p *= .92)
Altfeld, S.J. (2013) United States	Older Adult I: 360 C: 360			**Patient stress (30days)** No significant differences between groups (OR = 0.85) **Caregiver stress (30days)** No significant differences between groups (OR = 1.02) **Patient assessment of health (30days)** No significant differences between groups (OR = 1.27)	**Physician communication survey (30days)** Intervention group significantly more likely to have communicated with their physicians (90.3% versus 81.9%, *p *= .002)
Chan (2015) United States	Older Adult (≥55) I: 347 C:352				**Quality of the transitional care experience (30days)** No statistically significant differences between the intervention and control groups on the CTM−3 scores (80.5% versus 78.5%; *p* = .18) **Discharge communication (30days)** No differences on HCAHPS discharge communication scores (74.8% versus 68.7%; *p* = .11).
Courtney (2009) Australia	Older Adults (≥65), At‐risk for re‐admission I: 64 C:64			**Quality of life (4weeks, 12weeks, and 24weeks)** Intervention group had better Quality of life scores Physical Component Summary scores (*F* (3,279) = 30.43, *p *< .001) Mental Component Summary scores (*F* (3,279) = 7.20, *p *< .001)	
Li, H. (2012) United States	Adults (≥21 years) who were primary caregivers for a hospitalized older adult (≥65 years) I: 202 C:205			**Depressive symptoms (2weeks and 2months)** No differences between groups 2weeks (I: 2.77 versus C: 2.33, *p *= .20) and 2months (I: 2.06 versus C: 2.03, *p *= .90) **Patient's cognitive status (2w and 2mo)** No differences between groups 2weeks (I: 2.27 versus C: 1.79, *p *= .19) and 2months (I: 2.21 versus C: 1.68, *p *= .10) **Functional status (2weeks)** No differences between groups (I: 25.47 versus. C: 24.12, *p *= .21)	
Cajanding (2017) Philippines	Adult Cardic I: 75 C: 68	**Cardiac self‐efficacy (30d post)** Statistically significant improvement in cardiac self‐efficacy in intervention group The mean difference in cardiac self‐efficacy scores was 5.61 ± 1.13 (95% CI, 7.87 ± 3.36); *p* < .01		**Functional status (30days)** Statistically significant improvement of functional status in intervention group The mean difference in perceived functional status scores was 8.59 ± 2.29 (95% CI, 4.02 ± 13.16); *p* < .01	**Satisfaction (30days)** Statistically significant higher satisfaction in intervention group The mean difference in satisfaction scores was 17.33 ± 2.73 (95% CI, 22.78 ± 11.89); *p* < .01
Davis,K.K. (2012) United States	Adults ( ≥21) Cardiac I: 63 C: 62	**Heart failure knowledge (30days)** Significant increase in intervention group (mean difference = 0.04, *SD* = 1.69, *p* < .001)	**Heart failure self‐care (30days)** No differences on the Self‐Care maintenance (mean difference = −13.75, *SD* = 17.78, *p* = .711), management (mean difference = −3.75, *SD* = 21.44, *p* = .430) or confidence (mean difference = 0.55, *SD* = 17.86, *p* = .692)	**Depression (30days)** No difference in depressive symptoms in both groups (I: meaN = 52% versus. C: meaN = 56%; *p* = .65)	
Hanssen, T. A (2007) Norway	Adults Cardiac I: 156 C: 132		**Physical activity (6 months)** Intervention group reported more frequent physical activity (*p* = .004). No differences found at 3mo. **Smoking cessation (6 months)** No difference in smoking cessation (I: 60%, versus C: 40.8%, *p* = .055)	**Quality of life (6 months)** No differences on quality of life scores between groups except for a significant improvement in the physical health component (I: 7.31, *SD* = 7.46, C: 4.98, *SD* = 8.10, *p* = .039) in favour of the intervention group	
Harrison, M. B. (2002) Canada	Adults Cardiac I: 92 C:100			**Quality of life (heart failure) (6weeks and 12weeks)** Improved scores with intervention patients at 6w (I: 27.2, *SD* = 19.1, C: 37.5, *SD* = 20.3, *p* = .002) and 12 w (I: 25.8, *SD* = 19.4, C: 38.4, *SD* = 18.2, *p* < .001) **Generic quality of life** Improved mental health component of SF−36 at 6 w, (I: 53.46, *SD* = 10.50, C: 49.53, *SD* = 11.29, *p* = .05) No differences were found on the physical component at 6weeks (*p* = .82) and 12weeks (*p* = .07) and on the general health at 6weeks (*p* = .07) and 12weeks (*p* = .434)	
Holmes‐Rovner, M. (2008) United States	≥ 21 years Cardiac I: 268 C:257		**Physical activity (3months)** Intervention group showed higher self‐reported physical activity (OR = 1.53; *p *= .01) during the first three months, with decline after active intervention was withdrawn. **Weight loss (3months)** No differences (OR = 1.08; *p *> .05) **Smoking cessation (3months)** No differences (OR = 2.34; *p *> .05)	**Functional status (8months)** No differences (I: 29.12 versus C: 30.00; *p* = .55) **Quality of life (8months)** No differences (I: 0.75 versus C: 0.74; *p* = .55) at 8mo	
Jaarsma, T. (2000) Netherlands	≥50 years Cardiac I: 55 C: 73		**Heart Failure Self‐care Behaviour (1month and 3months)** Significant increase in self‐care behaviour in intervention group at 1month (I: 14.2, *SD* = 2.9, C: 12.2, *SD* = 2.9, *p* < .001) and at 3month (I: 12.2, *SD* = 3.1, C: 10.6, *SD* = 3.2, *p* = .005). No differences at 9months follow‐up (*p *= .11)		
Jerant et al. (2003) United States	≥40 years Cardiac 25 (Home telecare: 13, telephone: 12, control: 12)			**Quality of life (2months)** No statistically significant differences (*p *= .39)	**Satisfaction (2months)** No statistically significant differences (*p *= .40)
Laramee, A.S. (2003) United States	Cardiac I: 122 C:112		**Adherence to plan (4weeks and 12weeks)** The intervention group adhered to the treatment plan better than the usual care group with regard to daily weights (I: 4.6 versus C: 3.1, *p *< .001), checks for oedema (I: 4.8 versus C: 4.6, *p *= .02), low‐salt diet (I: 4.8 versus C: 4.4, *p *< .001) and fluid recommendation (I: 5.0 versus C: 4.6, *p *= .003) **Medication adherence** No differences found for medication adherence (*p *= .04).		**Satisfaction (4weeks)** Intervention group more satisfied with care (I: 4.2 versus C: 3.8, *p* < .001)
Lenz, E. R. (2000) United States	Cardiac I: 19 C: 19			**Perioperative complications (3−4days, 2weeks, 4weeks, 6weeks, and 12weeks)** No differences between groups (*F*(1,36)= 4.61, *p* < .04) **Depressive symptoms (3−4days, 2weeks, 4weeks, 6weeks, and 12weeks)** No differences between groups (*F*(1,34) =0.01, *p *< .91) **Functional status (3−4days, 2weeks, 4weeks, 6weeks, and 12weeks)** No differences between groups (*F*(1,36)=0.09, *p *< .67)	**Patient satisfaction (3−4days, 2weeks, 4weeks, 6weeks, and 12weeks)** No differences between groups (*F*(1,35) =1.87, *p *< .18)
Naylor, M. D. (2004) United States	Older Adults (≥65 years), Cardiac I: 118 C: 121			**Quality of life** The intervention group reported greater overall quality of life at 12 weeks (I: 3.2, *SD* = 1.5, C: 2.7, *SD* = 1.5, *p *< .05) and in the physical dimension at 2 weeks (I: 3.5, *SD* = 1.2, C: 3.0, *SD* = 1.2, *p *< .01) and 12 weeks (3.6, *SD* = 1.4, C: 3.1, *SD* = 1.6, *p *< .05) No differences between groups at 6, 26 and 52weeks. **Functional status** Statistically significant group differences in functional status did not emerge, although less dependency was, on average, observed	**Satisfaction with care** Satisfaction with care was greater in intervention patients at 2 and 6 weeks (83.1, *SD* = 9.6, C: 77.8, *SD* = 11.2, *p *< .001)
Nucifora, G (2006) Italy	Adults Cardiac I: 74 C: 75		**Compliance with self‐care behaviours (6months)** No difference between groups (*p *> .05) **Medication use (6months)** No differences between groups (I: 87% versus C: 84%, *p *> .05)	**Quality of life (6months)** No differences in groups (I: 14, *SD* = 20, C: 10, *SD* = 16, *p *> .05) **Clinical status (6months)** No differences between groups (*p *> .05)	
Zhao and Wong (2009) China	Older Adults (≥60 years), Cardiac I: 100 C: 100	**Self‐reported understanding of congenital heart disease (CHD) risk factors** Improved for intervention group between 2days and 4weeks after discharge, *p < .01* No differences between 4weeks and 12weeks. **Self‐reported understanding of CHD diet** Higher for intervention group at 2days, 4weeks and 12weeks postdischarge, *p* < .001. Knowledge level increased between follow‐ups for intervention group, *p* < .01. **Self‐reported understanding of CHD medication** Increased more among intervention group at 2days, 4weeks and 12weeks, *p < .001* **Self‐reported understanding of CHD physical exercise** Increased in intervention group at 12weeks, *p < .001*. No differences at 2days and 4weeks follow‐up.	**Health‐related lifestyle behaviour compliance** Intervention group reported better at 2days, *p = .026*, 4weeks, *p < .001* and 12weeks, *p < .001* **Self‐reported diet adherence** Better in intervention group at 2days, *p = .003*, 4weeks, *p < .001* and 12weeks, *p = .023* **Medication adherence** Intervention patients reported better at 4weeks, *p = .012* and 12weeks, *p < .001*. No difference at 2days. **Physical exercise compliance** Intervention patients reported better at 12weeks, *p < .001*. No group differences at 2days and 4weeks.		
Boter (2004) Netherlands	Adult Stroke I: 231 C: 255			**Quality of life (6months)** No statistically significant differences except for higher scores in intervention group on the SF−36 domain “Role Emotional” (mean difference = 7.9; 95% CL, 0.1 to 15.7) **Anxiety (6months)** Lower anxiety scores in intervention group Hospital depression subscale: Difference between medians = 1 (−0.52 to 2.98) Hospital anxiety subscale: Difference between medians = 1 (0.19 to 2.79) **Carers’ strain (6months)** No statistically significant differences between the 2 groups Difference between medians = 0 (−1.01 to 1.53) **Carers’ social support (6months)** No statistically significant differences between the 2 groups Difference between medians = 0 (−1.20 to 1.20)	**Dissatisfaction with stroke care (6months)** No differences in satisfaction with care in hospital or after discharge. Hospital subscale (RR = 1.17) and home subscale (RR 1.07)
Hill, A‐*M* (2013) Australia	≥60 years, Stroke or Rehabilitation units I: 25 C:25	**Self‐perceived risk and knowledge about falls (1month)** Intervention group had a significantly increased self‐perceived risk of falls [OR 4.96, 95% CI (2.84, 7.10), *p* < .001] and of falls injuries [OR 4.76, 95% CI (2.59, 6.94), *p* < .001]	**Engagement in falls prevention strategies (1month)** Intervention group was more likely to seek formal assistance for ADL [Adjusted OR 3.02, 95% CI (0.82, 11.10), *p* = .09] and IADL [AOR 2.53, 95% CI (0.75, 8.59), *p* = .14], plan to gradually resume functional activities [AOR 3.80, 95% CI (1.07, 13.52), ***p* = .04**], participate in their own home exercise programme [AOR 2.76, 95% CI (0.72, 10.50), *p* = .14] and make their own informal home modifications [AOR 2.43, 95% CI (0.74, 7.96), *p* = .14], **although only one comparison reached statistical significance.**	**Falls and fall injuries (1month)** Intervention group had less falls (I: 5.4/1000 and C: 18.7/1000) [Adjusted OR 3.38, 95% CI (0.98, 11.56), *p* = .05] and fall injuries (I: 2.2/1000 and C: 10.4/1000) [Adjusted OR 4.42, 95% CI (0.66, 29.54), *p* = .12]	
Hoffmann, T. (2015) Australia	Adults Stroke 23 (Coping skills: *N* = 11; Self‐management: *N* = 12) C:10	**Stroke knowledge (3months)** No difference on stroke self‐efficacy or knowledge		**Depression** No difference at 1week and 3months. **Anxiety** No difference at 1week and 3months.	
Chow (2010) China	Adults, Dialysis I: 43 C: 42			**Kidney disease quality of life (6weeks and 12weeks)** Statistically significant within‐group effects were found for symptoms/problems (*F* = 4.5, *p *= .01), effects of kidney disease (*F* = 3.28, *p *= .04), sleep (*F* = 3.46, *p *= .03), role‐physical (*F* = 6.95, *p *= .00), pain (*F* = 3.17, *p *= .05), emotional wellbeing (*F* = 4.35, *p *= .01) and social function (*F* = 7.95, *p *= .00). Statistically significant interaction effects were demonstrated for staff encouragement (*F* = 5.21, *p *= .01), patient satisfaction (*F* = 3.62, *p *= .03), sleep (*F* = 9.47, *p *= .00) and social function (*F* = 4.62, *p *= .01)	
Gonçalves, *N*. (2016) Brazil	Adults (≥18), Burn victims I: 43 C:46	**Perceived self‐efficacy (6months)** No differences on the Perceived Self‐Efficacy Scale (I: 41.3, *SD* = 6.4, C: 40.4, *SD* = 7.4, *p* = .53)		**Health status (6months)** No differences in perceived health status (I: 129.8, *SD* = 18.5, C: 127.0, *SD* = 22.5, *p* = .52) **Anxiety (6months)** Intervention patients had lower anxiety score (I: 4.6, *SD* = 3.5, C: 6.7, *SD* = 5.0, *p = .03)* **Stress (6months)** Lower scores on stress in intervention group (I: 49.5, *SD* = 35.3, C: 69.2, *SD* = 39.5, *p < .02)*. **Depression (6months)** No differences between groups in depression scores (I: 3.8, *SD* = 4.1, C: 4.4, *SD* = 4.4, *p* = .48)	
Guihan, M.(2014) United States	Adults (≥18 years), Ulcers I: 71 C:72		**Skin care behaviours (6 months)** No differences in adherence (I: 85, *SD* = 15.2, C: 83, *SD* = 14.6, *p* = .41)	**Skin status (6 months)** No differences between groups on skin status (I: meaN = 50.7% versus C: meaN = 45.8%, *p* = .86)	
Huang T.T. (2005) Taiwan	≥65 years, hip fracture due to falling I: 63 C: 63			**Survival (3months)** Higher survival rate among intervention group (I: 3, *SD* = 0, C: 2.92, *SD* = 0.04, *p* = .04) **Quality of life (3months)** Higher QOL in intervention group (I: 60.8, *SD* = 10.5 versus. C: 51.3, *SD* = 11.6). Group by time interaction was significant (*F* = 4.06, *p* < .05) and the main effects of group and time were significant (*F* = 21.77 and 289.47, *p* < .01). **Falls** No significant difference was found in the average time to repeat fall experienced by patients in either group (2.83 versus. 2.79 mo after discharge, *p *= .57)	
Lainscak et al. (2013) Slovenia	COPD I: 118 C:135			**Quality of life (6months)** No differences between the groups (*p *= .774)	
McCorkle, R. (2000) United States	Older Adult (≥60 years) Cancer I: 190 C:185			**Survival** The risk of death was approximately doubled among usual care patients (adjusted hazard ratio 2.04; CI, 1.33–3.12; *p *= .001) compared with those in the intervention group. **Depressive symptoms (6months)** No differences between groups (I: 9.70, *SD* = 8.5, C: 9.10, *SD* = 7.5, *p *> .01) **Symptom distress (6months)** No differences between groups (I: 21.01, *SD* = 7.4, C:19.58, *SD* = 6.2, *p *> .01) **Enforced social dependency (6months** **)** No difference between groups (I: 18.85, *SD* = 8.0, C: 16.94, *SD* = 6.9, *p *> .01)	

### 4.4 Self‐care behaviours

Self‐care was an outcome in 10 out of the 28 studies (35.7%). Out of this sub‐group of 10 studies, three out of three studies that measured patient compliance with their physical exercise plan found a significant difference (*p* = .004, Hanssen et al., 2007; *p* = .01, Holmes‐Rovner et al., 2008), *p* < .001 (Zhao & Wong, 2009)). Only 1 of 4 studies that measured medication adherence found a significant improvement (*p* = .012, Zhao & Wong, 2009). The other 3 studies that measured medication adherence found no differences (*p* = .37, Kangovi et al., 2014; *p* = .04, Laramee et al., 2003; *p* > .05, Nucifora et al., 2006).

Other studies out of this sub‐group found a significant improvement in other self‐care measures: (1) patient activation (*p* = .05, Kangovi et al., 2014); (2) heart failure self‐care (*p* < .001, Jaarsma et al., 2000); (3) adherence to plan (*p* < .001, Laramee et al., 2003); (4) health‐related lifestyle behaviour compliance (*p* < .001, Zhao & Wong, 2009); (5) diet adherence (*p* = .003, Zhao & Wong, 2009); and (6) engagement in falls prevention strategies (*p* = .04, Hill, Etherton‐Beer, Haines, 2013). However, some studies found no significant differences in self‐care measures: (1) heart failure self‐care (*p* = .711, Davis et al., 2012); (2) smoking cessation (*p* = .055, Hanssen et al., 2007), *p* > .05, Holmes‐Rovner et al., 2008); (3) weight loss (*p* > .05, Holmes‐Rovner et al., 2008); (4) compliance with self‐care behaviours (*p* > .05, Nucifora et al., 2006); and (5) skin care behaviours (*p* = .41, Guihan et al., 2014).

### 4.5 Health outcomes/health status

A sub‐group of 23 out of the 28 studies (82.1%) reported a health outcome or health status measure. Eleven studies out of the 23 assessed the patients’ quality of life. Out of these 11, only five studies showed a significant improvement in quality of life (*p* < .001, Courtney et al., 2009); *p* = .002 (heart failure), Harrison et al., 2002; *p* < .05, Naylor et al., 2004; *p* = .01 (kidney disease), Chow & Wong, 2010; *p* < .05, Huang and Liang, 2005) (Table 3).

**Table 3:** Association between PFCC transition interventions and quality of life (*N* = 11)

Study/year/location	Significance	Direction and Magnitude	Comment
Courtney (2009) Australia	Significant	+	*p* < .001
Hanssen, T. A (2007) Norway	Non‐significant	−	No differences on quality of life scores between groups
Harrison, M. B. (2002) Canada	Significant	+	Heart failure quality of life *p* = .002
Holmes‐Rovner, M. (2008) United States	Non‐significant	−	*p* = .55
Jerant et al. (2003) United States	Non‐significant	−	*p* = .39
Naylor, M. D. (2004) United States	Significant	+	*p* < .05
Nucifora, G (2006) Italy	Non‐significant	−	*p* > .05
Boter (2004) Netherlands	Non‐significant	−	No statistically significant differences
Chow (2010) China	Significant	+	Kidney disease quality of life *p* = .01
Huang T.T. (2005) Taiwan	Significant	+	*p* < .05
Lainscak et al. (2013) Slovenia	Non‐significant	−	*p* = .774

Also, some studies showed a significant improvement in other health outcomes: (1) mental health (*p* = .02, Kangovi et al., 1999); (2) functional status (*p* < .01, Cajanding et al., 2017); (3) falls (*p* = .05, Hill et al., 2013); and (4) survival (*p* = .04, Huang & Liang, 2005), (*p* = .001, McCorkle et al., 2000). Other studies showed no significant differences in: (1) functional status (*p* = .86, Naylor et al., 1999), (*p* = .55, Holmes‐Rovner et al., 2008), (*p* < .67, Lenz & Perkins, 2000); (2) patient stress (OR = 0.85) or caregiver stress (OR = 1.02) (Altfeld et al., 2013); (3) depressive symptoms (*p* = .20, Li et al., 2012), (*p* = .65, Davis et al., 2012), (Hoffmann et al., 2015); (4) health status (*p* = .52, Goncalves et al., 2016); and (5) skin status (*p* = .86, Guihan et al., 2014).

### 4.6 Satisfaction related measures

Patient satisfaction related measures were assessed in 11 out of the 28 studies (39.2%). Out of these 11 studies, seven measured overall satisfaction. Only 3 of the 7 studies that assessed overall satisfaction showed a significant improvement (*p* < .01, Cajanding, 2017; *p* < .001, Laramee et al., 2003; *p* < .001, Naylor et al., 2004) (Table 4).

**Table 4:** Association between PFCC transition interventions and overall satisfaction (*N* = 7)

Study/year/location	Significance	Direction and Magnitude	Comment
Kangovi, S (2014) United States	Non‐significant	−	*p* = .85
Naylor, M. D. (1999) United States	Non‐significant	−	*p* = .92
Cajanding (2017) Philippines	Significant	+	*p* < .01
Jerant et al. (2003) United States	Non‐significant	−	*p* = .40
Laramee, A.S. (2003) United States	Significant	+	*p* < .001
Lenz, E. R. (2000) United States	Non‐significant	−	*p* < .18
Naylor, M. D. (2004) United States	Significant	+	*p* < .001

Other studies showed a significant improvement in: (1) discharge communication (*p* = .002, Kangovi et al., 2014); and (2) physician communication (*p* = .002, Altfeld et al., 2013) while other studies found no significant differences in: (1) satisfaction with healthcare education (*p* > .5, Bostrom et al., 1996); (2) quality of the transitional care experience (*p* = .18, Chan et al., 2015); and (3) satisfaction with stroke care (Boter et al., 2004).

### 4.7 Condition‐specific knowledge/self‐efficacy

Condition‐specific knowledge or self‐efficacy was assessed in six out of the 28 studies (21.4%), but each of the six studies measured a different outcome. Four of the six studies found a significant improvement in knowledge: (1) cardiac self‐efficacy (*p* < .01, Cajanding et al., 2017); (2) heart failure knowledge (*p* < .001, Davis et al., 2012),; (3) understanding of congenital heart disease (CHD) risk factors, diet, medication, physical exercise (*p* < .001, Zhao & Wong, 2009); and (4) self‐perceived risk and knowledge about falls (*p* < .001, Hill et al., 2013). Two of the six studies found a non‐significant difference in: (1) stroke knowledge (Hoffman et al., 2015); and (2) perceived self‐efficacy (*p* = .53, Goncalves et al., 2016).

### 4.8 Risk of Bias

Risk of bias varied among the included studies. Ten out of the 28 studies (35.7%) showed low risk of bias on all of the domains, 11 out of 28 studies (39.3%) showed high risk of bias on at least one domain and seven out of the 28 studies (25.0%) had insufficient information to determine bias in at least one of the domains. Methodological limitations included sequence generation (*N* = 2), blinding (*N* = 7), incomplete outcome data (*N* = 3), selective outcome reporting (*N* = 2) and other sources of bias (*N* = 1). See Table 5.

**Table 5:** Risk of bias assessment

Study	Sequence generation	Allocation Concealment	Blinding of participants and personnel	Blinding of outcome assessors	Incomplete outcome data	Selective outcome reporting	Other sources of bias
Altfeld	Low	Unclear	Unclear	Unclear	Low	Low	Low
Bostrom	High	Unclear	Unclear	Low	Low	Low	High
Boter	Low	Low	Low	Low	Low	Low	Low
Cajanding	Low	Low	Low	Low	Low	Low	Low
Chan	Low	Low	Unclear	Unclear	Low	Low	Low
Chow	Low	Unclear	High	Unclear	Low	Low	Low
Courtney	Low	Low	Low	Low	Low	Low	Low
Davis	Low	Low	Low	Low	Low	Low	Low
Gonçalves	Low	Low	Low	High	Low	Low	Low
Guihan	Low	Low	Low	Low	Low	Low	Low
Hanssen	Low	Low	Low	Low	High	Low	Low
Harrison	Low	Low	Low	Low	Low	Low	Low
Hill	Low	Low	Low	Low	Low	Low	Low
Hoffman	Low	Low	Low	Low	Low	Low	Low
Holmes‐Rovner	High	Low	Low	Low	Low	Low	Low
Huang	Low	Low	Unclear	Low	Low	Low	Low
Jaarsma	Low	Low	High	High	Low	Low	Low
Jerant	Low	Low	High	High	High	High	Low
Kangovi	Low	Low	Low	Low	Low	Low	Low
Lainscak	Low	Low	High	Low	Low	Low	Low
Laramee	Low	Unclear	High	Low	Low	Low	Low
Lenz	Low	Low	Unclear	High	Low	Low	Low
Li	Low	Low	Unclear	High	Low	Low	Low
McCorkle	Low	Low	Unclear	Unclear	Low	Low	Low
Naylor	Low	Low	Unclear	Low	Low	Low	Low
Naylor	Low	Low	Unclear	Low	Low	Low	Low
Nucifora	Low	Low	High	High	Low	Low	Low
Zhao	Low	Low	High	Low	High	High	Low

## 5 DISCUSSION

Multifaceted PFCC transition interventions (*N* = 28) had mixed effects on patient‐oriented outcomes. Interventions varied in regards to the extent of the PFCC focus and the comprehensiveness of the transition of care. Educating patients to promote self‐management was the most commonly included component and it was described in all 28 interventions. Studies included multiple patient‐oriented outcomes. Overall, outcome categories included self‐care behaviours (*N* = 10), health outcomes/ health status (*N* = 23), satisfaction with care (*N* = 11), condition‐specific knowledge or self‐efficacy (*N* = 6). Methodological limitations were identified in 11 (39%) of the included trials.

Our SR reported considerable heterogeneity in intervention types and outcomes among the studies examined, similarly to other SRs (Allen et al., 2014; Black & Duval, 2019; Desai et al., 2015; Dusek et al., 2015). Allen et al. (2014) included twelve studies (*N* = 12) which consisted of a wide variety of transitional care interventions with half (*N* = 6) the studies measuring a patient‐oriented specific outcome such as patient satisfaction. In Desai et al. (2015)’s review, which included a mix of paediatric (*N* = 4) and adult (*N* = 12) trials of emergency department to home transition interventions showed a clear gap of the impact of PFCC transition on patient‐oriented outcomes. Results from two other SRs (Black & Duval, 2019; Dusek et al., 2015) demonstrated the need for a more PFCC approach to ensure that effective information exchange among patients, caregivers and clinicians occurs during care transitions (Dusek et al., 2015), to improve patient outcomes for diabetes patients during care transitions (Black & Duval, 2019).

Although PFCC has gained momentum worldwide, the biggest challenge is meaningfully engaging patient and family in the different elements of PFCC included in the holistic care, collaborative care and responsive care components (Sidani et al., 2014). In our review, all PFCC elements (Sidani et al., 2014) or all the critical components of an ideal transition (Burke et al., 2013) were not considered across the different multifaceted interventions in the included studies. For successful achievement of PFCC care transitions, clinicians, administrators and other stakeholders need to incorporate all the elements of PFCC into care transition interventions. A framework should be used to clearly define the interventions to better determine their effectiveness.

Guidelines and policies that promote PFCC during transitions of care include a lack of clearly defined standards and protocols to provide PFCC and a lack of communication guidelines that foster patient and provider communication. Systems must be designed to deliver consistent, PFCC results during transitions of care between settings. Organizations need to consider PFCC as a partnership with all stakeholders including clinicians working in different healthcare sectors, patients and caregivers. There should be guidelines and policies in place to support all stakeholders and to hold accountable those organizations that fail to design such systems. Future research should focus on understanding the relationship between PFCC transition interventions including the specific PFCC elements and also the components of the “ideal transition” on patient‐oriented outcomes.

### 5.1 Strengths and limitations

Despite the systematic and rigorous methods used in this review, there are limitations. We only searched some of the grey literature databases and, as such, this review may not contain all the work done on this topic. Furthermore, only RCTs were included in this study. Since the review included a variety of PFCC transition interventions and patient‐oriented outcome measures found, we used a vote‐counting approach to synthesize the data. Vote counting has its limitations as it does not take into account the differential weights given to each study and it does not demonstrate an estimate of the effect size (Grimshaw et al., 2003).

## RELEVANCE TO CLINICAL PRACTICE

In this systematic review, we critically analysed the effect of PFCC transition interventions on patient‐oriented outcomes and we identified gaps in the literature that could inform future intervention studies and that could aid in the development of measures to determine the effectiveness of care transitions from hospital to home.

## CONFLICT OF INTEREST

None declared.

## AUTHORSHIP

CB was a major contributor in writing the manuscript. CB and JC designed the project, performed data acquisition, analysis and interpretation. OD designed and ran the literature searches. MC, RD participated in the data collection. CB, JC and BS conducted the data analyses. All co‐authors critically appraised and edited the manuscript. All authors also read and approved the final manuscript.

## Supporting information

Fig S1

Table S1

Table S2

Table S3

## Data Availability

All data generated or analysed during this study is available in the manuscript.
