# Supplementary material for: Effectiveness of person‐ and family‐centred care transition interventions on patient‐ oriented outcomes: A systematic review
Source: Nurs Open. 2020 Nov 19;8(2):721–54. doi: 10.1002/nop2.677 (PMC7877224; doi:10.1002/nop2.677)
Supplement: Supplementary file 1 — Fig S1 [file NOP2-8-721-s001.doc]

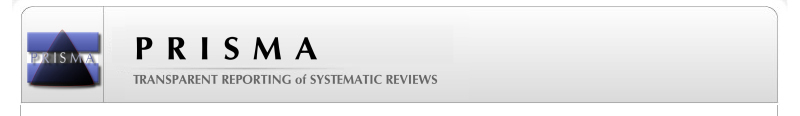
 **PRISMA Diagram**

**Screening**

**Included**

**Eligibility**

**Identification**

References identified through database searching
(n = 6,886)

References screened against title and abstract
(n = 6,127)

References excluded
(n = 5,571)

Full-text articles assessed for eligibility
(n = 556)

Full-text articles excluded

(n = 528)

- Not a person-family centred care transition intervention (n = 341)
- Not related to care transitions (n = 47)
- Wrong study design (n = 71)
- No full text (n = 5)
- Wrong outcomes (n = 19)
- Wrong patient population (n=30)
- Wrong setting (n=6)
- Non-English or Non-French (n = 9)

Studies included

(n = 28)

Duplicates removed
(n = 759)
