# Supplementary material for: Effectiveness of person‐ and family‐centred care transition interventions on patient‐ oriented outcomes: A systematic review
Source: Nurs Open. 2020 Nov 19;8(2):721–54. doi: 10.1002/nop2.677 (PMC7877224; doi:10.1002/nop2.677)
Supplement: Supplementary file 3 — Table S2 [file NOP2-8-721-s003.docx]

**S2 Table. MEDLINE Search Strategy**

1. exp "continuity of patient care"/

2. (continu* adj3 care).ti,ab,kw,kf,hw.

3. discharge*.ti,ab,kw,kf,hw.

4. handoff*.ti,ab,kw,kf,hw.

5. hand off*.ti,ab,kw,kf,hw.

6. handover*.ti,ab,kw,kf,hw.

7. hand over*.ti,ab,kw,kf,hw.

8. signoff*.ti,ab,kw,kf,hw.

9. sign off*.ti,ab,kw,kf,hw.

10. signover*.ti,ab,kw,kf,hw.

11. sign over*.ti,ab,kw,kf,hw.

12. transfer*.ti,ab,kw,kf,hw.

13. transition*.ti,ab,kw,kf,hw.

14. (turf* adj3 patient*).ti,ab,kw,kf,hw.

15. (dump* adj3 patient*).ti,ab,kw,kf,hw.

16. posthospital*.ti,ab,kw,kf,hw.

17. post hospital*.ti,ab,kw,kf,hw.

18. or/1-17

19. home.ti,ab,kw,kf,hw.

20. exp home care services/

21. (domicil* adj3 care).ti,ab,kw,kf,hw.

22. patient readmission/

23. readmi*.ti,ab,kw,kf,hw.

24. rehospitali*.ti,ab,kw,kf,hw.

25. post discharge.ti,ab,kw,kf,hw.

26. or/19-25

27. exp patient-centered care/

28. (patient* adj3 (centred* or centered* or focus*)).ti,ab,kw,kf,hw.

29. (person adj3 (centred* or centered* or focus*)).ti,ab,kw,kf,hw.

30. (famil* adj3 (centred* or centered* or focus*)).ti,ab,kw,kf,hw.

31. patient participation/

32. (patient* adj3 (involve* or empower* or participat* or activat* or engage* or perspective*)).ti,ab,kw,kf,hw.

33. patient education as topic/

34. (patient* adj3 educat*).ti,ab,kw,kf,hw.

35. quality improvement/

36. quality improvement.ti,ab,kw,kf,hw.

37. (intervent* adj3 (care or patient*)).ti,ab,kw,kf,hw.

38. or/27-37

39. 18 and 26 and 38

40. remove duplicates from 39
