# Supplementary material for: Effectiveness of person‐ and family‐centred care transition interventions on patient‐ oriented outcomes: A systematic review
Source: Nurs Open. 2020 Nov 19;8(2):721–54. doi: 10.1002/nop2.677 (PMC7877224; doi:10.1002/nop2.677)
Supplement: Supplementary file 4 — Table S3 [file NOP2-8-721-s004.docx]

**S3 Table. Full Text Articles Excluded with Reasons (n=528)**

**Wrong intervention (n=341)**

| **Authors** | **Year** | **Title** | **Journal** | **Volume** | | **Issue** | **Pages** |
| --- | --- | --- | --- | --- | --- | --- | --- |
| Anderson, S. L.; Marrs, J. C.; Vande Griend, J. P.; Hanratty, R. | 2013 | Implementation of a clinical pharmacy specialist-managed telephonic hospital discharge follow-up program in a patient-centered medical home | Population Health Management | 16 | | 4 | 235-41 |
| Abad-Corpa, E.; Royo-Morales, T.; Iniesta-Sanchez, J.; Carrillo-Alcaraz, A.; Rodriguez-Mondejar, J. J.; Saez-Soto, A. R.; Vivo-Molina, M. C. | 2013 | Evaluation of the effectiveness of hospital discharge planning and follow-up in the primary care of patients with chronic obstructive pulmonary disease | Journal of Clinical Nursing | 22 | |  | 669-80 |
| Adams, C. J.; Stephens, K.; Whiteman, K.; Kersteen, H.; Katruska, J. | 2014 | Implementation of the Re-Engineered Discharge (RED) toolkit to decrease all-cause readmission rates at a rural community hospital | Quality Management in Health Care | 23 | | 3 | 169-77 |
| Addington-Hall, J. M.; MacDonald, L. D.; Anderson, H. R.; Chamberlain, J.; Freeling, P.; Bland, J. M.; Raftery, J. | 1992 | Randomised controlled trial of effects of coordinating care for terminally ill cancer patients | British Medical Journal (BMJ) | 305 | | 6865 | 1317-22 |
| Addis, G. | 2003 | The effect of home visits after discharge on patients who have had an ileostomy or a colostomy | World Council of Enterostomal Therapists Journal | 23 | | 1 | 26-33 |
| Ahmed, O. I.; Rak, D. J. | 2010 | Hospital readmission among participants in a transitional case management program | American Journal of Managed Care | 16 | | 10 | 778-83 |
| Alem, N.; Rinehart, J.; Lee, B.; Merrill, D.; Sobhanie, S.; Ahn, K.; Schwarzkopf, R.; Cannesson, M.; Kain, Z. | 2016 | A case management report: a collaborative perioperative surgical home paradigm and the reduction of total joint arthroplasty readmissions | Perioperative Medicine | 5 | |  | 27 |
| Allen, K.; Hazelett, S.; Jarjoura, D.; Hua, K.; Wright, K.; Weinhardt, J.; Kropp, D. | 2009 | A randomized trial testing the superiority of a postdischarge care management model for stroke survivors | Journal of Stroke & Cerebrovascular Diseases | 18 | | 6 | 443-52 |
| Al-Rashed, S. A.; Wright, D. J.; Roebuck, N.; Sunter, W.; Chrystyn, H. | 2002 | The value of inpatient pharmaceutical counselling to elderly patients prior to discharge | British Journal of Clinical Pharmacology | 54 | | 6 | 657-64 |
| Anderegg, S. V.; Wilkinson, S. T.; Couldry, R. J.; Grauer, D. W.; Howser, E. | 2014 | Effects of a hospitalwide pharmacy practice model change on readmission and return to emergency department rates | American Journal of Health-System Pharmacy | 71 | | 17 | 1469-79 |
| Anderson, D.; Backaler, M. | 2008 | Hospitalization and emergent care risk assessment | Home Health Care Management & Practice | 20 | | 2 | 117-124 |
| Anderson, S. L.; Marrs, J. C.; Vande Griend, J. P.; Hanratty, R. | 2013 | Implementation of a clinical pharmacy specialist-managed telephonic hospital discharge follow-up program in a patient-centered medical home | Population Health Management | 16 | | 4 | 235-41 |
| Arbaje, A. I.; Maron, D. D.; Yu, Q.; Wendel, V. I.; Tanner, E.; Boult, C.; Eubank, K. J.; Durso, S. C. | 2010 | The geriatric floating interdisciplinary transition team | Journal of the American Geriatrics Society | 58 | | 2 | 364-70 |
| Arnold, Matthew E.; Buys, Lucinda; Fullas, Fekadu | 2015 | Impact of pharmacist intervention in conjunction with outpatient physician follow-up visits after hospital discharge on readmission rate | American Journal of Health-System Pharmacy | 72 | | S1 | S36-S42 |
| Aronovitch, S. A.; Sharp, R.; Harduar-Morano, L. | 2010 | Quality of life for patients living with ostomies: influence of contact with an ostomy nurse | Journal of Wound, Ostomy, & Continence Nursing | 37 | | 6 | 649-53 |
| Austin, L. S.; Landis, C. O.; Hanger, K. H., Jr. | 2012 | Extending the continuum of care in congestive heart failure: an interactive technology self-management solution | Journal of Nursing Administration | 42 | | 9 | 442-6 |
| Axon, R. N.; Cole, L.; Moonan, A.; Foster, R.; Cawley, P.; Long, L.; Turley, C. B. | 2016 | Evolution and Initial Experience of a Statewide Care Transitions Quality Improvement Collaborative: Preventing Avoidable Readmissions Together | Population Health Management | 19 | | 1 | 4-10 |
| Ã–zalp GerÃ§eker, GÃ¼lÃ§in; KarayaÄŸÄ±z Muslu, Gonca; Yardimci, Figen | 2016 | Children's postoperative symptoms at home through nurse-led telephone counseling and its effects on parents' anxiety: A randomized controlled trial | Journal for Specialists in Pediatric Nursing | 21 | | 4 | 189-199 |
| Badger, N.; Mullis, S.; Butler, K.; Tucker, D. | 2007 | Pharmacist's intervention for older hospitalized patients | American Journal of Health-System Pharmacy | 64 | | 17 | 1794-1796 |
| Bager, P.; Vilstrup, H. | 2010 | Post-discharge brief intervention increases the frequency of alcohol abstinence - a randomized trial | Journal of Addictions Nursing (Taylor & Francis Ltd) | 21 | | 1 | 37-41 |
| Bagnasco, A.; Calza, S.; Petralia, P.; Aleo, G.; Fornoni, L.; Sasso, L. | 2016 | Investigating the use of Barrows Cards to improve self-management and reduce healthcare costs in adolescents with blood cancer: a pilot study | Journal of Advanced Nursing | 72 | | 4 | 754-8 |
| Balaban, R. B.; Weissman, J. S.; Samuel, P. A.; Woolhandler, S. | 2008 | Redefining and redesigning hospital discharge to enhance patient care: a randomized controlled study | Journal of General Internal Medicine | 23 | | 8 | 1228-33 |
| Balling, L.; Erstad, B. L.; Weibel, K. | 2015 | Impact of a transition-of-care pharmacist during hospital discharge | Journal of the American Pharmacists Association: JAPhA | 55 | | 4 | 443-8 |
| Barker, A.; Barlis, P.; Berlowitz, D.; Page, K.; Jackson, B.; Lim, W. K. | 2012 | Pharmacist directed home medication reviews in patients with chronic heart failure: a randomised clinical trial | International Journal of Cardiology | 159 | | 2 | 139-43 |
| Barnason, S.; Zimmerman, L.; Nieveen, J.; Schmaderer, M.; Carranza, B.; Reilly, S. | 2003 | Impact of a home communication intervention for coronary artery bypass graft patients with ischemic heart failure on self-efficacy, coronary disease risk factor modification, and functioning | Heart & Lung | 32 | | 3 | 147-58 |
| Basso Lipani, M.; Holster, K.; Bussey, S. | 2015 | The Preventable Admissions Care Team (PACT): A Social Work-Led Model of Transitional Care | Social Work in Health Care | 54 | | 9 | 810-827 |
| Bates, O. L.; O'Connor, N.; Dunn, D.; Hasenau, S. M. | 2014 | Applying STAAR interventions in incremental bundles: improving post-CABG surgical patient care | Worldviews on Evidence-Based Nursing | 11 | | 2 | 89-97 |
| Beauchesne, M.; Nenciu, L. M.; Dinh, T.; TassÃ©, M.; Fillion, A.; Labrecque, M.; Blais, L. | 2007 | Active communication of a pharmacy discharge plan for patients with respiratory diseases: a pilot study | Journal of Pharmacy Technology | 23 | | 2 | 67-74 |
| Bell, S. P.; Schnipper, J. L.; Goggins, K.; Bian, A.; Shintani, A.; Roumie, C. L.; Dalal, A. K.; Jacobson, T. A.; Rask, K. J.; Vaccarino, V.; Gandhi, T. K.; Labonville, S. A.; Johnson, D.; Neal, E. B.; Kripalani, S.; Pharmacist Intervention for Low Literacy in Cardiovascular Disease Study, Group | 2016 | Effect of Pharmacist Counseling Intervention on Health Care Utilization Following Hospital Discharge: A Randomized Control Trial | Journal of General Internal Medicine | 31 | | 5 | 470-7 |
| Bellone, J. M.; Barner, J. C.; Lopez, D. A. | 2012 | Postdischarge interventions by pharmacists and impact on hospital readmission rates | Journal of the American Pharmacists Association: JAPhA | 52 | | 3 | 358-62 |
| Beney, J.; Devine, E. B.; Chow, V.; Ignoffo, R. J.; Mitsunaga, L.; Shahkarami, M.; McMillan, A.; Bero, L. A. | 2002 | Effect of telephone follow-up on the physical well-being dimension of quality of life in patients with cancer | Pharmacotherapy: The Journal of Human Pharmacology & Drug Therapy | 22 | | 10 | 1301-11 |
| Bentley, C. L.; Mountain, G. A.; Thompson, J.; Fitzsimmons, D. A.; Lowrie, K.; Parker, S. G.; Hawley, M. S. | 2014 | A pilot randomised controlled trial of a Telehealth intervention in patients with chronic obstructive pulmonary disease: challenges of clinician-led data collection | Trials | 15 | |  | 313 |
| Berkman, P.; Heinik, J.; Rosenthal, M.; Burke, M. | 1999 | Supportive telephone outreach as an interventional strategy for elderly patients in a period of crisis | Social Work in Health Care | 28 | | 4 | 63-76 |
| Berkowitz, R. E.; Fang, Z.; Helfand, B. K.; Jones, R. N.; Schreiber, R.; Paasche-Orlow, M. K. | 2013 | Project ReEngineered Discharge (RED) lowers hospital readmissions of patients discharged from a skilled nursing facility | Journal of the American Medical Directors Association | 14 | | 10 | 736-40 |
| Bisharat, B.; Hafi, L.; Baron-Epel, O.; Armaly, Z.; Bowirrat, A. | 2012 | Pharmacist counseling to cardiac patients in Israel prior to discharge from hospital contribute to increasing patient's medication adherence closing gaps and improving outcomes | Journal of Translational Medicine | 10 | |  | 34 |
| Blue, L.; Lang, E.; McMurray, J. J.; Davie, A. P.; McDonagh, T. A.; Murdoch, D. R.; Petrie, M. C.; Connolly, E.; Norrie, J.; Round, C. E.; Ford, I.; Morrison, C. E. | 2001 | Randomised controlled trial of specialist nurse intervention in heart failure | British Medicial Journal (BMJ) | 323 | | 7315 | 715-18 |
| Booth, K. A.; Vinci, L. M.; Oyler, J. L.; Pincavage, A. T. | 2014 | Using a resident discharge clinic for resident education and patient care: a feasibility study | Journal of Graduate Medical Education | 6 | | 3 | 536-40 |
| Boter, H.; Mistiaen, P.; Groenewegen, I. | 2000 | A randomized trial of a Telephone Reassurance Programme for patients recently discharged from an ophthalmic unit | Journal of Clinical Nursing | 9 | | 2 | 199-206 |
| Bowman, G. S.; Howden, J.; Allen, S.; Webster, R. A.; Thompson, D. R. | 1994 | A telephone survey of medical patients 1 week after discharge from hospital | Journal of Clinical Nursing | 3 | | 6 | 369-73 |
| Bradburn, M.; Goodacre, S. W.; Fitzgerald, P.; Coats, T.; Gray, A.; Hassan, T.; Humphrey, J.; Kendall, J.; Smith, J.; Collinson, P.; Ratpac Research Team | 2012 | Interhospital variation in the RATPAC trial (Randomised Assessment of Treatment using Panel Assay of Cardiac markers) | Emergency Medicine Journal | 29 | | 3 | 233-8 |
| Bradley, E. H.; Sipsma, H.; Horwitz, L. I.; Ndumele, C. D.; Brewster, A. L.; Curry, L. A.; Krumholz, H. M. | 2015 | Hospital strategy uptake and reductions in unplanned readmission rates for patients with heart failure: a prospective study | Journal of General Internal Medicine | 30 | | 5 | 605-11 |
| Bradywood, A.; Farrokhi, F.; Williams, B.; Kowalczyk, M.; Blackmore, C. C. | 2016 | Reduction of Inpatient Hospital Length of Stay in Lumbar Fusion Patients with Implementation of an Evidence Based Clinical Care Pathway | Spine | 47 | | 3 | 169-76 |
| Bretz, M. N.; Graves, A.; West, A.; Kiesz, K. C.; Toth, L.; Welch, M. | 2014 | Steps against recurrent stroke plus: patient transition program | Journal of Neuroscience Nursing | 46 | | 4 | E3-13; quiz E1-2 |
| Brock, J.; Mitchell, J.; Irby, K.; Stevens, B.; Archibald, T.; Goroski, A.; Lynn, J.; Care Transitions Project, Team | 2013 | Association between quality improvement for care transitions in communities and rehospitalizations among Medicare beneficiaries | JAMA | 309 | | 4 | 381-91 |
| Brown, D. S. | 1995 | Hospital discharge preparation for homeward bound elderly | Clinical Nursing Research | 4 | | 2 | 181-94 |
| Brown, Randall S.; Peikes, Deborah; Peterson, Greg; Schore, Jennifer; Razafundrakoto, Carol M. | 2012 | Six Features Of Medicare Coordinated Care Demonstration Programs That Cut Hospital Admissions Of High-Risk Patients | Health Affairs | 31 | | 6 | 1156-1166 |
| Bruce Bayley, K.; Savitz, L. A.; Maddalone, T.; Stoner, S. E.; Hunt, J. S.; Wells, R. | 2007 | Evaluation of patient care interventions and recommendations by a transitional care pharmacist | Therapeutics & Clinical Risk Management | 3 | | 4 | 695-703 |
| Brumm, S.; Theisen, K.; Falciglia, M. | 2016 | Diabetes Transition Care From an Inpatient to Outpatient Setting in a Veteran Population: Quality Improvement Pilot Study | Diabetes Educator | 42 | | 3 | 346-53 |
| Budiman, T.; Snodgrass, K.; Komatsu Chang, A. | 2016 | Evaluation of Pharmacist Medication Education and Post-discharge Follow-up in Reducing Readmissions in Patients With ST-Segment Elevation Myocardial Infarction (STEMI) | Annals of Pharmacotherapy | 50 | | 2 | 118-24 |
| Bumpus, Sherry M. | 2012 | Bridging the Discharge Gap Effectively (BRIDGE): A Novel Approach to Transitional Care for Patients with Acute Coronary Syndrome | ProQuest Dissertation Publishing | Ph.D. | |  |  |
| Burke, R. E.; Whitfield, E.; Prochazka, A. V. | 2014 | Effect of a hospitalist-run postdischarge clinic on outcomes | Journal of Hospital Medicine | 9 | | 1 | 7-12 |
| Burkey, S. L. | 1979 | An audit outcome: home going instructions | Supervisor Nurse | 10 | | 5 | 36-43 |
| Bushnell, C.; Arnan, M.; Han, S. | 2014 | A new model for secondary prevention of stroke: transition coaching for stroke | Frontiers in Neurology | 5 | |  | 219 |
| Buurman, B. M.; Verhaegh, K. J.; Smeulers, M.; Vermeulen, H.; Geerlings, S. E.; Smorenburg, S.; de Rooij, S. E. | 2016 | Improving handoff communication from hospital to home: the development, implementation and evaluation of a personalized patient discharge letter | International Journal for Quality in Health Care | 28 | | 3 | 384-90 |
| Buurman, B. M.; Parlevliet, J. L.; Allore, H. G.; Blok, W.; van Deelen, B. A.; Moll van Charante, E. P.; de Haan, R. J.; de Rooij, S. E. | 2016 | Comprehensive Geriatric Assessment and Transitional Care in Acutely Hospitalized Patients: The Transitional Care Bridge Randomized Clinical Trial | JAMA Internal Medicine | 176 | | 3 | 302-9 |
| Calugi, S.; Taricco, M.; Rucci, P.; Fugazzaro, S.; Stuart, M.; Dallolio, L.; Pillastrini, P.; Fantini, M. P.; E. F. G. investigators | 2016 | Effectiveness of adaptive physical activity combined with therapeutic patient education in stroke survivors at twelve months: a non-randomized parallel group study | European journal of physical & rehabilitation medicine. | 52 | | 1 | 72-80 |
| Cam, Rahsan; Korkmaz, Fatma Demir | 2014 | The effect of long-term care and follow-up on complications in patients with external fixators | International Journal of Nursing Practice | 20 | | 1 | 89-96 |
| Cardozo, L.; Steinberg, J. | 2010 | Telemedicine for recently discharged older patients | Telemedicine Journal & E-Health | 16 | | 1 | 49-55 |
| Carney, N. A.; Petroni, G. J.; Lujan, S. B.; Ballarini, N. M.; Faguaga, G. A.; du Coudray, H. E.; Huddleston, A. E.; Baggio, G. M.; Becerra, J. M.; Busso, L. O.; Dikmen, S. S.; Falcone, R.; Garcia, M. E.; Gonzalez Carrillo, O. R.; Medici, P. L.; Quaglino, M. B.; Randisi, C. A.; Saenz, S. S.; Temkin, N. R.; Vanella, E. E. | 2016 | Postdischarge Care of Pediatric Traumatic Brain Injury in Argentina: A Multicenter Randomized Controlled Trial | Pediatric Critical Care Medicine | 17 | | 7 | 658-66 |
| Carter, B. L.; Farris, K. B.; Abramowitz, P. W.; Weetman, D. B.; Kaboli, P. J.; Dawson, J. D.; James, P. A.; Christensen, A. J.; Brooks, J. M. | 2008 | The Iowa Continuity of Care study: Background and methods | American Journal of Health-System Pharmacy | 65 | | 17 | 1631-42 |
| Carter, J. A.; Carr, L. S.; Collins, J.; Doyle Petrongolo, J.; Hall, K.; Murray, J.; Smith, J.; Tata, L. A. | 2015 | STAAR: improving the reliability of care coordination and reducing hospital readmissions in an academic medical centre | BMJ Innovations | 1 | | 3 | 75-80 |
| Cawthon, C.; Walia, S.; Osborn, C. Y.; Niesner, K. J.; Schnipper, J. L.; Kripalani, S. | 2012 | Improving care transitions: the patient perspective | Journal of Health Communication | 17 | | Suppl 3 | 312-24 |
| Cebeci, F.; Celik, S. S. | 2008 | Discharge training and counselling increase self-care ability and reduce postdischarge problems in CABG patients | Journal of Clinical Nursing | 17 | | 3 | 412-20 |
| Chaiyawat, P.; Kulkantrakorn, K. | 2012 | Randomized controlled trial of home rehabilitation for patients with ischemic stroke: impact upon disability and elderly depression | Psychogeriatrics:The Official Journal of the Japanese Psychogeriatric Society | 12 | | 3 | 193-9 |
| Chan, C.; Tang, D.; Jones, A. | 2008 | Clinical outcomes of a Cardiac Rehabilitation and Maintenance Program for Chinese patients with congestive heart failure | Disability & Rehabilitation | 30 | | 17 | 1245-53 |
| Chang, R.; Spahlinger, D.; Kim, C. S. | 2012 | Re-engineering the post-discharge appointment process for general medicine patients | The Patient: Patient-Centered Outcomes Research | 5 | | 1 | 27-32 |
| Chen, H. Y.; Wu, T. J.; Lin, C. C. | 2015 | Improving self-perception and self-efficacy in patients with spinal cord injury: the efficacy of DVD-based instructions | Journal of Clinical Nursing | 24 | | 11-12 | 1666-75 |
| Cheng, Q.; Church, J.; Haas, M.; Goodall, S.; Sangster, J.; Furber, S. | 2016 | Cost-effectiveness of a Population-based Lifestyle Intervention to Promote Healthy Weight and Physical Activity in Non-attenders of Cardiac Rehabilitation | Heart, Lung & Circulation | 25 | | 3 | 265-74 |
| Christy, S.; Sin, B.; Gim, S. | 2016 | Impact of an Integrated Pharmacy Transitions of Care Pilot Program in an Urban Hospital | Journal of Pharmacy Practice | 29 | | 5 | 490-4 |
| Clarke, R.; Bharmal, N.; Di Capua, P.; Tseng, C. H.; Mangione, C. M.; Mittman, B.; Skootsky, S. A. | 2015 | Innovative approach to patient-centered care coordination in primary care practices | American Journal of Managed Care | 21 | | 9 | 623-30 |
| Clarkson, J. N.; Schaffer, S. D.; Clarkson, J. J. | 2017 | The Effect of an Interprofessional Heart Failure Education Program on Hospital Readmissions | Journal for Healthcare Quality | 39 | | 2 | 78-84 |
| Clemson, L.; Lannin, N. A.; Wales, K.; Salkeld, G.; Rubenstein, L.; Gitlin, L.; Barris, S.; Mackenzie, L.; Cameron, I. D. | 2016 | Occupational Therapy Predischarge Home Visits in Acute Hospital Care: A Randomized Trial | Journal of the American Geriatrics Society | 64 | | 10 | 2019-2026 |
| Colandrea, M.; Eckardt, P. | 2016 | Improving Tracheostomy Care Delivery: Instituting Clinical Care Pathways and Nursing Education to Improve Patient Outcomes | ORL - Head & Neck Nursing | 34 | | 1 | 7-16 |
| Coleman, E. A.; Parry, C.; Chalmers, S. A.; Chugh, A.; Mahoney, E. | 2007 | The central role of performance measurement in improving the quality of transitional care | Home Health Care Services Quarterly | 26 | | 4 | 93-104 |
| Condon, C.; Lycan, S.; Duncan, P.; Bushnell, C. | 2016 | Reducing Readmissions After Stroke With a Structured Nurse Practitioner/Registered Nurse Transitional Stroke Program | Stroke | 47 | | 6 | 1599-604 |
| Connolly, M. L.; Russell, M.; Reyna, C. | 1995 | Home care of the prostate cryosurgery patient | Todays Or-Nurse | 17 | | 3 | 25-36 |
| Costa, L. L.; Poe, S. S.; Lee, M. C. | 2011 | Challenges in posthospital care: nurses as coaches for medication management | Journal of Nursing Care Quality | 26 | | 3 | 243-51 |
| Costantino, M. E.; Frey, B.; Hall, B.; Painter, P. | 2013 | The influence of a postdischarge intervention on reducing hospital readmissions in a Medicare population | Population Health Management | 16 | | 5 | 310-6 |
| Creason, H. | 2001 | Congestive heart failure telemanagement clinic | Lippincott's Case Management | 6 | | 4 | 146-56 |
| Crennan, M.; MacRae, A. | 2010 | Occupational therapy discharge assessment of elderly patients from acute care hospitals | Physical & Occupational Therapy in Geriatrics | 28 | | 1 | 33-43 |
| Crossen-Sills, J.; Toomey, I.; Doherty, M. | 2006 | Strategies to reduce unplanned hospitalizations of home healthcare patients: a step-by-step approach | Home Healthcare Nurse | 24 | | 6 | 368-376 |
| Crotty, M.; Whitehead, C. H.; Gray, S.; Finucane, P. M. | 2002 | Early discharge and home rehabilitation after hip fracture achieves functional improvements: a randomized controlled trial | Clinical Rehabilitation | 16 | | 4 | 406-13 |
| Crotty, M.; Whitehead, C.; Miller, M.; Gray, S. | 2003 | Patient and caregiver outcomes 12 months after home-based therapy for hip fracture: a randomized controlled trial | Archives of Physical Medicine & Rehabilitation | 84 | | 8 | 1237-9 |
| Crotty, M.; Whitehead, C. H.; Wundke, R.; Giles, L. C.; Ben-Tovim, D.; Phillips, P. A. | 2005 | Transitional care facility for elderly people in hospital awaiting a long term care bed: randomised controlled trial | British Medical Journal (BMJ) | 331 | | 7525 | 1110 |
| Cucinotta, D.; Savorani, G.; Piscaglia, F.; Galletti, L.; Petazzoni, E.; Bolondi, L. | 2004 | The chronically ill elderly patients discharged from the hospital: interim report from a controlled study of home care attendance | Archives of Gerontology & Geriatrics - Supplement | 38 | |  | 103-8 |
| Cuffel, B. J.; Held, M.; Goldman, W. | 2002 | Predictive models and the effectiveness of strategies for improving outpatient follow-up under managed care | Psychiatric Services | 53 | | 11 | 1438-43 |
| Cunliffe, A. L.; Gladman, J. R.; Husbands, S. L.; Miller, P.; Dewey, M. E.; Harwood, R. H. | 2004 | Sooner and healthier: a randomised controlled trial and interview study of an early discharge rehabilitation service for older people | Age & Ageing | 33 | | 3 | 246-52 |
| Dallolio, L.; Menarini, M.; China, S.; Ventura, M.; Stainthorpe, A.; Soopramanien, A.; Rucci, P.; Fantini, M. P.; Thrive Project | 2008 | Functional and clinical outcomes of telemedicine in patients with spinal cord injury | Archives of Physical Medicine & Rehabilitation | 89 | | 12 | 2332-41 |
| Damiani, G.; Federico, B.; Venditti, A.; Sicuro, L.; Rinaldi, S.; Cirio, F.; Pregno, C.; Ricciardi, W. | 2009 | Hospital discharge planning and continuity of care for aged people in an Italian local health unit: does the care-home model reduce hospital readmission and mortality rates? | BMC Health Services Research | 9 | | 1 | 22 |
| Dash, I.; Pickering, G. T. | 2017 | Improving post-operative communication between primary and secondary care: the wound closure information card | Primary Health Care Research & Development | 18 | | 1 | 92-96 |
| DeBusk, R. F.; Miller, N. H.; Parker, K. M.; Bandura, A.; Kraemer, H. C.; Cher, D. J.; West, J. A.; Fowler, M. B.; Greenwald, G. | 2004 | Improving patient care. Care management for low-risk patients with heart failure: a randomized, controlled trial | Annals of Internal Medicine | 141 | | 8 | 606-I58 |
| Dedhia, P.; Kravet, S.; Bulger, J.; Hinson, T.; Sridharan, A.; Kolodner, K.; Wright, S.; Howell, E. | 2009 | A quality improvement intervention to facilitate the transition of older adults from three hospitals back to their homes | Journal of the American Geriatrics Society | 57 | | 9 | 1540-6 |
| Delaney, Colleen; Apostolidis, Beka; Bartos, Susan; Morrison, Heather; Smith, Liane; Fortinsky, Richard | 2013 | A Randomized Trial of Telemonitoring and Self-Care Education in Heart Failure Patients Following Home Care Discharge | Home Health Care Management & Practice | 25 | | 5 | 187-195 |
| de Lapasse, C.; Rabischong, B.; Bolandard, F.; Canis, M.; Botchorischvili, R.; Jardon, K.; Mage, G. | 2008 | Total laparoscopic hysterectomy and early discharge: satisfaction and feasibility study | Journal of Minimally Invasive Gynecology | 15 | | 1 | 20-25 |
| Delate, T.; Chester, E. A.; Stubbings, T. W.; Barnes, C. A. | 2008 | Clinical outcomes of a home-based medication reconciliation program after discharge from a skilled nursing facility | Pharmacotherapy:The Journal of Human Pharmacology & Drug Therapy | 28 | | 4 | 444-52 |
| de Wit, R.; van Dam, F. | 2001 | From hospital to home care: a randomized controlled trial of a Pain Education Programme for cancer patients with chronic pain | Journal of Advanced Nursing | 36 | | 6 | 742-54 |
| Dietrick-Gallagher, M.; Hyzinski, M. M. | 1989 | Teaching patients to care for drains after breast surgery for malignancy | Oncology Nursing Forum | 16 | | 2 | 263-5 |
| Dudas, V.; Bookwalter, T.; Kerr, K. M.; Pantilat, S. Z. | 2001 | The impact of follow-up telephone calls to patients after hospitalization | American Journal of Medicine | 111 | | 9B | 26S-30S |
| Dudas, V.; Bookwalter, T.; Kerr, K. M.; Pantilat, S. Z. | 2002 | The impact of follow-up telephone calls to patients after hospitalization | Disease-A-Month | 48 | | 4 | 239-48 |
| Edmans, J.; Bradshaw, L.; Franklin, M.; Gladman, J.; Conroy, S. | 2013 | Specialist geriatric medical assessment for patients discharged from hospital acute assessment units: randomised controlled trial | British Medical Journal (BMJ) | 347 | |  | f5874 |
| Einstadter, D.; Cebul, R. D.; Franta, P. R. | 1996 | Effect of a nurse case manager on postdischarge follow-up | Journal of General Internal Medicine | 11 | | 11 | 684-8 |
| Ekim, A.; Ocakci, A. F. | 2016 | Efficacy of a Transition Theory-Based Discharge Planning Program for Childhood Asthma Management | Pediatric Obesity | 27 | | 2 | 70-78 |
| Ellis, R. F.; Stephens, M. A.; Sharp, G. B. | 1992 | Evaluation of a pharmacy-managed warfarin-monitoring service to coordinate inpatient and outpatient therapy | American Journal of Hospital Pharmacy | 49 | | 2 | 387-94 |
| Elson, R.; Cook, H.; Blenkinsopp, A. | 2016 | Patients' knowledge of new medicines after discharge from hospital: What are the effects of hospital-based discharge counseling and community-based medicines use reviews (MURs)? | Research In Social & Administrative Pharmacy | 13 | | 3 | 628-633 |
| Emery, D.; Pearson, A.; Lopez, R.; Hamilton, C.; Albert, N. M. | 2015 | Voiceover Interactive PowerPoint Catheter Care Education for Home Parenteral Nutrition | Nutrition in Clinical Practice | 30 | | 5 | 714-9 |
| Englander, H.; Michaels, L.; Chan, B.; Kansagara, D. | 2014 | The care transitions innovation (C-TraIn) for socioeconomically disadvantaged adults: results of a cluster randomized controlled trial | Journal of General Internal Medicine | 29 | | 11 | 1460-7 |
| Enguidanos, S.; Gibbs, N.; Jamison, P. | 2012 | From hospital to home: a brief nurse practitioner intervention for vulnerable older adults | Journal of Gerontological Nursing | 38 | | 3 | 40-50 |
| Esposito, L. | 1995 | The effects of medication education on adherence to medication regimens in an elderly population | Journal of Advanced Nursing | 21 | | 5 | 935-43 |
| Evangelista, L. S.; Lee, J. A.; Moore, A. A.; Motie, M.; Ghasemzadeh, H.; Sarrafzadeh, M.; Mangione, C. M. | 2015 | Examining the effects of remote monitoring systems on activation, self-care, and quality of life in older patients with chronic heart failure | Journal of Cardiovascular Nursing | 30 | | 1 | 51-57 |
| Fagermoen, M. S.; Hamilton, G. | 2006 | Patient information at discharge-a study of a combined approach | Patient Education & Counseling | 63 | | 1 | 169-76 |
| Farley, T. M.; Shelsky, C.; Powell, S.; Farris, K. B.; Carter, B. L. | 2014 | Effect of clinical pharmacist intervention on medication discrepancies following hospital discharge | International Journal of Clinical Pharmacy | 36 | | 2 | 430-7 |
| Farrell, T. W.; Tomoaia-Cotisel, A.; Scammon, D. L.; Brunisholz, K.; Kim, J.; Day, J.; Gren, L. H.; Wallace, S.; Gunning, K.; Tabler, J.; Magill, M. K. | 2015 | Impact of an integrated transition management program in primary care on hospital readmissions | Journal for Healthcare Quality | 37 | | 1 | 81-92 |
| Farris, K. B.; Carter, B. L.; Xu, Y.; Dawson, J. D.; Shelsky, C.; Weetman, D. B.; Kaboli, P. J.; James, P. A.; Christensen, A. J.; Brooks, J. M. | 2014 | Effect of a care transition intervention by pharmacists: an RCT | BMC Health Services Research | 14 | | 1 | 406 |
| Felsenthal, G.; Glomski, N.; Jones, D. | 1986 | Medication education program in an inpatient geriatric rehabilitation unit | Archives of Physical Medicine & Rehabilitation | 67 | | 1 | 9-27 |
| Fine, Michelle; Mutharasan, R. Kannan; Kansal, Preeti; Jackson, Hannah Alphs; Benacka, Corrine; Vlcek, Amanda; Fortman, Robin; Davidson, Charles; Anderson, Allen S.; Yancy, Clyde W. | 2016 | Pharmacist-Led Transition of Care Interventions Identify and Reduce Medication Errors in Heart Failure Patients Post-Discharge | Journal of Cardiac Failure | 22 | | 8 | S134-S135 |
| Finn, K. M.; Heffner, R.; Chang, Y.; Bazari, H.; Hunt, D.; Pickell, K.; Berube, R.; Raju, S.; Farrell, E.; Iyasere, C.; Thompson, R.; O'Malley, T.; O'Donnell, W.; Karson, A. | 2011 | Improving the discharge process by embedding a discharge facilitator in a resident team | Journal of Hospital Medicine | 6 | | 9 | 494-500 |
| Forster, A.; Young, J.; Nixon, J.; Kalra, L.; Smithard, D.; Patel, A.; Knapp, M.; Monaghan, J.; Breen, R.; Anwar, S.; Farrin, A. | 2012 | A cluster randomized controlled trial of a structured training programme for caregivers of inpatients after stroke (TRACS) | International Journal of Stroke | 7 | | 1 | 94-99 |
| Forster, A.; Dickerson, J.; Young, J.; Patel, A.; Kalra, L.; Nixon, J.; Smithard, D.; Knapp, M.; Holloway, I.; Anwar, S.; Farrin, A.; Tracs Trial Collaboration | 2013 | A cluster randomised controlled trial and economic evaluation of a structured training programme for caregivers of inpatients after stroke: the TRACS trial | Health Technology Assessment | 17 | | 46 | 1-216 |
| Foust, J. B.; Naylor, M. D.; Bixby, M. B.; Ratcliffe, S. J. | 2012 | Medication problems occurring at hospital discharge among older adults with heart failure | Research in Gerontological Nursing | 5 | | 1 | 25-33 |
| Frail, C. K.; Garza, O. W.; Haas, A. L. | 2016 | Experience with technology-supported transitions of care to improve medication use | Journal of the American Pharmacists Association: JAPhA | 56 | | 5 | 568-72 |
| Gardella, J. E.; Cardwell, T. B.; Nnadi, M. | 2012 | Improving medication safety with accurate preadmission medication lists and postdischarge education | Joint Commission Journal on Quality & Patient Safety | 38 | | 10 | 452-8 |
| Gardner, R.; Li, Q.; Baier, R. R.; Butterfield, K.; Coleman, E. A.; Gravenstein, S. | 2014 | Is implementation of the care transitions intervention associated with cost avoidance after hospital discharge? | Journal of General Internal Medicine | 29 | | 6 | 878-84 |
| Gilbertson, L.; Langhorne, P.; Walker, A.; Allen, A.; Murray, G. D. | 2000 | Domiciliary occupational therapy for patients with stroke discharged from hospital: randomised controlled trial | British Medical Journal (BMJ) | 320 | | 7235 | 603-6 |
| Gould, K. A. | 2009 | A randomized controlled trial of a discharge nursing intervention to promote self-regulation of care for early discharge interventional cardiology patients | ProQuest Dissertations Publishing | Ph.D. | |  |  |
| Gow, P.; Berg, S.; Smith, D.; Ross, D. | 1999 | Care co-ordination improves quality-of-care at South Auckland Health | Journal of Quality in Clinical Practice | 19 | | 2 | 107-110 |
| Green, Uthona R.; Dearmon, Valorie; Taggart, Helen | 2015 | Improving Transition of Care for Veterans After Total Joint Replacement | Orthopaedic Nursing | 34 | | 2 | 79-88 |
| Gregersen, M.; Morch, M. M.; Hougaard, K.; Damsgaard, E. M. | 2012 | Geriatric intervention in elderly patients with hip fracture in an orthopedic ward | Journal of Injury & Violence Research | 4 | | 2 | 45-51 |
| Griffin, A.; Skinner, A.; Thornhill, J.; Weinberger, M. | 2016 | Patient Portals: Who uses them? What features do they use? And do they reduce hospital readmissions? | Applied Clinical Informatics | 7 | | 2 | 489-501 |
| Grimmer, K. A.; Dryden, L. R.; Puntumetakul, R.; Young, A. F.; Guerin, M.; Deenadayalan, Y.; Moss, J. R. | 2006 | Incorporating patient concerns into discharge plans: evaluation of a patient-generated checklist | Internet Journal of Allied Health Sciences & Practice | 4 | | 2 | 1-23 |
| Gwadry-Sridhar, F. H.; Arnold, J. M.; Zhang, Y.; Brown, J. E.; Marchiori, G.; Guyatt, G. | 2005 | Pilot study to determine the impact of a multidisciplinary educational intervention in patients hospitalized with heart failure | American Heart Journal | 150 | | 5 | 982 |
| Haag, J. D.; Davis, A. Z.; Hoel, R. W.; Armon, J. J.; Odell, L. J.; Dierkhising, R. A.; Takahashi, P. Y. | 2016 | Impact of Pharmacist-Provided Medication Therapy Management on Healthcare Quality and Utilization in Recently Discharged Elderly Patients | American Health & Drug Benefits | 9 | | 5 | 259-68 |
| Haddock, K. S. | 1994 | Collaborative discharge planning: nursing and social services | Clinical Nurse Specialist | 8 | | 5 | 248-52, 288 |
| Haggmark, C.; Nilsson, B. | 1997 | Effects of an intervention programme for improved discharge-planning | Nordic Journal of Nursing Research & Clinical Studies / VÃ¥rd i Norden | 17 | | 2 | 4-8 |
| Hansen, F. R.; Poulsen, H.; Sorensen, K. H. | 1995 | A model of regular geriatric follow-up by home visits to selected patients discharged from a geriatric ward: a randomized controlled trial | Aging-Clinical & Experimental Research | 7 | | 3 | 202-6 |
| Hansen, L. O.; Greenwald, J. L.; Budnitz, T.; Howell, E.; Halasyamani, L.; Maynard, G.; Vidyarthi, A.; Coleman, E. A.; Williams, M. V. | 2013 | Project BOOST: effectiveness of a multihospital effort to reduce rehospitalization | Journal of Hospital Medicine | 8 | | 8 | 421-7 |
| Harahsheh, A. S.; Hom, L. A.; Clauss, S. B.; Cross, R. R.; Curtis, A. R.; Steury, R. D.; Mitchell, S. J.; Martin, G. R. | 2016 | The Impact of a Designated Cardiology Team Involving Telemedicine Home Monitoring on the Care of Children with Single-Ventricle Physiology After Norwood Palliation | Pediatric Cardiology | 37 | | 5 | 899-912 |
| Hardiman, K. M.; Reames, C. D.; McLeod, M. C.; Regenbogen, S. E. | 2016 | Patient autonomy-centered self-care checklist reduces hospital readmissions after ileostomy creation | Surgery | 160 | | 5 | 1302-08 |
| Harrison, J. D.; Young, J. M.; Solomon, M. J.; Butow, P. N.; Secomb, R.; Masya, L. | 2011 | Randomized pilot evaluation of the supportive care intervention "CONNECT" for people following surgery for colorectal cancer | Diseases of the Colon & Rectum | 54 | | 5 | 622-31 |
| Harrison, J. D.; Auerbach, A. D.; Quinn, K.; Kynoch, E.; Mourad, M. | 2014 | Assessing the impact of nurse post-discharge telephone calls on 30-day hospital readmission rates | Journal of General Internal Medicine | 29 | | 11 | 1519-25 |
| Hendrix, Cristina; Tepfer, Sara; Forest, Sabrina; Ziegler, Karen; Fox, Valerie; Stein, Jeannette; McConnell, Eleanor S.; Hastings, Susan Nicole; Schmader, Kenneth; Colon-Emeric, Cathleen | 2013 | Transitional Care Partners: A hospital-to-home support for older adults and their caregivers | Journal of the American Association of Nurse Practitioners | 25 | | 8 | 407-14 |
| Hill, B.; Perri-Moore, S.; Kuang, J.; Bray, B. E.; Ngo, L.; Doig, A.; Zeng-Treitler, Q. | 2016 | Automated pictographic illustration of discharge instructions with Glyph: impact on patient recall and satisfaction | Journal of the American Medical Informatics Association | 23 | | 6 | 1136-42 |
| Hitch, B.; Parlier, A. B.; Reed, L.; Galvin, S. L.; Fagan, E. B.; Wilson, C. G. | 2016 | Evaluation of a Team-Based, Transition-of-Care Management Service on 30-Day Readmission Rates | North Carolina Medical Journal | 77 | | 2 | 87-92 |
| Holland, D. E.; Hemann, M. A. | 2011 | Standardizing hospital discharge planning at the Mayo Clinic | Joint Commission Journal on Quality & Patient Safety | 37 | | 1 | 29-36 |
| Hollier, L. H.; Smith, F. I.; Rice, J. C.; Kliger, C. H.; Kerstein, M. D. | 1990 | Efficacy of home health care in patients with peripheral vascular disease | American Journal of Surgery | 160 | | 2 | 179-81 |
| Huntington, M. K.; Guzman, A. I.; Roemen, A.; Fieldsend, J.; Saloum, H. | 2013 | Hospital-to-Home: a hospital readmission reduction program for congestive heart failure | South Dakota Medicine: The Journal of the South Dakota State Medical Association | 66 | | 9 | 370-73 |
| Hutchison, L. J.; Mayzell, G. G.; Bailey, S. C.; Broyles, J. E. | 2014 | Impact of a discharge medication therapy management program in an extended care hospital | Consultant Pharmacist | 29 | | 1 | 33-38 |
| Inouye, S.; Bouras, V.; Shouldis, E.; Johnstone, A.; Silverzweig, Z.; Kosuri, P. | 2015 | Predicting readmission of heart failure patients using automated follow-up calls | BMC Medical Informatics & Decision Making | 15 | |  | 22 |
| Jack, B. W.; Chetty, V. K.; Anthony, D.; Greenwald, J. L.; Sanchez, G. M.; Johnson, A. E.; Forsythe, S. R.; O'Donnell, J. K.; Paasche-Orlow, M. K.; Manasseh, C.; Martin, S.; Culpepper, L. | 2009 | A reengineered hospital discharge program to decrease rehospitalization: a randomized trial | Annals of Internal Medicine | 150 | | 3 | 178-87 |
| Jackson, C. T.; Trygstad, T. K.; DeWalt, D. A.; DuBard, C. A. | 2013 | Transitional care cut hospital readmissions for North Carolina Medicaid patients with complex chronic conditions | Health Affairs | 32 | | 8 | 1407-15 |
| Jacobs, Barbara | 2011 | Reducing heart failure hospital readmissions from skilled nursing facilities | Professional Case Management | 16 | | 1 | 18-26 |
| Jenkins, H. M.; Blank, V.; Miller, K.; Turner, J.; Stanwick, R. S. | 1996 | A randomized single-blind evaluation of a discharge teaching book for pediatric patients with burns | Journal of Burn Care & Rehabilitation | 17 | | 1 | 49-61 |
| Johnson, K. | 2000 | Use of telephone follow-up for post-cardiac surgery patients | Intensive & Critical Care Nursing | 16 | | 3 | 144-50 |
| Jorstad, H. T.; von Birgelen, C.; Alings, A. M.; Liem, A.; van Dantzig, J. M.; Jaarsma, W.; Lok, D. J.; Kragten, H. J.; de Vries, K.; de Milliano, P. A.; Withagen, A. J.; Scholte Op Reimer, W. J.; Tijssen, J. G.; Peters, R. J. | 2013 | Effect of a nurse-coordinated prevention programme on cardiovascular risk after an acute coronary syndrome: main results of the RESPONSE randomised trial | Heart | 99 | | 19 | 1421-30 |
| Joubert, J.; Reid, C.; Joubert, L.; Barton, D.; Ruth, D.; Jackson, D.; Sullivan, J. O.; Davis, S. M. | 2006 | Risk factor management and depression post-stroke: the value of an integrated model of care | Journal of Clinical Neuroscience | 13 | | 1 | 84-90 |
| Kalista, T.; Lemay, V.; Cohen, L. | 2015 | Postdischarge community pharmacist-provided home services for patients after hospitalization for heart failure | Journal of the American Pharmacists Association: JAPhA | 55 | | 4 | 438-42 |
| Kirkham, Heather S.; Clark, Bobby L.; Paynter, Jacquelyn; Lewis, Geraint H.; Duncan, Ian | 2014 | The effect of a collaborative pharmacist-hospital care transition program on the likelihood of 30-day readmission | American Journal of Health-System Pharmacy | 71 | | 9 | 739-45 |
| Kirsch, S. D.; Wilson, L. S.; Harkins, M.; Albin, D.; Del Beccaro, M. A. | 2015 | Feasibility of using a pediatric call center as part of a quality improvement effort to prevent hospital readmission | Journal of Pediatric Nursing | 30 | | 2 | 333-7 |
| Koelling, T. M.; Johnson, M. L.; Cody, R. J.; Aaronson, K. D. | 2005 | Discharge education improves clinical outcomes in patients with chronic heart failure | Circulation | 111 | | 2 | 179-85 |
| Kramer, J. S.; Hopkins, P. J.; Rosendale, J. C.; Garrelts, J. C.; Hale, L. S.; Nester, T. M.; Cochran, P.; Eidem, L. A.; Haneke, R. D. | 2007 | Implementation of an electronic system for medication reconciliation | American Journal of Health-System Pharmacy | 64 | | 4 | 404-22 |
| Kwok, T.; Lee, J.; Woo, J.; Lee, D. T. F.; Griffith, S. | 2008 | A randomized controlled trial of a community nurse-supported hospital discharge programme in older patients with chronic heart failure | Journal of Clinical Nursing | 17 | | 1 | 109-17 |
| Lambert-Kerzner, A.; Del Giacco, E. J.; Fahdi, I. E.; Bryson, C. L.; Melnyk, S. D.; Bosworth, H. B.; Davis, R.; Mun, H.; Weaver, J.; Barnett, C.; Radcliff, T.; Hubbard, A.; Bosket, K. D.; Carey, E.; Virchow, A.; Mihalko-Corbitt, R.; Kaufman, A.; Marchant-Miros, K.; Ho, P. M.; Multifaceted Intervention to Improve Cardiac Medication, Adherence; Secondary Prevention Measures Study, Investigators | 2012 | Patient-centered adherence intervention after acute coronary syndrome hospitalization | Circulation. Cardiovascular Quality & Outcomes | 5 | | 4 | 571-76 |
| Landefeld, C. S.; Palmer, R. M.; Kresevic, D. M.; Fortinsky, R. H.; Kowal, J. | 1995 | A randomized trial of care in a hospital medical unit especially designed to improve the functional outcomes of acutely ill older patients | New England Journal of Medicine | 332 | | 20 | 1338-44 |
| Lawlor, M.; Kealy, S.; Agnew, M.; Korn, B.; Quinn, J.; Cassidy, C.; Silke, B.; O'Connell, F.; O'Donnell, R. | 2009 | Early discharge care with ongoing follow-up support may reduce hospital readmissions in COPD | International Journal of Copd | 4 | |  | 55-60 |
| Linne, A. B.; Liedholm, H. | 2006 | Effects of an interactive CD-program on 6 months readmission rate in patients with heart failure - a randomised, controlled trial [NCT00311194] | BMC Cardiovascular Disorders | 6 | |  | 30 |
| Luder, H. R.; Frede, S. M.; Kirby, J. A.; Epplen, K.; Cavanaugh, T.; Martin-Boone, J. E.; Conrad, W. F.; Kuhlmann, D.; Heaton, P. C. | 2015 | TransitionRx: Impact of community pharmacy postdischarge medication therapy management on hospital readmission rate | Journal of the American Pharmacists Association: JAPhA | 55 | | 3 | 246-54 |
| Madge, P.; McColl, J.; Paton, J. | 1997 | Impact of a nurse-led home management training programme in children admitted to hospital with acute asthma: a randomised controlled study | Thorax | 52 | | 3 | 223-28 |
| Masterson Creber, R.; Patey, M.; Lee, C. S.; Kuan, A.; Jurgens, C.; Riegel, B. | 2016 | Motivational interviewing to improve self-care for patients with chronic heart failure: MITI-HF randomized controlled trial | Patient Education & Counseling | 99 | | 2 | 256-64 |
| McPhee, S. J.; Frank, D. H.; Lewis, C.; Bush, D. E.; Smith, C. R. | 1983 | Influence of a "discharge interview" on patient knowledge, compliance, and functional status after hospitalization | Medical Care | 21 | | 8 | 755-67 |
| Mehralian, H.; Salehi, S.; Moghaddasi, J.; Amiri, M.; Rafiei, H. | 2014 | The comparison of the effects of education provided by nurses on the quality of life in patients with congestive heart failure (CHF) in usual and home-visit cares in Iran | Global Journal of Health Science | 6 | | 3 | 256-60 |
| Melin, A. L.; Bygren, L. O. | 1992 | Efficacy of the rehabilitation of elderly primary health care patients after short-stay hospital treatment | Medical Care | 30 | | 11 | 1004-15 |
| Middleton, S.; Donnelly, N.; Harris, J.; Ward, J. | 2005 | Nursing intervention after carotid endarterectomy: a randomized trial of Co-ordinated Care Post-Discharge (CCPD) | Journal of Advanced Nursing | 52 | | 3 | 250-61 |
| Miller, D. A.; Schaper, A. M. | 2015 | Implementation of a follow-up telephone call process for patients at high risk for readmission | Journal of Nursing Care Quality | 30 | | 1 | 63-70 |
| Miranda, M. B.; Gorski, L. A.; LeFevre, J. G.; Levac, K. A.; Niederstadt, J. A.; Toy, A. L. | 2002 | An evidence-based approach to improving care of patients with heart failure across the continuum | Journal of Nursing Care Quality | 17 | | 1 | 1-14 |
| Mukamel, D. B.; Amin, A.; Weimer, D. L.; Ladd, H.; Sharit, J.; Schwarzkopf, R.; Sorkin, D. H. | 2016 | Personalizing Nursing Home Compare and the Discharge from Hospitals to Nursing Homes | Health Services Research | 51 | | 6 | 2076-94 |
| Nagle, D.; Pare, T.; Keenan, E.; Marcet, K.; Tizio, S.; Poylin, V. | 2012 | Ileostomy pathway virtually eliminates readmissions for dehydration in new ostomates | Diseases of the Colon & Rectum | 55 | | 12 | 1266-72 |
| Naylor, M. D.; McCauley, K. M. | 1999 | The effects of a discharge planning and home follow-up intervention on elders hospitalized with common medical and surgical cardiac conditions | Journal of Cardiovascular Nursing | 14 | | 1 | 44-54 |
| Nazareth, I.; Burton, A.; Shulman, S.; Smith, P.; Haines, A.; Timberal, H. | 2001 | A pharmacy discharge plan for hospitalized elderly patients-a randomized controlled trial | Age & Ageing | 30 | | 1 | 33-40 |
| Nguyen, D.; Busey, B.; Stackle, M.; Donoway, T.; Strickland, S.; Roselle, A.; Hahn, S.; Bennett, N. | 2016 | Implementation of a Transition of Care Coordinator at a Military Treatment Facility | US Army Medical Department Journal |  | |  | 47-52 |
| Novak, C. J.; Hastanan, S.; Moradi, M.; Terry, D. F. | 2012 | Reducing unnecessary hospital readmissions: the pharmacist's role in care transitions | Consultant Pharmacist | 27 | | 3 | 174-9 |
| Nuccio, E.; Richard, A. A. | 2010 | Do delays in initiation of home healthcare services following hospital discharge affect patient outcomes? | Home Healthcare Nurse | 28 | | 8 | 500-507 |
| Ohia, Mary Iwalani | 2014 | Improving hospital readmission rates: The benefit of a transitional case management program | ProQuest Dissertations Publishing | Ed.D. | |  |  |
| Olsen, Russell; Courtemanche, Ted; Hodach, Richard | 2016 | Automated Phone Assessments and Hospital Readmissions | Population Health Management | 19 | | 2 | 120-124 |
| Ong, M. K.; Romano, P. S.; Edgington, S.; Aronow, H. U.; Auerbach, A. D.; Black, J. T.; De Marco, T.; Escarce, J. J.; Evangelista, L. S.; Hanna, B.; Ganiats, T. G.; Greenberg, B. H.; Greenfield, S.; Kaplan, S. H.; Kimchi, A.; Liu, H.; Lombardo, D.; Mangione, C. M.; Sadeghi, B.; Sadeghi, B.; Sarrafzadeh, M.; Tong, K.; Fonarow, G. C.; Better Effectiveness After Transition-Heart Failure Research, Group | 2016 | Effectiveness of Remote Patient Monitoring After Discharge of Hospitalized Patients With Heart Failure: The Better Effectiveness After Transition - Heart Failure (BEAT-HF) Randomized Clinical Trial | JAMA Internal Medicine | 176 | | 3 | 310-8 |
| Ornstein, K.; Smith, K. L.; Foer, D. H.; Lopez-Cantor, M. T.; Soriano, T. | 2011 | To the hospital and back home again: a nurse practitioner-based transitional care program for hospitalized homebound people | Journal of the American Geriatrics Society | 59 | | 3 | 544-51 |
| Paquette, Julie; Le May, Sylvie; Lachance Fiola, Jacinthe; Villeneuve, Edith; Lapointe, Annie; Bourgault, Patricia | 2013 | A randomized clinical trial of a nurse telephone follow-up on paediatric tonsillectomy pain management and complications | Journal of Advanced Nursing | 69 | | 9 | 2054-65 |
| Park, H. K.; Branch, L. G.; Bulat, T.; Vyas, B. B.; Roever, C. P. | 2013 | Influence of a transitional care clinic on subsequent 30-day hospitalizations and emergency department visits in individuals discharged from a skilled nursing facility | Journal of the American Geriatrics Society | 61 | | 1 | 137-42 |
| Parry, M. J.; Watt-Watson, J.; Hodnett, E.; Tranmer, J.; Dennis, C. L.; Brooks, D. | 2009 | Cardiac Home Education and Support Trial (CHEST): a pilot study | Canadian Journal of Cardiology | 25 | | 12 | e393-8 |
| Phatak, A.; Prusi, R.; Ward, B.; Hansen, L. O.; Williams, M. V.; Vetter, E.; Chapman, N.; Postelnick, M. | 2016 | Impact of pharmacist involvement in the transitional care of high-risk patients through medication reconciliation, medication education, and postdischarge call-backs (IPITCH Study) | Journal of Hospital Medicine | 11 | | 1 | 39-44 |
| Polak, Tracy; Ahuja, Namita; Milanovich, Penny; Wickline, Dennis | 2016 | Enhanced Care Program for Transitional Care to Home Upon Acute Care or SNF Discharge: A Quality Improvement Study | Journal of the American Medical Directors Association | 17 | | 3 | B15-B15 |
| Polinski, Jennifer M.; Moore, Janice M.; Kyrychenko, Pavlo; Gagnon, Michael; Matlin, Olga S.; Fredell, Joshua W.; Brennan, Troyen A.; Shrank, William H. | 2016 | An Insurer's Care Transition' Program Emphasizes Medication Reconciliation, Reduces Readmissions And Costs | Health Affairs | 35 | | 7 | 1222-29 |
| Poston, K. M.; Dumas, B. P.; Edlund, B. J. | 2014 | Outcomes of a quality improvement project implementing stroke discharge advocacy to reduce 30-day readmission rates | Journal of Nursing Care Quality | 29 | | 3 | 237-44 |
| Ramani, A. A.; Pickston, A. A.; Clark, J. L.; Clark, C. A.; Brown, M. | 2010 | Role of the management pathway in the care of advanced COPD patients in their own homes | Care Management Journals | 11 | | 4 | 249-53 |
| Rankin, S. H. | 2002 | An audiotaped information programme after coronary artery bypass surgery improved physical functioning in women and psychological distress in men | Evidence Based Nursing | 5 | | 1 | 17 |
| Rauh, R. A.; Schwabauer, N. J.; Enger, E. L.; Moran, J. F. | 1999 | A community hospital-based congestive heart failure program: impact on length of stay, admission and readmission rates, and cost | American Journal of Managed Care | 5 | | 1 | 37-43 |
| Reynolds, M. A. | 2009 | Postoperative pain management discharge teaching in a rural population | Pain Management Nursing | 10 | | 2 | 76-84 |
| Rich, M. W.; Beckham, V.; Wittenberg, C.; Leven, C. L.; Freedland, K. E.; Carney, R. M. | 1995 | A multidisciplinary intervention to prevent the readmission of elderly patients with congestive heart failure | New England Journal of Medicine | 333 | | 18 | 1190-5 |
| Robinson, T. E.; Zhou, L.; Kerse, N.; Scott, J. D.; Christiansen, J. P.; Holland, K.; Armstrong, D. E.; Bramley, D. | 2015 | Evaluation of a New Zealand program to improve transition of care for older high risk adults | Australasian Journal on Ageing | 34 | | 4 | 269-74 |
| Roselle, S.; D'Amico, F. J. | 1982 | The effect of home respiratory therapy on hospital readmission rates of patients with chronic obstructive pulmonary disease | Respiratory Care | 27 | | 10 | 1194-9 |
| Ryan, T.; Enderby, P.; Rigby, A. S. | 2006 | A randomized controlled trial to evaluate intensity of community-based rehabilitation provision following stroke or hip fracture in old age | Clinical Rehabilitation | 20 | | 2 | 123-31 |
| Saleh, S. S.; Freire, C.; Morris-Dickinson, G.; Shannon, T. | 2012 | An effectiveness and cost-benefit analysis of a hospital-based discharge transition program for elderly Medicare recipients | Journal of the American Geriatrics Society | 60 | | 6 | 1051-6 |
| Sanguinetti, M.; Catanzaro, M. | 1987 | A comparison of discharge teaching on the consequences of brain injury | Journal of Neuroscience Nursing | 19 | | 5 | 271-5 |
| Sanii, Y.; Torkamandi, H.; Gholami, K.; Hadavand, N.; Javadi, M. | 2016 | Role of pharmacist counseling in pharmacotherapy quality improvement | Journal of Research in Pharmacy Practice | 5 | | 2 | 132-7 |
| Schnipper, J. L.; Kirwin, J. L.; Cotugno, M. C.; Wahlstrom, S. A.; Brown, B. A.; Tarvin, E.; Kachalia, A.; Horng, M.; Roy, C. L.; McKean, S. C.; Bates, D. W. | 2006 | Role of pharmacist counseling in preventing adverse drug events after hospitalization | Archives of Internal Medicine | 166 | | 5 | 565-71 |
| Schweitzer, M.; Aucoin, J.; Docherty, S. L.; Rice, H. E.; Thompson, J.; Sullivan, D. T. | 2014 | Evaluation of a discharge education protocol for pediatric patients with gastrostomy tubes | Journal of Pediatric Health Care | 28 | | 5 | 420-8 |
| Scullin, C.; Scott, M. G.; Hogg, A.; McElnay, J. C. | 2007 | An innovative approach to integrated medicines management | Journal of Evaluation in Clinical Practice | 13 | | 5 | 781-8 |
| Seggelke, S. A.; Hawkins, R. M.; Gibbs, J.; Rasouli, N.; Wang, C.; Draznin, B. | 2014 | Transitional care clinic for uninsured and medicaid-covered patients with diabetes mellitus discharged from the hospital: a pilot quality improvement study | Hospital Practice | 42 | | 1 | 46-51 |
| Setter, S. M.; Corbett, C. F.; Neumiller, J. J.; Gates, B. J.; Sclar, D. A.; Sonnett, T. E. | 2009 | Effectiveness of a pharmacist--nurse intervention on resolving medication discrepancies for patients transitioning from hospital to home health care | American Journal of Health-System Pharmacy | 66 | | 22 | 2027-31 |
| Shaya, F. T.; Chirikov, V. V.; Rochester, C.; Zaghab, R. W.; Kucharski, K. C. | 2015 | Impact of a comprehensive pharmacist medication-therapy management service | Journal of Medical Economics | 18 | | 10 | 828-37 |
| Smeenk, F. W.; de Witte, L. P.; van Haastregt, J. C.; Schipper, R. M.; Biezemans, H. P.; Crebolder, H. F. | 1998 | Transmural care. A new approach in the care for terminal cancer patients: its effects on re-hospitalization and quality of life | Patient Education & Counseling | 35 | | 3 | 189-99 |
| Smith, D. M.; Weinberger, M.; Katz, B. P.; Moore, P. S. | 1988 | Postdischarge care and readmissions | Medical Care | 26 | | 7 | 699-708 |
| Sovie, M. D.; Tarcinale, M. A.; Burns, K. A.; Jacoby, F. G.; Kull, J. A.; Tringali, R. A.; Kramer-Bodnar, C. | 1983 | Discharge planning and home care... to prepare hospital and community health nurses for their roles in the discharge and home care of a burned person | Journal of Burn Care & Rehabilitation | 4 | | 6 | 458-65 |
| Stafford, L.; van Tienen, E. C.; Bereznicki, L. R.; Peterson, G. M. | 2012 | The benefits of pharmacist-delivered warfarin education in the home | International Journal of Pharmacy Practice | 20 | | 6 | 384-9 |
| Steeman, E.; Moons, P.; Milisen, K.; De Bal, N.; De Geest, S.; De Froidmont, C.; Tellier, V.; Gosset, C.; Abraham, I. | 2006 | Implementation of discharge management for geriatric patients at risk of readmission or institutionalization | International Journal for Quality in Health Care | 18 | | 5 | 352-8 |
| Stewart, S.; Pearson, S.; Horowitz, J. D. | 1998 | Effects of a home-based intervention among patients with congestive heart failure discharged from acute hospital care | Archives of Internal Medicine | 158 | | 10 | 1067-72 |
| Still, K. L.; Davis, A. K.; Chilipko, A. A.; Jenkosol, A.; Norwood, D. K. | 2013 | Evaluation of a pharmacy-driven inpatient discharge counseling service: impact on 30-day readmission rates | Consultant Pharmacist | 28 | | 12 | 775-85 |
| Suter-Gut, D.; Metcalf, A. M.; Donnelly, M. A.; Smith, I. M. | 1990 | Post-discharge care planning and rehabilitation of the elderly surgical patient | Clinics in Geriatric Medicine | 6 | | 3 | 669-83 |
| Tanguturi, V. K.; Temin, E.; Yeh, R. W.; Thompson, R. W.; Rao, S. K.; Mallick, A.; Cavallo, E.; Ferris, T. G.; Wasfy, J. H. | 2016 | Clinical Interventions to Reduce Preventable Hospital Readmission After Percutaneous Coronary Intervention | Circulation. Cardiovascular Quality & Outcomes | 9 | | 5 | 600-4 |
| Tecson, K. M.; Silver, M. A.; Brune, S. D.; Cauthen, C.; Kwan, M. D.; Schussler, J. M.; Vasudevan, A.; Watts, J. A.; McCullough, P. A. | 2016 | Impact of Enhanced External Counterpulsation on Heart Failure Rehospitalization in Patients With Ischemic Cardiomyopathy | American Journal of Cardiology | 117 | | 6 | 901-5 |
| Tedesco, G. W.; McConaha, J. L.; Skomo, M. L.; Higginbotham, S. K. | 2016 | A Pharmacist's Impact on 30-Day Readmission Rates When Compared to the Current Standard of Care Within a Patient-Centered Medical Home: A Pilot Study | Journal of Pharmacy Practice | 29 | | 4 | 368-73 |
| Thygesen, L. C.; Fokdal, S.; Gjorup, T.; Taylor, R. S.; Zwisler, A. D.; Prevention of Early Readmission Research, Group | 2015 | Can municipality-based post-discharge follow-up visits including a general practitioner reduce early readmission among the fragile elderly (65+ years old)? A randomized controlled trial | Scandinavian Journal of Primary Health Care | 33 | | 2 | 65-73 |
| Tinetti, M. E.; Charpentier, P.; Gottschalk, M.; Baker, D. I. | 2012 | Effect of a restorative model of posthospital home care on hospital readmissions | Journal of the American Geriatrics Society | 60 | | 8 | 1521-6 |
| Torisson, G.; Minthon, L.; Stavenow, L.; Londos, E. | 2013 | Multidisciplinary intervention reducing readmissions in medical inpatients: a prospective, non-randomized study | Clinical Interventions In Aging | 8 | |  | 1295-304 |
| Verhaegh, K. J.; Buurman, B. M.; Veenboer, G. C.; de Rooij, S. E.; Geerlings, S. E. | 2014 | The implementation of a comprehensive discharge bundle to improve the discharge process: a quasi-experimental study | Netherlands Journal of Medicine | 72 | | 6 | 318-25 |
| Vesterlund, M.; Granger, B.; Thompson, T. J.; Coggin, C., 3rd; Oermann, M. H. | 2015 | Tailoring your heart failure project for success in rural areas | Quality Management in Health Care | 24 | | 2 | 91-95 |
| Villars, HÃ©lÃ¨ne; Dupuy, Charlotte; Soler, Pauline; Gardette, Virginie; Soto, Maria E.; Gillette, Sophie; Nourhashemi, Fati; Brunovellas, | 2013 | A follow-up intervention in severely demented patients after discharge from a special Alzheimer acute care unit: impact on early emergency room re-hospitalization rate | International Journal of Geriatric Psychiatry | 28 | | 11 | 1131-1140 |
| Walker, P. C.; Bernstein, S. J.; Jones, J. N.; Piersma, J.; Kim, H. W.; Regal, R. E.; Kuhn, L.; Flanders, S. A. | 2009 | Impact of a pharmacist-facilitated hospital discharge program: a quasi-experimental study | Archives of Internal Medicine | 169 | | 21 | 2003-10 |
| Warden, B. A.; Freels, J. P.; Furuno, J. P.; Mackay, J. | 2014 | Pharmacy-managed program for providing education and discharge instructions for patients with heart failure | American Journal of Health-System Pharmacy | 71 | | 2 | 134-39 |
| Warrington, D.; Cholowski, K.; Peters, D. | 2003 | Effectiveness of home-based cardiac rehabilitation for special needs patients | Journal of Advanced Nursing | 41 | | 2 | 121-29 |
| Warsame, R.; Kasi, P. M.; Villasboas-Bisneto, J. C.; Gallenberg, D.; Wolf, R.; Ward, J.; Matt-Hensrud, N.; Grethen, K.; Colborn, L.; Zeldenrust, S.; Lacy, M. Q.; Thompson, C. A. | 2016 | Transition of Care for Inpatient Hematology Patients Receiving Chemotherapy: Development of Hospital Discharge Huddle Process and Effects of Implementation | Journal of oncology practice/American Society of Clinical Oncology | 12 | | 1 | e88-e94 |
| Wehbe-Janek, H.; Hochhalter, A. K.; Castilla, T.; Jo, C. | 2015 | Feasibility of "Standardized Clinician" Methodology for Patient Training on Hospital-to-Home Transitions | Simulation in Healthcare: The Journal of The Society for Medical Simulation | 10 | | 1 | 4-13 |
| Weintraub, A.; Gregory, D.; Patel, A. R.; Levine, D.; Venesy, D.; Perry, K.; Delano, C.; Konstam, M. A. | 2010 | A multicenter randomized controlled evaluation of automated home monitoring and telephonic disease management in patients recently hospitalized for congestive heart failure: the SPAN-CHF II trial | Journal of Cardiac Failure | 16 | | 4 | 285-292 |
| Weisman, D. S.; Bashir, L.; Mehta, A.; Bhatia, L.; Levine, S. M.; Mete, M.; Padmore, J. S. | 2012 | A medical resident post-discharge phone call study | Hospital Practice | 40 | | 2 | 138-46 |
| Whitby, M.; McLaws, M. L.; Doidge, S.; Collopy, B. | 2007 | Post-discharge surgical site surveillance: does patient education improve reliability of diagnosis? | Journal of Hospital Infection | 66 | | 3 | 237-42 |
| Williams, E. I.; Greenwell, J.; Groom, L. M. | 1992 | The care of people over 75 years old after discharge from hospital: an evaluation of timetabled visiting by Health Visitor Assistants | Journal of Public Health Medicine | 14 | | 2 | 138-44 |
| Williams, N. | 2003 | Nurse led transitional care improved health related quality of life and reduced emergency department use for heart failure | Evidence Based Nursing | 6 | | 1 | 21-21 |
| Young, W.; Rewa, G.; Goodman, S. G.; Jaglal, S. B.; Cash, L.; Lefkowitz, C.; Coyte, P. C. | 2003 | Evaluation of a community-based inner-city disease management program for postmyocardial infarction patients: a randomized controlled trial | Canadian Medical Association Journal (CMAJ) | 169 | | 9 | 905-10 |
| Young, L.; Hertzog, M.; Barnason, S. | 2016 | Effects of a home-based activation intervention on self-management adherence and readmission in rural heart failure patients: the PATCH randomized controlled trial | BMC Cardiovascular Disorders | 16 | | 1 | 176 |
| Ahmed, A. | 2002 | Quality and outcomes of heart failure care in older adults: role of multidisciplinary disease-management programs | Journal of the American Geriatrics Society | 50 | | 9 | 1590-1593 |
| Alkubati, S. A.; Al-Zaru, I. M.; Khater, W.; Ammouri, A. A. | 2013 | Perceived learning needs of Yemeni patients after coronary artery bypass graft surgery | Journal of Clinical Nursing | 22 | | 7-Aug | 930-8 |
| Alonso-Babarro, A.; Bruera, E.; Varela-Cerdeira, M.; Boya-Cristia, M. J.; Madero, R.; Torres-Vigil, I.; De Castro, J.; Gonzalez-Baron, M. | 2011 | Can this patient be discharged home? Factors associated with at-home death among patients with cancer | Journal of Clinical Oncology | 29 | | 9 | 1159-67 |
| Anderson, C. A. | 1979 | Making the right moves in discharge planning: home or nursing home? Let the elderly patient decide | American Journal of Nursing | 79 | | 8 | 1448-9 |
| Andrews, E.; Toubman, S. | 2009 | Patient-centered medical home: improving health care by shifting the focus to patients | Connecticut Medicine | 73 | | 8 | 479-80 |
| Annis, T. D. | 2002 | The Synergy Model in practice. The interdisciplinary team across the continuum of care | Critical Care Nurse | 22 | | 5 | 76-79 |
| Anonymous, | 1992 | Smoothing the transition from hospital to home. Patient Learning Center increases quality of care. University of Minnesota Hospital and Clinic | Profiles in Healthcare Marketing |  | | 47 |  |
| Anonymous, | 2013 | Study: interventions help prevent readmissions | Hospital Case Management | 21 | | 9 | 122, 127-8 |
| Anonymous, | 2015 | Star Ratings show hospitals need to improve the discharge process | Hospital Case Management | 23 | | 7 | 81-3 |
| Aoughsten, Jacqueline; Johnson, Susan; Kuruvilla, Mercy; Bionat, Susan | 2015 | The Effect of the Affordable Care Act on Medicare: Opportunities for Advanced Practice Nursing | Nurse Leader | 13 | | 3 | 49-53 |
| Arena, R.; Williams, M.; Forman, D. E.; Cahalin, L. P.; Coke, L.; Myers, J.; Hamm, L.; Kris-Etherton, P.; Humphrey, R.; Bittner, V.; Lavie, C. J.; American Heart Association Exercise, Cardiac Rehabilitation; Prevention Committee of the Council on Clinical Cardiology, Council on Epidemiology; Prevention,; Council on Nutrition, Physical Activity; Metabolism, | 2012 | Increasing referral and participation rates to outpatient cardiac rehabilitation: the valuable role of healthcare professionals in the inpatient and home health settings: a science advisory from the American Heart Association | Circulation | 125 | | 10 | 1321-9 |
| Arora, V. M.; Prochaska, M. L.; Farnan, J. M.; D'Arcy, M. J. th; Schwanz, K. J.; Vinci, L. M.; Davis, A. M.; Meltzer, D. O.; Johnson, J. K. | 2010 | Problems after discharge and understanding of communication with their primary care physicians among hospitalized seniors: a mixed methods study | Journal of Hospital Medicine (Online) | 5 | | 7 | 385-91 |
| Askren-Gonzalez, Angela; Frate, Jeff | 2012 | Case Management Programs for Hospital Readmission Prevention | Professional Case Management | 17 | | 5 | 219-226 |
| Baier, Rosa; D'Antonio, Karen; Mottshaw, Paula; Voss, Rachel; Goldsmith, Christopher | 2011 | Transforming transitions from patient interventions to systems change: Quality partners of Rhode Island's safe transitions project | Remington Report | 19 | | 2 |  |
| Bailey, R.; Caldwell, C. | 1997 | Preparing parents for going home | Paediatric Nursing | 9 | | 4 | 15-17 |
| Balsam, A.; Osteraas, G. | 1987 | Developing a continuum of community nutrition services: Massachusetts elderly nutrition programs | Journal of Nutrition for the Elderly | 6 | | 4 | 51-67 |
| Barnes, S. | 2000 | Ambulatory surgery. Are you watching the clock? Let criteria define discharge readiness | Journal of Peri Anaesthesia Nursing | 15 | | 3 | 174-176 |
| Bates, David; Brennan, Patricia Flatley; Flory, Joyce | 2015 | Leveraging Evidence Across the Care Continuum | Joint Commission Journal on Quality & Patient Safety | 41 | | 2 | 87-96 |
| Bell, Susan P.; Vasilevskis, Eduard E.; Saraf, Avantika A.; Jacobsen, J. M. L.; Kripalani, Sunil; Mixon, Amanda S.; Schnelle, John F.; Simmons, Sandra F. | 2016 | Geriatric Syndromes in Hospitalized Older Adults Discharged to Skilled Nursing Facilities | Journal of the American Geriatrics Society | 64 | | 4 | 715-722 |
| Berryman, Sandra N.; Palmer, Sheri P.; Kohl, James E.; Parham, Jon S. | 2013 | Medical Home Model of Patient-Centered Health Care | Medsurg Nursing | 22 | | 3 | 166-196 |
| Bielaszka-DuVernay, Christina | 2011 | The 'GRACE' Model: In-Home Assessments Lead to Better Care for Dual Eligibles | Health Affairs | 30 | | 3 | 431-434 |
| Birmingham, J.; Carter, J.; Commander, C.; Holland, D. E.; Skinner, N. | 2008 | Provide information to ensure patients safely transition through the continuum | Hospital Case Management | 16 | | 9 | 129-131 |
| Black, J. T.; Romano, P. S.; Sadeghi, B.; Auerbach, A. D.; Ganiats, T. G.; Greenfield, S.; Kaplan, S. H.; Ong, M. K.; Beat-Hf Research Group | 2014 | A remote monitoring and telephone nurse coaching intervention to reduce readmissions among patients with heart failure: study protocol for the Better Effectiveness After Transition - Heart Failure (BEAT-HF) randomized controlled trial | Trials | 15 | |  | 124 |
| Bloomquist, L. M.; Lewis-Hunstiger, M. J. | 1978 | To care for the child at home: discharge planning for the child with leukaemia | Cancer Nursing | 1 | | 4 | 303-8 |
| Bobay, K. L.; Jerofke, T. A.; Weiss, M. E.; Yakusheva, O. | 2010 | Age-related differences in perception of quality of discharge teaching and readiness for hospital discharge | Geriatric Nursing | 31 | | 3 | 178-187 |
| Borders, C. R. | 1985 | Preparing for posthospital home care. Home care related patient education | Patient Care | 19 | | 21 | 27-36 |
| Bowles, K. H. | 2000 | Patient problems and nurse interventions during acute care and discharge planning | Journal of Cardiovascular Nursing | 14 | | 3 | 29-41 |
| Brekke, A.; Elfenbein, D. M.; Madkhali, T.; Schaefer, S. C.; Shumway, C.; Chen, H.; Schneider, D. F.; Sippel, R. S.; Balentine, C. | 2016 | When patients call their surgeon's office: An opportunity to improve the quality of surgical care and prevent readmissions | American Journal of Surgery | 211 | | 3 | 599-604 |
| Brock, Jane; Boutwell, Amy E. | 2012 | How did we make transitions such a big deal? | Generations | 36 | | 4 | 35-43 |
| Brogaard, T.; Jensen, A. B.; Sokolowski, I.; Olesen, F.; Neergaard, M. A. | 2011 | Who is the key worker in palliative home care? | Scandinavian Journal of Primary Health Care | 29 | | 3 | 150-6 |
| Capuano, T. A.; Barber, R.; Sullivan, K. | 1997 | Bridging the care continuum for open heart surgery patients | Nursing Management | 28 | | 2 | 32B-32D |
| Cheng, H. Y.; Tonorezos, E.; Zorowitz, R.; Novotny, J.; Dubin, S.; Maurer, M. S. | 2006 | Inpatient care for nursing home patients: An opportunity to improve transitional care | Journal of the American Medical Directors Association | 7 | | 6 | 383-387 |
| Coleman, E. A.; Chugh, A.; Williams, M. V.; Grigsby, J.; Glasheen, J. J.; McKenzie, M.; Min, S. J. | 2013 | Understanding and execution of discharge instructions | American Journal of Medical Quality | 28 | | 5 | 383-91 |
| Coller, R. J.; Klitzner, T. S.; Saenz, A. A.; Lerner, C. F.; Nelson, B. B.; Chung, P. J. | 2015 | The Medical Home and Hospital Readmissions | Paediatrics | 136 | | 6 | e1550-60 |
| Comin-Colet, J.; Verdu-Rotellar, J. M.; Vela, E.; Cleries, M.; Bustins, M.; Mendoza, L.; Badosa, N.; Cladellas, M.; Ferre, S.; Bruguera, J.; working group of the Integrated Program for Heart Failure Management of the Barcelona Litoral Mar Integrated Health Care Area, Spain | 2014 | Efficacy of an integrated hospital-primary care program for heart failure: A population-based analysis of 56,742 patients | Revista Espanola de Cardiologia | 67 | | 4 | 283-93 |
| Cummings, S. M. | 1999 | Adequacy of discharge plans and rehospitalization among hospitalized dementia patients | Health & Social Work | 24 | | 4 | 249-259 |
| Dancer, S. | 1996 | Redesigning care for the non-hemorrhagic stroke patient | Journal of Neuroscience Nursing | 28 | | 3 | 183-189 |
| Dark, Cedric K.; Matthews, Kameron L.; Cryer, Lesley; Van Amsterdam, Melanie; Leff, Bruce | 2012 | A Randomized Trial Of 'Hospital At Home' | Health Affairs | 31 | | 9 | 2152-2152 |
| Davoody, N.; Koch, S.; Krakau, I.; Hagglund, M. | 2014 | Collaborative interaction points in post-discharge stroke care | International Journal of Integrated Care | 14 | |  | e032 |
| DeMarco, J.; Nystrom, M. S. | 2009 | Expanding education technology to meet the needs of patients, families, and clinicians | Health Promotion Practice | 10 | | 1 | 15-23 |
| Desai, R.; Williams, C. E.; Greene, S. B.; Pierson, S.; Hansen, R. A. | 2011 | Medication errors during patient transitions into nursing homes: characteristics and association with patient harm | American Journal of Geriatric Pharmacotherapy | 9 | | 6 | 413-22 |
| Dettenmeier, P. A. | 1990 | Planning for successful home mechanical ventilation | AACN Clinical Issues in Critical Care Nursing | 1 | | 2 | 267-79 |
| Edwards, P. K.; Jacobs, C. A.; Hadden, K. B.; Barnes, C. L. | 2016 | A Perioperative Patient Support System Was Unable to Mitigate the Risk of Hospital Readmission for Total Hip Arthroplasty Patients with High American Society of Anaesthesiologists Grades | Journal of Arthroplasty | 20 | |  | 20 |
| Elias, E. R.; Murphy, N. A.; Council on Children with, Disabilities | 2012 | Home care of children and youth with complex health care needs and technology dependencies | Paediatrics | 129 | | 5 | 996-1005 |
| Evdokimoff, Merrily | 2011 | One home health agency's quality improvement project to decrease rehospitalizations: utilizing a transitions model | Home Healthcare Nurse | 29 | | 3 | 180-195 |
| Falvey, Jason R.; Burke, Robert E.; Malone, Daniel; Ridgeway, Kyle J.; McManus, Beth M.; Stevens-Lapsley, Jennifer E. | 2016 | Role of physical therapists in reducing hospital readmissions: Optimizing outcomes for older adult during care transitions from hospital to community | Physical Therapy | 96 | | 8 | 1125-1134 |
| Giesy, J. | 1987 | Teaching discharge management. Utilizing a stimulation game: Home Pursuit | Journal of Paediatric Nursing | 2 | | 5 | 353-354 |
| Goldenheim, A.; Oates, D.; Parker, V.; Russell, M.; Winter, M.; Silliman, R. A. | 2014 | Rehospitalization of older adults discharged to home hospice care | Journal of Palliative Medicine | 17 | | 7 | 841-4 |
| Grafft, C. A.; McDonald, F. S.; Ruud, K. L.; Liesinger, J. T.; Johnson, M. G.; Naessens, J. M. | 2010 | Effect of hospital follow-up appointment on clinical event outcomes and mortality | Archives of Internal Medicine | 170 | | 11 | 955-60 |
| Greysen, S. R.; Harrison, J. D.; Kripalani, S.; Vasilevskis, E.; Robinson, E.; Metlay, J.; Schnipper, J. L.; Meltzer, D.; Sehgal, N.; Ruhnke, G. W.; Williams, M. V.; Auerbach, A. D. | 2016 | Understanding patient-centred readmission factors: A multi-site, mixed-methods study | BMJ Quality & Safety | 14 | |  | 14 |
| Hansen, L. O.; Strater, A.; Smith, L.; Lee, J.; Press, R.; Ward, N.; Weigelt, J. A.; Boling, P.; Williams, M. V. | 2011 | Hospital discharge documentation and risk of rehospitalisation | BMJ Quality & Safety | 20 | | 9 | 773-8 |
| Harrison, J. D.; Greysen, R. S.; Jacolbia, R.; Nguyen, A.; Auerbach, A. D. | 2016 | Not ready, not set...discharge: Patient-reported barriers to discharge readiness at an academic medical center | Journal of Hospital Medicine (Online) | 11 | | 9 | 610-4 |
| Jackson, B.; Swanson, C.; Hicks, L. E.; Prokop, L.; Laughlin, J. | 2000 | Bridge of continuity from hospital to nursing home -- part I: a proactive approach to reduce relocation stress syndrome in the elderly | Continuum: An Interdisciplinary Journal on Continuity of Care | 20 | | 1 |  |
| Kalowes, P.; Peters, C.; Long, P.; Hawkins, D.; Wayne, B.; Catipon, K.; Tin, E.; Schauer, A.; Jeong, S.; Lamond, B.; Nguyen, H. | 2011 | 3. Improving patient outcomes in heart failure: Assessment of nurses' knowledge of heart failure self-management | Heart & Lung | 40 | | 4 | 362-362 |
| Kinnersly, D.; Grundy, D.; Russell, J. | 1986 | ABC of spinal cord injury. Transfer of care from hospital to community | British Medical Journal Clinical Research Ed. | 292 | | 6520 | 607-9 |
| Lorimer, K. | 2004 | Happenings. Continuity through best practice: design and implementation of a nurse-led community leg-ulcer service | Canadian Journal of Nursing Research | 36 | | 2 | 105-112 |
| Mabire, C.; Bula, C.; Morin, D.; Goulet, C. | 2015 | Nursing discharge planning for older medical inpatients in Switzerland: A cross-sectional study | Geriatric Nursing | 36 | | 6 | 451-7 |
| McAllister, J. W.; Presler, E.; Turchi, R. M.; Antonelli, R. C. | 2009 | Achieving effective care coordination in the medical home | Paediatric Annals | 38 | | 9 | 491-7 |
| McIlvennan, C. K.; Eapen, Z. J.; Allen, L. A. | 2015 | Hospital readmissions reduction program | Circulation | 131 | | 20 | 1796-803 |
| McKale, Brigitte | 2014 | Reducing hospital readmissions using a multimodal evidence-based approach |  | D.N.P. | |  | 51 |
| McKinney, M. | 2010 | Coaching with care. Patient advocates help guide post-hospital care in an effort to improve outcomes, reduce readmissions | Modern Healthcare | 40 | | 33 | 30-2 |
| Mead, J. | 1994 | An emphasis on practical management. Discharge planning in stoma care | Professional Nurse | 9 | | 6 | 405-6, 408-10 |
| Pfaff, S. J.; Terry, B. A. | 1980 | Discharge planning. Infection prevention and control in the home | Nursing Clinics of North America | 15 | | 4 | 893-908 |
| Shyu, Yea-Ing Lotus; Chen, Min-Chi; Liang, Jersey; Tseng, Ming-Yueh | 2012 | Trends in health outcomes for family caregivers of hip-fractured elders during the first 12 months after discharge | Journal of Advanced Nursing | 68 | | 3 | 658-666 |
| Smith, C. E.; Schorfheide, A. M.; Lackey, N. R. | 1985 | Acute to home care: managing the transition | Kansas Nurse | 60 | | 11 |  |
| Toles, Mark; Young, Heather M.; Ouslander, Joseph | 2012 | Improving care transitions in nursing homes | Generations | 36 | | 4 | 78-85 |
| Toren, O.; Kerzman, H.; Koren, N.; Baron-Epel, O. | 2006 | Patients' knowledge regarding medication therapy and the association with health services utilization | European Journal of Cardiovascular Nursing | 5 | | 4 | 311-6 |
| Tuso, P.; Watson, H. L.; Garofalo-Wright, L.; Lindsay, G.; Jackson, A.; Taitano, M.; Koyama, S.; Kanter, M. | 2014 | Complex case conferences associated with reduced hospital admissions for high-risk patients with multiple comorbidities | Permanente Journal | 18 | | 1 | 38-42 |
| Weiss, M. E.; Piacentine, L. B.; Lokken, L.; Ancona, J.; Archer, J.; Gresser, S.; Holmes, S. B.; Toman, S.; Toy, A.; Vega-Stromberg, T. | 2007 | Perceived readiness for hospital discharge in adult medical-surgical patients | Clinical Nurse Specialist | 21 | | 1 | 31-42 |
| Young, C. K.; White, S. | 1992 | Preparing patients for tube feeding at home | American Journal of Nursing | 92 | | 4 | 46-53 |
| Agarwal, K. S.; Kazim, R.; Xu, J.; Borson, S.; Taffet, G. E. | 2016 | Unrecognized cognitive impairment and its effect on heart failure readmissions of elderly adults | Journal of the American Geriatrics Society | | 64 | 11 | 2296-2301 |
| Akbari, M.; Celik, S. S. | 2015 | The effects of discharge training and counselling on post-discharge problems in patients undergoing coronary artery bypass graft surgery | Iranian Journal of Nursing and Midwifery Research | | 20 | 4 | 442-9 |
| Baptiste, D. L.; Davidson, P.; Groff Paris, L.; Becker, K.; Magloire, T.; Taylor, L. A. | 2016 | Feasibility study of a nurse-led heart failure education program | Contemporary Nurse | | 52 | 4 | 499-510 |
| Boxer, R. S.; Dolansky, M. A.; Frantz, M. A.; Prosser, R.; Hitch, J. A.; Pina, I. L. | 2012 | The Bridge Project: Improving heart failure care in skilled nursing facilities | Journal of the American Medical Directors Association | | 13 | 1 | 83.e1-7 |
| Brook, K.; Camargo, C. A.; Christopher, K. B.; Quraishi, S. A. | 2015 | Admission vitamin D status is associated with discharge destination in critically ill surgical patients | Annals of Intensive Care | | 5 | 1 | 23 |
| Bull, M. J.; Hansen, H. E.; Gross, C. R. | 2000 | A professional-patient partnership model of discharge planning with elders hospitalized with heart failure | Applied Nursing Research | | 13 | 1 | 19-28 |
| Callery, P. | 1998 | A nurse led home management training programme reduced readmissions to the hospital in children with acute asthma | Evidence Based Nursing | | 1 | 1 |  |
| Cawley, J.; Grantham, C. C. | 2011 | Building a system of care: Integration across the heart failure care continuum | Permanente Journal | | 15 | 3 | 37-42 |
| Chaiyawat, P.; Kulkantrakorn, K.; Sritipsukho, P. | 2009 | Effectiveness of home rehabilitation for ischemic stroke | Neurology International | | 1 | 1 | e10 |
| Chaiyawat, P.; Kulkantrakorn, K. | 2012 | Effectiveness of home rehabilitation program for ischemic stroke upon disability and quality of life: A randomized controlled trial | Clinical Neurology & Neurosurgery | | 114 | 7 | 866-70 |
| Chan, K. E.; Lazarus, J. M.; Wingard, R. L.; Hakim, R. M. | 2009 | Association between repeat hospitalization and early intervention in dialysis patients following hospital discharge | Kidney International | | 76 | 3 | 331-41 |
| Chow, S. K.; Wong, F. K.; Chan, T. M.; Chung, L. Y.; Chang, K. K.; Lee, R. P. | 2008 | Community nursing services for post discharge chronically ill patients | Journal of Clinical Nursing | | 17 | 7B | 260-71 |
| Davis, J. L.; Harper, M.; McBroom, K.; Taylor, E.; Pura, L. | 2011 | 20. Heart failure at home sweet home | Heart & Lung | | 40 | 4 | 381-382 |
| Dhalla, I. A.; O'Brien, T.; Morra, D.; Thorpe, K. E.; Wong, B. M.; Mehta, R.; Frost, D. W.; Abrams, H.; Ko, F.; Van Rooyen, P.; Bell, C. M.; Gruneir, A.; Lewis, G. H.; Daub, S.; Anderson, G. M.; Hawker, G. A.; Rochon, P. A.; Laupacis, A. | 2014 | Effect of a post discharge virtual ward on readmission or death for high-risk patients: a randomized clinical trial | JAMA | | 312 | 13 | 1305-12 |
| Diem, S. J.; Prochazka, A. V.; Meyer, T. J.; Fryer, G. E. | 1996 | Effects of a post discharge clinic on house staff satisfaction and utilization of hospital services | Journal of General Internal Medicine | | 11 | 3 | 179-81 |
| Domingues, F. B.; Clausell, N.; Aliti, G. B.; Dominguez, D. R.; Rabelo, E. R. | 2011 | Education and telephone monitoring by nurses of patients with heart failure: randomized clinical trial | Arquivos Brasileiros de Cardiologia | | 96 | 3 | 233-9 |
| Donovan, J. L.; Kanaan, A. O.; Gurwitz, J. H.; Tjia, J.; Cutrona, S. L.; Garber, L.; Preusse, P.; Field, T. S. | 2016 | A pilot health information technology-based effort to increase the quality of transitions from skilled nursing facility to home: Compelling evidence of high rate of adverse outcomes | Journal of the American Medical Directors Association | | 17 | 4 | 312-7 |
| Gurwitz, J. H.; Field, T. S.; Ogarek, J.; Tjia, J.; Cutrona, S. L.; Harrold, L. R.; Gagne, S. J.; Preusse, P.; Donovan, J. L.; Kanaan, A. O.; Reed, G.; Garber, L. | 2014 | An electronic health record-based intervention to increase follow-up office visits and decrease rehospitalization in older adults | Journal of the American Geriatrics Society | | 62 | 5 | 865-71 |
| Hagglund, E.; Lynga, P.; Frie, F.; Ullman, B.; Persson, H.; Melin, M.; Hagerman, I. | 2015 | Patient-centred home-based management of heart failure. Findings from a randomised clinical trial evaluating a tablet computer for self-care, quality of life and effects on knowledge | Scandinavian Cardiovascular Journal | | 49 | 4 | 193-9 |
| Kahokehr, A.; Broadbent, E.; Wheeler, B. R.; Sammour, T.; Hill, A. G. | 2012 | The effect of perioperative psychological intervention on fatigue after laparoscopic cholecystectomy: A randomized controlled trial | Surgical Endoscopy | | 26 | 6 | 1730-6 |
| Kansagara, D.; Ramsay, R. S.; Labby, D.; Saha, S. | 2012 | Post-discharge intervention in vulnerable, chronically ill patients | Journal of Hospital Medicine (Online) | | 7 | 2 | 124-30 |
| Karapinar-Carkit, F.; Borgsteede, S. D.; Zoer, J.; Siegert, C.; van Tulder, M.; Egberts, A. C.; van den Bemt, P. M. | 2010 | The effect of the COACH program (Continuity of Appropriate pharmacotherapy, patient Counselling and information transfer in Healthcare) on readmission rates in a multicultural population of internal medicine patients | BMC Health Services Research | | 10 |  | 39 |
| Latour, C. H.; Bosmans, J. E.; van Tulder, M. W.; de Vos, R.; Huyse, F. J.; de Jonge, P.; van Gemert, L. A.; Stalman, W. A. | 2007 | Cost-effectiveness of a nurse-led case management intervention in general medical outpatients compared with usual care: An economic evaluation alongside a randomized controlled trial | Journal of Psychosomatic Research | | 62 | 3 | 363-70 |
| Lim, W. K.; Lambert, S. F.; Gray, L. C. | 2003 | Effectiveness of case management and post-acute services in older people after hospital discharge | Medical Journal of Australia | | 178 | 6 | 262-6 |
| Linden, B. | 1995 | Evaluation of a home-based rehabilitation programme for patients recovering from acute myocardial infarction | Intensive & Critical Care Nursing | | 11 | 1 |  |
| Markley, Jennifer; Andow, Vanessa; Sabharwal, Karen; Wang, Ziyin; Fennell, Emilie; Dusek, Ron | 2013 | A project to reengineer discharges reduces 30-day readmission rates | American Journal of Nursing | | 113 | 7 | 55-64 |
| McCoy, M. L.; Davidhizar, R.; Gillum, D. R. | 2007 | A correlational pilot study of home health nurse management of heart failure patients and hospital readmissions | Home Health Care Management & Practice | | 19 | 5 | 392-396 |
| Ong, Michael K.; Romano, Patrick S.; Edgington, Sarah; Aronow, Harriet U.; Auerbach, Andrew D.; Black, Jeanne T.; De Marco, Teresa; Escarce, Jose J.; Evangelista, Lorraine S.; Hanna, Barbara; Ganiats, Theodore G.; Greenberg, Barry H.; Greenfield, Sheldon; Kaplan, Sherrie H.; Kimchi, Asher; Honghu, Liu; Lombardo, Dawn; Mangione, Carol M.; Sadegh, Bahman; Sadeghi, Banafsheh | 2016 | Effectiveness of remote patient monitoring after discharge of hospitalized patients with heart failure: the better effectiveness after transition -- heart failure (BEAT-HF) randomized clinical trial | JAMA Internal Medicine | | 176 | 3 | 310-318 |
| Pollard, J.; Oliver-McNeil, S.; Patel, S.; Mason, L.; Baker, H. | 2015 | Impact of the development of a regional collaborative to reduce 30-day heart failure readmissions | Journal of Nursing Care Quality | | 30 | 4 | 298-305 |
| Siehr, S. L.; Norris, J. K.; Bushnell, J. A.; Ramamoorthy, C.; Reddy, V. M.; Hanley, F. L.; Wright, G. E. | 2014 | Home monitoring program reduces inter stage mortality after the modified Norwood procedure | Journal of Thoracic & Cardiovascular Surgery | | 147 | 2 | 718-23.e1 |
| Slater, M. R.; Phillips, D. M.; Woodard, E. K. | 2008 | Cost-effective care a phone call away: A nurse-managed telephonic program for patients with chronic heart failure | Nursing Economics | | 26 | 1 | 41-4 |
| Taber, D. J.; Pilch, N. A.; McGillicuddy, J. W.; Bratton, C. F.; Chavin, K. D.; Baliga, P. K. | 2013 | Improved patient safety and outcomes with a comprehensive interdisciplinary improvement initiative in kidney transplant recipients | American Journal of Medical Quality | | 28 | 2 | 103-12 |
| Williams, H.; Blue, B.; Langlois, P. F. | 1994 | Do follow-up home visits by military nurses of chronically ill medical patients reduce readmissions? | Military Medicine | | 159 | 2 | 141-4 |
| Young, J.; O'Connell, B. | 2001 | Recovery following laparoscopic cholecystectomy in either a 23 hour or an 8-hour facility | Journal of Quality in Clinical Practice | | 21 | 1-Feb | 2-7, 8 |

**No Full Text (n=5)**

| **Authors** | **Year** | **Title** | **Journal** | **Volume** | **Issue** | **Pages** |
| --- | --- | --- | --- | --- | --- | --- |
| Cartwright, G. | 1995 | Home care RCPs play important role in patient and family education | AARC Times | 19 | 10 | 34-50 |
| Cheah, G. M.; Martens, K. H. | 2003 | Coumadin knowledge deficits: do recently hospitalized patients know how to safely manage the medication? | Home Healthcare Nurse | 21 | 2 | 94-100; quiz 101 |
| Dungan, K.; Lyons, S.; Manu, K.; Kulkarni, M.; Ebrahim, K.; Grantier, C.; Harris, C.; Black, D.; Schuster, D. | 2014 | An individualized inpatient diabetes education and hospital transition program for poorly controlled hospitalized patients with diabetes | Endocrine Practice | 20 | 12 | 1265-73 |
| Rice, Y. B.; Barnes, C. A.; Rastogi, R.; Hillstrom, T. J.; Steinkeler, C. N. | 2016 | Tackling 30-Day, All-Cause Readmissions with a Patient-Centered Transitional Care Bundle | Population Health Management | 19 | 1 | 56-62 |
| Valle, R.; Carbonieri, E.; Tenderini, P.; Zanella, C.; De Cian, F.; Ginocchio, G.; Cannas, S.; Milan, D.; Milani, L. | 2004 | A comprehensive management system for heart failure improves clinical outcomes and reduces medical resource utilization | Italian Heart Journal Supplement | 5 | 4 | 282-91 |

**Non English or Non French (n=9)**

| **Authors** | **Year** | **Title** | **Journal** | **Volume** | **Issue** | **Pages** |
| --- | --- | --- | --- | --- | --- | --- |
| Angelino, E.; Fattirolli, F. | 2012 | The bare minimum of information at discharge after acute coronary syndrome. Part 1: Factors that affect communication | Monaldi Archives for Chest Disease | 78 | 2 | 79-84 |
| Andersen, H. E.; Schultz-Larsen Jurgensen, K.; Kreiner, S.; Forchhammer, B. H.; Eriksen, K.; Brown, A. | 2001 | Can readmission after apoplexy be prevented? Post-hospital follow-up intervention for apoplexy patients | Ugeskrift for Laeger | 163 | 46 | 6421-27 |
| Ando, S. | 1986 | Health instruction following discharge with consideration toward personal relationship between the patient and the person in charge of nursing at home | Kangogaku Zasshi – Japanese Journal of Nursing | 50 | 5 | 512-16 |
| Araki, C.; Nakanishi, N.; Akanuma, M.; Shiota, C.; Osawa, M. | 1984 | Nursing care of patients undergoing lung lobectomy and home care instructions | Kango Gijutsu – Japanese Journal of Nursing Art | 30 | 3 | 326-31 |
| Bergenholtz, H. | 2009 | Optimal admission care plan for COPD patients | Sygeplejersken / Danish Journal of Nursing | 109 | 10 | 54-58 |
| Choi, Y. S.; Kim, D. H.; Storey, M.; Kim, C. J.; Kang, K. S. | 1992 | A study of home care needs of patients at discharge and effects of home care-centered on patients discharged from a rural general hospital | Taehan Kanho – Korean Nurse | 31 | 4 | 77-99 |
| de Haro FernÃ¡ndez, Francisco; Flores AntigÃ¼edad, MarÃ­a de la Luz | 2012 | Evaluation of the educational session conducted by a carer hospital nursing care management | Gerokomos | 23 | 4 | 160-165 |
| Falces, C.; Lopez-Cabezas, C.; Andrea, R.; Arnau, A.; Ylla, M.; Sadurni, J. | 2008 | An educative intervention to improve treatment compliance and to prevent readmissions of elderly patients with heart failure | Medicina Clinica | 131 | 12 | 452-56 |
| Sanchez Ulayar, A.; Gallardo Lopez, S.; Pons Llobet, N.; Murgadella Sancho, A.; Campins Bernadas, L.; Merino Mendez, R. | 2012 | Pharmaceutical intervention upon hospital discharge to strengthen understanding and adherence to pharmacological treatment | Farmacia Hospitalaria | 36 | 3 | 118-23 |

**Not Related to Care Transitions (n=47)**

| **Authors** | **Year** | **Title** | **Journal** | **Volume** | **Issue** | **Pages** |
| --- | --- | --- | --- | --- | --- | --- |
| Afendulis, Christopher C.; Caudry, Daryl J.; O'Malley, A. James; Kemper, Peter; Grabowski, David C. | 2016 | Green House adoption and nursing home quality | Health Services Research | 51 |  | 454-474 |
| Aiken, L. S.; Butner, J.; Lockhart, C. A.; Volk-Craft, B. E.; Hamilton, G.; Williams, F. G. | 2006 | Outcome evaluation of a randomized trial of the Phoenix Care intervention: program of case management and coordinated care for the seriously chronically ill | Journal of Palliative Medicine | 9 | 1 | 111-126 |
| Appleby, S. | 2013 | Shared care, home haemodialysis and the expert patient |  | 39 Suppl 1 |  | 16-21 |
| Bager, P.; Vilstrup, H. | 2010 | Discharge of patients with harmful use of alcohol - a randomized trial | Danish Journal of Nursing | 110 | 15 | 62-66 |
| Bakker, F. C.; Persoon, A.; Bredie, S. J.; van Haren-Willems, J.; Leferink, V. J.; Noyez, L.; Schoon, Y.; Olde Rikkert, M. G. | 2014 | The CareWell in Hospital program to improve the quality of care for frail elderly inpatients: results of a before-after study with focus on surgical patients | American Journal of Surgery | 208 | 5 | 735-46 |
| Basch, Ethan; Deal, Allison M.; Kris, Mark G.; Scher, Howard I.; Hudis, Clifford A.; Sabbatini, Paul; Rogak, Lauren; Bennett, Antonia V.; Dueck, Amylou C.; Atkinson, Thomas M.; Chou, Joanne F.; Dulko, Dorothy; Sit, Laura; Barz, Allison; Novotny, Paul; Fruscione, Michael; Sloan, Jeff A.; Schrag, Deborah | 2016 | Symptom Monitoring with Patient-Reported Outcomes During Routine Cancer Treatment: A Randomized Controlled Trial | Journal of Clinical Oncology | 34 | 6 | 557-565 |
| Batchelor, F. A.; Hill, K. D.; Mackintosh, S. F.; Said, C. M.; Whitehead, C. H. | 2012 | Effects of a multifactorial falls prevention program for people with stroke returning home after rehabilitation: A randomized controlled trial | Archives of Physical Medicine & Rehabilitation | 93 | 9 | 1648-55 |
| Bell, R. | 1996 | Continuity of care: were we providing continuity of care for our clients? | Perspectives: The Journal of the Gerontological Nursing Association | 20 | 4 | 17-20 |
| Bento, C. A.; Pedroso, E. R. | 2010 | Assessment of the effectiveness of a home-based care program for patients coinfected with tuberculosis and human immunodeficiency virus after discharge from a reference hospital in South-Eastern Brazil | Brazilian Journal of Infectious Diseases | 14 | 6 | 594-600 |
| Borenstein, J.; Aronow, H. U.; Bolton, L. B.; Choi, J.; Bresee, C.; Braunstein, G. D. | 2013 | Early recognition of risk factors for adverse outcomes during hospitalization among Medicare patients: a prospective cohort study | BMC Geriatrics | 13 |  | 72 |
| Brennan, P. F.; Casper, G. R.; Burke, L. J.; Johnson, K. A.; Brown, R.; Valdez, R. S.; Sebern, M.; Perez, O. A.; Sturgeon, B. | 2010 | Technology-enhanced practice for patients with chronic cardiac disease: home implementation and evaluation | Heart & Lung | 39 | 6 Suppl | S34-46 |
| Brewer, L.; Mellon, L.; Hall, P.; Dolan, E.; Horgan, F.; Shelley, E.; Hickey, A.; Williams, D.; Aspire- S. Study Group | 2015 | Secondary prevention after ischaemic stroke: the ASPIRE-S study | BMC Neurology | 15 |  | 216 |
| Cady, R. G.; Erickson, M.; Lunos, S.; Finkelstein, S. M.; Looman, W.; Celebreeze, M.; Garwick, A. | 2015 | Meeting the needs of children with medical complexity using a telehealth advanced practice registered nurse care coordination model | Maternal & Child Health Journal | 19 | 7 | 1497-506 |
| Carew, A. P.; Resnick, B. | 2015 | Outcomes of the Maryland person-centered hospital discharge program: A pilot targeting decreasing long-term care use and hospital readmissions | Care Management Journals | 16 | 1 | 48-58 |
| Chang, K.; Davis, R.; Birt, J.; Castelluccio, P.; Woodbridge, P.; Marrero, D. | 2007 | Nurse practitioner-based diabetes care management: impact of telehealth or telephone intervention on glycaemic control | Disease Management & Health Outcomes | 15 | 6 | 377-385 |
| Cho, J.; Thorud, J. L.; Marishak-Simon, S.; Frawley, L.; Stevens, A. B. | 2015 | A Model Home-Delivered Meals Program to Support Transitions from Hospital to Home | Journal of Nutrition in Gerontology & Geriatrics | 34 | 2 | 207-17 |
| Cohen, Marya J.; Morton, Suzanne; Scholle, Sarah Hudson; Solberg, Leif I.; Kormos, William A. | 2014 | Self-management support activities in patient-centered medical home practices | Journal of Ambulatory Care Management | 37 | 4 | 349-358 |
| Cook, A.; Grothaus, C. T.; Gutierrez, C. E.; Kehoe, K. A.; Valentin, M. R. | 2010 | Closing the gap "Disparity in native Hawaiian cardiac care" | Hawaii Medical Journal | 69 | 5 Suppl 2 |  |
| Covinsky, K. E.; Palmer, R. M.; Kresevic, D. M.; Kahana, E.; Counsell, S. R.; Fortinsky, R. H.; Landefeld, C. S. | 1998 | Improving functional outcomes in older patients: Lessons from an acute care for elders unit | Joint Commission Journal on Quality Improvement | 24 | 2 | 63-76 |
| Delparte, J. J.; Chau, B. A.; Mills, S.; Burns, A. S. | 2014 | Spinal cord essentials: The development of an individualized, handout-based patient and family education initiative for people with spinal cord injury | Spinal Cord | 52 | 5 | 400-6 |
| Dowson, C. A.; Kuijer, R. G.; Town, I. G.; Mulder, R. T. | 2010 | Impact of panic disorder upon self-management educational goals in chronic obstructive pulmonary disease? | Chronic Respiratory Disease | 7 | 2 | 83-90 |
| Fassl, B. A.; Nkoy, F. L.; Stone, B. L.; Srivastava, R.; Simon, T. D.; Uchida, D. A.; Koopmeiners, K.; Greene, T.; Cook, L. J.; Maloney, C. G. | 2012 | The Joint Commission children's asthma care quality measures and asthma readmissions | Paediatrics | 130 | 3 | 482-91 |
| Goldfield, Norbert; Kelly, William P.; Patel, Kavita | 2012 | Potentially preventable events: An actionable set of measures for linking quality improvement and cost savings | Quality Management in Health Care | 21 | 4 | 213-219 |
| Griffiths, P.; Wilson-Barnett, J.; Richardson, G.; Spilsbury, K.; Miller, F.; Harris, R. | 2000 | The effectiveness of intermediate care in a nursing-led in-patient unit | International Journal of Nursing Studies | 37 | 2 | 153-61 |
| Irewall, A. L.; Ogren, J.; Bergstrom, L.; Laurell, K.; Soderstrom, L.; Mooe, T. | 2015 | Nurse-led, telephone-based, secondary preventive follow-up after stroke or transient ischemic attack improves blood pressure and ldl cholesterol: Results from the first 12 months of the randomized, controlled NAILED stroke risk factor trial | PLoS ONE [Electronic Resource] | 10 | 10 | e0139997 |
| Jia, H.; Chuang, H.; Wu, S. S.; Wang, X.; Chumbler, N. R. | 2009 | Long-term effect of home telehealth services on preventable hospitalization use | Journal of Rehabilitation Research & Development | 46 | 5 | 557-566 |
| Jordan, C. J.; Goldstein, R. Y.; Michels, R. F.; Hutzler, L.; Slover, J. D.; Bosco, J. A., 3rd | 2012 | Comprehensive program reduces hospital readmission rates after total joint arthroplasty | American Journal of Orthopaedics (Chatham, Nj) | 41 | 11 | 147-51 |
| Krantz, M. J.; Havranek, E. P.; Haynes, D. K.; Smith, I.; Bucher-Bartelson, B.; Long, C. S. | 2008 | Inpatient initiation of beta-blockade plus nurse management in vulnerable heart failure patients: A randomized study | Journal of Cardiac Failure | 14 | 4 | 303-9 |
| Liesenfeld, B.; Renner, R.; Neese, M.; Hepp, K. D. | 2000 | Telemedical care reduces hypoglycaemias and improves glycaemic control in children and adolescents with type 1 diabetes | Diabetes Technology & Therapeutics | 2 | 4 | 561-7 |
| Lin, R.; Gallagher, R.; Spinaze, M.; Najoumian, H.; Dennis, C.; Clifton-Bligh, R.; Tofler, G. | 2014 | Effect of a patient-directed discharge letter on patient understanding of their hospitalisation | Internal Medicine Journal | 44 | 9 | 851-7 |
| Liou, H. L.; Chen, H. I.; Hsu, S. C.; Lee, S. C.; Chang, C. J.; Wu, M. J. | 2015 | The effects of a self-care program on patients with heart failure | Journal of the Chinese Medical Association: JCMA | 78 | 11 | 648-56 |
| McCann-Spry, Lisa; Pelton, JoAnne; Grandy, Glenda; Newell, Dawn | 2016 | An Interdisciplinary approach to reducing length of stay in joint replacement patients | Orthopaedic Nursing | 35 | 5 | 279-300 |
| McMahon, Kevin R. | 2014 | 30-day readmissions rate: What's behind the number? | Long-Term Living: For the Continuing Care Professional | 63 | 8 | 28-31 |
| Merwin, Sara L.; Ismail, Haisam; Kohn, Nina; Chaudhry, Saima I.; Rosenberg, David J. | 2013 | Impact of an educational intervention in a post-discharge VTE prophylaxis quality improvement initiative | Journal of Clinical Outcomes Management | 20 | 10 | 447-454 |
| Murphy, D.; Crowley, R.; Spencer, A.; Birch, M. | 2015 | When can I go home? A prospective case control study to improve communication with patients regarding their diagnosis, treatment plan and likely discharge date | New Zealand Medical Journal | 128 | 1412 | 53-8 |
| Pesut, B.; Hooper, B. P.; Robinson, C. A.; Bottorff, J. L.; Sawatzky, R.; Dalhuisen, M. | 2015 | Feasibility of a rural palliative supportive service | Rural & Remote Health | 15 | 2 | 3116 |
| Rivard, A.; Warren, S.; Voaklander, D.; Jones, A. | 2003 | The efficacy of pre-operative home visits for total hip replacement clients | Canadian Journal of Occupational Therapy - Revue Canadienne d Ergotherapie | 70 | 4 | 226-32 |
| Rosenberg, T. | 2012 | Acute hospital use, nursing home placement, and mortality in a frail community-dwelling cohort managed with primary integrated interdisciplinary elder care at home | Journal of the American Geriatrics Society | 60 | 7 | 1340-6 |
| Samus, Q. M.; Johnston, D.; Black, B. S.; Hess, E.; Lyman, C.; Vavilikolanu, A.; Pollutra, J.; Leoutsakos, J. M.; Gitlin, L. N.; Rabins, P. V.; Lyketsos, C. G. | 2014 | A multidimensional home-based care coordination intervention for elders with memory disorders: the maximizing independence at home (MIND) pilot randomized trial | American Journal of Geriatric Psychiatry | 22 | 4 | 398-414 |
| Sarangarm, P.; London, M. S.; Snowden, S. S.; Dilworth, T. J.; Koselke, L. R.; Sanchez, C. O.; D'Angio, R.; Ray, G. | 2013 | Impact of pharmacist discharge medication therapy counselling and disease state education: Pharmacist Assisting at Routine Medical Discharge (project PhARMD) | American Journal of Medical Quality | 28 | 4 | 292-300 |
| Smeenk, F. W.; de Witte, L. P.; Nooyen, I. W.; Crebolder, H. F. | 2000 | Effects of transmural care on coordination and continuity of care | Patient Education & Counseling | 41 | 1 | 73-81 |
| Smith, C. E.; Piamjariyakul, U.; Werkowitch, M.; Yadrich, D. M.; Thompson, N.; Hooper, D.; Nelson, E. L. | 2016 | A clinical trial of translation of evidence-based interventions to mobile tablets and illness specific internet sites | International Journal of Sensor Networks & Data Communications Print | 5 | 1 |  |
| Verloo, H.; Goulet, C.; Morin, D.; von Gunten, A. | 2015 | effect estimation of an innovative nursing intervention to improve delirium among home-dwelling older adults: A randomized controlled pilot trial | Dementia and Geriatric Cognitive Disorders Extra | 5 | 1 | 176-90 |
| Wang, T. C.; Tsai, A. C.; Wang, J. Y.; Lin, Y. T.; Lin, K. L.; Chen, J. J.; Lin, B. Y.; Lin, T. C. | 2015 | Caregiver-mediated intervention can improve physical functional recovery of patients with chronic stroke: a randomized controlled trial | Neurorehabilitation & Neural Repair | 29 | 1 |  |
| Wiggins, S. A. | 2009 | Family exemplars during implementation of a home pain management intervention | Issues in Comprehensive Paediatric Nursing | 32 | 4 | 160-79 |
| Wu, S.; Tyler, A.; Logsdon, T.; Holmes, N. M.; Balkian, A.; Brittan, M.; Hoover, L.; Martin, S.; Paradis, M.; Sparr-Perkins, R.; Stanley, T.; Weber, R.; Saysana, M. | 2016 | A quality improvement collaborative to improve the discharge process for hospitalized children | Paediatrics | 138 | 2 |  |
| Yu-Yahiro, J. A.; Resnick, B.; Orwig, D.; Hicks, G.; Magaziner, J. | 2009 | Design and implementation of a home-based exercise program post-hip fracture: The Baltimore hip studies experience | Pm & R | 1 | 4 | 308-18 |

**Wrong Outcomes (n=19)**

| **Authors** | **Year** | **Title** | **Journal** | **Volume** | **Issue** | **Pages** |
| --- | --- | --- | --- | --- | --- | --- |
| Aguado, O.; Morcillo, C.; Delas, J.; Rennie, M.; Bechich, S.; Schembari, A.; Fernandez, F.; Rosell, F. | 2010 | Long-term implications of a single home-based educational intervention in patients with heart failure | Heart & Lung | 39 | 6 Suppl | S14-22 |
| Clarke, A.; Sohanpal, R.; Wilson, G.; Taylor, S. | 2010 | Patients' perceptions of early supported discharge for chronic obstructive pulmonary disease: A qualitative study | Quality & Safety in Health Care | 19 | 2 | 95-8 |
| Coleman, E. A.; Mahoney, E.; Parry, C. | 2005 | Assessing the quality of preparation for posthospital care from the patient's perspective: The care transitions measure | Medical Care | 43 | 3 | 246-55 |
| Record, J. D.; Niranjan-Azadi, A.; Christmas, C.; Hanyok, L. A.; Rand, C. S.; Hellmann, D. B.; Ziegelstein, R. C. | 2015 | Telephone calls to patients after discharge from the hospital: An important part of transitions of care | Medical Education Online | 20 |  | 26701 |
| Weinberger, M.; Smith, D. M.; Katz, B. P.; Moore, P. S. | 1988 | The cost-effectiveness of intensive post discharge care. A randomized trial | Medical Care | 26 | 11 | 1092-102 |
| Young, J.; O'Connell, B.; McGregor, S. | 2000 | Day surgery patients' convalescence at home: does enhanced discharge education make a difference? | Nursing & Health Sciences | 2 | 1 | 29-39 |
| Brennan, P. F.; Moore, S. M.; Bjornsdottir, G.; Jones, J.; Visovsky, C.; Rogers, M. | 2001 | Heart Care: An Internet-based information and support system for patient home recovery after coronary artery bypass graft (CABG) surgery | Journal of Advanced Nursing | 35 | 5 | 699-708 |
| Epstein-Lubow, G.; Baier, R. R.; Butterfield, K.; Gardner, R.; Babalola, E.; Coleman, E. A.; Gravenstein, S. | 2014 | Caregiver presence and patient completion of a transitional care intervention | American Journal of Managed Care | 20 | 10 | e349-444 |
| Hiltunen, E. F.; Winder, P. A.; Rait, M. A.; Buselli, E. F.; Carroll, D. L.; Rankin, S. H. | 2005 | Implementation of efficacy enhancement nursing interventions with cardiac elders | Rehabilitation Nursing Journal | 30 | 6 | 221-9 |
| Balaban, R. B.; Galbraith, A. A.; Burns, M. E.; Vialle-Valentin, C. E.; Larochelle, M. R.; Ross-Degnan, D. | 2015 | A Patient Navigator Intervention to Reduce Hospital Readmissions among High-Risk Safety-Net Patients: A Randomized Controlled Trial | Journal of General Internal Medicine | 30 | 7 | 907-15 |
| Benor, D. E.; Delbar, V.; Krulik, T. | 1998 | Measuring impact of nursing intervention on cancer patients' ability to control symptoms | Cancer Nursing | 21 | 5 | 320-34 |
| Bronstein, L. R.; Gould, P.; Berkowitz, S. A.; James, G. D.; Marks, K. | 2015 | Impact of a Social Work Care Coordination Intervention on Hospital Readmission: A Randomized Controlled Trial | Social Work | 60 | 3 | 248-55 |
| Burns, M. E.; Galbraith, A. A.; Ross-Degnan, D.; Balaban, R. B. | 2014 | Feasibility and evaluation of a pilot community health worker intervention to reduce hospital readmissions | International Journal for Quality in Health Care | 26 | 4 | 358-65 |
| Coleman, E. A.; Parry, C.; Chalmers, S.; Min, S. J. | 2006 | The care transitions intervention: results of a randomized controlled trial | Archives of Internal Medicine | 166 | 17 | 1822-8 |
| Courtney, M. D.; Edwards, H. E.; Chang, A. M.; Parker, A. W.; Finlayson, K.; Bradbury, C.; Nielsen, Z. | 2012 | Improved functional ability and independence in activities of daily living for older adults at high risk of hospital readmission: a randomized controlled trial | Journal of Evaluation in Clinical Practice | 18 | 1 | 128-34 |
| Gujral, G.; Winckel, K.; Nissen, L. M.; Cottrell, W. N. | 2014 | Impact of community pharmacist intervention discussing patients' beliefs to improve medication adherence | International Journal of Clinical Pharmacy | 36 | 5 | 1048-58 |
| Hanssen, T. A.; Nordrehaug, J. E.; Eide, G. E.; Hanestad, B. R. | 2009 | Does a telephone follow-up intervention for patients discharged with acute myocardial infarction have long-term effects on health-related quality of life? A randomised controlled trial | Journal of Clinical Nursing | 18 | 9 | 1334-45 |
| Naylor, M. D.; Brooten, D.; Campbell, R.; Jacobsen, B. S.; Mezey, M. D.; Pauly, M. V.; Schwartz, J. S. | 1999 | Comprehensive discharge planning and home follow-up of hospitalized elders: a randomized clinical trial | JAMA | 281 | 7 | 613-20 |
| Pezzin, L. E.; Feldman, P. H.; Mongoven, J. M.; McDonald, M. V.; Gerber, L. M.; Peng, T. R. | 2011 | Improving blood pressure control: results of home-based post-acute care interventions | Journal of General Internal Medicine | 26 | 3 | 280-6 |
| Schneider, J. K.; Hornberger, S.; Booker, J.; Davis, A.; Kralicek, R. | 1993 | A medication discharge planning program: measuring the effect on readmissions | Clinical Nursing Research | 2 | 1 | 41-53 |

**Wrong Patient Population (n=30)**

| **Authors** | **Year** | **Title** | **Journal** | **Volume** | **Issue** | **Pages** |
| --- | --- | --- | --- | --- | --- | --- |
| Ballard, F. A. | 2008 | Benefits of psychosocial rehabilitation programming in a treatment mall | Journal of Psychosocial Nursing & Mental Health Services | 46 | 2 | 26-32 |
| Barsamian, A. M.; Gregoire, M.; Sowa, D.; Lafferty, L.; Stone, M. | 2010 | Timely resolution of patient concerns improves post-discharge satisfaction | Journal of the American Dietetic Association | 110 | 9 | 1346-1350 |
| Bauml, J.; Pitschel-Walz, G.; Volz, A.; Engel, R. R.; Kessling, W. | 2007 | Psychoeducation in schizophrenia: 7-year follow-up concerning rehospitalization and days in hospital in the Munich Psychosis Information Project study | Journal of Clinical Psychiatry | 68 | 6 | 854-61 |
| Bonsack, C.; Golay, P.; Gibellini Manetti, S.; Gebel, S.; Ferrari, P.; Besse, C.; Favrod, J.; Morandi, S. | 2016 | Linking primary and secondary care after psychiatric hospitalization: Comparison between transitional case management setting and routine care for common mental disorders | Frontiers in psychiatry Frontiers Research Foundation | 7 |  | 96 |
| Bostelman, S.; Callan, M.; Rolincik, L. C.; Gantt, M.; Herink, M.; King, J.; Massey, M. K.; Morehouse, D.; Sopata, T.; Turner, J. | 1994 | A community project to encourage compliance with mental health treatment aftercare | Public Health Reports | 109 | 2 | 153-7 |
| Bray-Hall, S.; Schmidt, K.; Aagaard, E. | 2010 | Toward safe hospital discharge: A transitions in care curriculum for medical students | Journal of General Internal Medicine | 25 | 8 | 878-81 |
| Cardona, F. A.; Davis, E. R.; Switzer, P. K., 3rd | 1996 | The Kiva project | Journal - South Carolina Medical Association | 92 | 5 | 220-4 |
| Daly, B. J.; Douglas, S. L.; Kelley, C. G.; O'Toole, E.; Montenegro, H. | 2005 | Trial of a disease management program to reduce hospital readmissions of the chronically critically ill | Chest | 128 | 2 | 507-17 |
| Dolansky, M. A.; Zullo, M. D.; Boxer, R. S.; Moore, S. M. | 2011 | Initial efficacy of a cardiac rehabilitation transition program: Cardiac TRUST | Journal of Gerontological Nursing | 37 | 12 | 36-44 |
| Dusing, S. C.; Brown, S. E.; Van Drew, C. M.; Thacker, L. R.; Hendricks-Munoz, K. D. | 2015 | Supporting play exploration and early development intervention from NICU to home: A feasibility study | Paediatric Physical Therapy | 27 | 3 | 267-74 |
| Dyck, D. G.; Weeks, D. L.; Gross, S.; Lederhos Smith, C.; Lott, H. A.; Wallace, A. J.; Wood, S. M. | 2016 | Comparison of two psycho-educational family group interventions for improving psycho-social outcomes in persons with spinal cord injury and their caregivers: A randomized-controlled trial of multi-family group intervention versus an active education control condition | BMC psychology | 4 | 1 | 40 |
| Fens, Manon; Beusmans, George; Limburg, Martien; van Hoef, Liesbeth; van Haastreg, Jolanda; Metsemakers, Job; van Heugten, Caroline | 2015 | A process evaluation of a stroke-specific follow-up care model for stroke patients and caregivers; a longitudinal study | BMC Nursing | 14 | 1 | 20-38 |
| Frich, L. M.; Sorensen, J.; Jacobsen, S.; Fohlmann, B.; Hojsted, J. | 2012 | Outcomes of follow-up visits to chronic non-malignant pain patients | Pain Management Nursing | 13 | 4 | 223-35 |
| Hanrahan, N. P.; Solomon, P.; Hurford, M. O. | 2014 | A pilot randomized control trial: Testing a transitional care model for acute psychiatric conditions | Journal of the American Psychiatric Nurses Association | 20 | 5 | 315-27 |
| Hengartner, M. P.; Passalacqua, S.; Heim, G.; Andreae, A.; Rossler, W.; von Wyl, A. | 2016 | Factors influencing patients' recovery and the efficacy of a psychosocial post-discharge intervention: Post hoc analysis of a randomized controlled trial | Social Psychiatry & Psychiatric Epidemiology | 6 |  | 6 |
| Jenq, G. Y.; Doyle, M. M.; Belton, B. M.; Herrin, J.; Horwitz, L. I. | 2016 | Quasi-experimental evaluation of the effectiveness of a large-scale readmission reduction program | JAMA Internal Medicine | 176 | 5 | 681-90 |
| Jensen, E.; Chapman, P.; Davis, A.; Forchuk, C.; Seymour, B.; Witcher, P.; Armstrong, D. | 2010 | An evaluation of community-based discharge planning in acute mental health care | Canadian Journal of Community Mental Health | 29 |  | 111-124 |
| Kushner, David S.; Peters, Kenneth | 2014 | An inpatient-rehabilitation intervention to increase functional-independence and rate of discharge to home in geriatric patients | American Journal of Physical Medicine & Rehabilitation |  |  | a15-6 |
| Lannin, N. A.; Clemson, L.; McCluskey, A.; Lin, C. W.; Cameron, I. D.; Barras, S. | 2007 | Feasibility and results of a randomised pilot-study of pre-discharge occupational therapy home visits | BMC Health Services Research | 7 |  | 42 |
| Legrain, S.; Tubach, F.; Bonnet-Zamponi, D.; Lemaire, A.; Aquino, J. P.; Paillaud, E.; Taillandier-Heriche, E.; Thomas, C.; Verny, M.; Pasquet, B.; Moutet, A. L.; Lieberherr, D.; Lacaille, S. | 2011 | A new multimodal geriatric discharge-planning intervention to prevent emergency visits and rehospitalizations of older adults: The optimization of medication in AGEd multicenter randomized controlled trial | Journal of the American Geriatrics Society | 59 | 11 | 2017-28 |
| Maples, N. J.; Copeland, L. A.; Zeber, J. E.; Li, X.; Moore, T. A.; Dassori, A.; Velligan, D. I.; Miller, A. L. | 2012 | Can medication management coordinators help improve continuity of care after psychiatric hospitalization? | Psychiatric Services | 63 | 6 | 554-60 |
| Phillips, E. M.; Abrandt, B. L.; Cesta, T.; Gallucci, M. A. | 1999 | Rehabilitation after hip fracture | Topics in Geriatric Rehabilitation | 15 | 1 | 56-65 |
| Rantz, Marilyn J.; Alexander, Greg; Galambos, Colleen; Vogelsmeier, Amy; Popejoy, Lori; Flesner, Marcia; Lueckenotte, Annette; Crecelius, Charles; Zwygart-Stauffacher, Mary; Koopman, Richelle J. | 2014 | Initiative to test a multidisciplinary model with advanced practice nurses to reduce avoidable hospitalizations among nursing facility residents | Journal of Nursing Care Quality | 29 | 1 |  |
| Reidt, Shannon L.; Holtan, Haley S.; Larson, Tom A.; Thompson, Bruce; Kerzner, Lawrence J.; Salvatore, Toni M.; Adam, Terrence J. | 2016 | Interprofessional Collaboration to Improve Discharge from Skilled Nursing Facility to Home: Preliminary Data on Post discharge Hospitalizations and Emergency Department Visits | Journal of the American Geriatrics Society | 64 | 9 | 1895-1899 |
| Reynolds, W.; Lauder, W.; Sharkey, S.; Maciver, S.; Veitch, T.; Cameron, D. | 2004 | The effects of a transitional discharge model for psychiatric patients | Journal of Psychiatric & Mental Health Nursing | 11 | 1 | 82-8 |
| Salas, C. M.; Miyares, M. A. | 2015 | Implementing a pharmacy resident run transition of care service for heart failure patients: Effect on readmission rates | American Journal of Health-System Pharmacy | 72 | 11 Suppl 1 | S43-7 |
| Sniehotta, F. F.; Scholz, U.; Schwarzer, R. | 2006 | Action plans and coping plans for physical exercise: A longitudinal intervention study in cardiac rehabilitation | British Journal of Health Psychology | 11 | Pt 1 | 23-37 |
| Strupeit, S.; Wolf-Ostermann, K.; Bu, A.; Dassen, T. | 2013 | Effectiveness of a nursing consultation intervention for older people with functional mobility impairments: a prospective, longitudinal study | Journal of Nursing Research | 21 | 4 | 279-88 |
| Tiberg, I.; Katarina, S. C.; Carlsson, A.; Hallstrom, I. | 2012 | Children diagnosed with type 1 diabetes: a randomized controlled trial comparing hospital versus home-based care | Acta Paediatrica | 101 | 10 | 1069-73 |
| Youssef, F. A. | 1987 | Discharge planning for psychiatric patients: the effects of a family-patient teaching programme | Journal of Advanced Nursing | 12 | 5 | 611-6 |

**Wrong Setting (n=6)**

| **Authors** | **Year** | **Title** | **Journal** | **Volume** | **Issue** | **Pages** |
| --- | --- | --- | --- | --- | --- | --- |
| Brumley, R. D.; Enguidanos, S.; Cherin, D. A. | 2003 | Effectiveness of a home-based palliative care program for end-of-life | Journal of Palliative Medicine | 6 | 5 | 715-24 |
| Butcher, Lola | 2016 | Oncologists seek to understand, address hospital readmissions | Oncology Times | 38 | 6 |  |
| Cacciola, J. S.; Camilleri, A. C.; Carise, D.; Rikoon, S. H.; McKay, J. R.; McLellan, A. T.; Wilson, C.; Schwarzlose, J. T. | 2008 | Extending residential care through telephone counselling: Initial results from the Betty Ford Center Focused Continuing Care protocol | Addictive Behaviors | 33 | 9 | 1208-16 |
| Leff, B.; Burton, L.; Mader, S. L.; Naughton, B.; Burl, J.; Greenough, W. B.; Guido, S.; Steinwachs, D. | 2009 | Comparison of functional outcomes associated with hospital at home care and traditional acute hospital care | Journal of the American Geriatrics Society | 57 | 2 | 273-278 |
| Pacini, M.; Smith, R. D.; Wilson, E. C.; Holland, R. | 2007 | Home-based medication review in older people: is it cost effective? | Pharmacoeconomics | 25 | 2 | 171-80 |
| Tappen, R. M.; Whitehead, D.; Folden, S. L.; Hall, R. | 2003 | Effect of a video intervention on functional recovery following hip replacement and hip fracture repair | Rehabilitation Nursing Journal | 28 | 5 | 148-53 |

**Wrong Study Design (n=71)**

| **Authors** | **Year** | **Title** | **Journal** | **Volume** | **Issue** | **Pages** |
| --- | --- | --- | --- | --- | --- | --- |
| Acher, A. W.; LeCaire, T. J.; Hundt, A. S.; Greenberg, C. C.; Carayon, P.; Kind, A. J.; Weber, S. M. | 2015 | Using human factors and systems engineering to evaluate readmission after complex surgery | Journal of the American College of Surgeons | 221 | 4 | 810-20 |
| Agelopoulos, N.; Tate, T. | 2009 | The Marie Curie delivering choice programme | European Journal of Palliative Care | 16 | 6 | 290-294 |
| Alvarez, R.; Ginsburg, J.; Grabowski, J.; Post, S.; Rosenberg, W. | 2016 | The social work role in reducing 30-day readmissions: The effectiveness of the bridge model of transitional care | Journal of Gerontological Social Work | 59 | 3 | 222-7 |
| Andrews, Carol A.; Harrington, Suzy | 2013 | Patient-centered medical home in the U.S. air force | AAACN Viewpoint | 35 | 4 |  |
| Annis, Ann M.; Harris, Marcelline; Robinson, Claire H.; Krein, Sarah L. | 2016 | Do patient-centered medical home access and care coordination measures reflect the contribution of all team members? A systematic review | Journal of Nursing Care Quality | 31 | 4 | 357-366 |
| Aston, G. | 2010 | Clinical management series. Creating a cardiac care continuum | H&HN: Hospitals & Health Networks | 84 | 9 | 32-36 |
| Aubry, F; Couturier, Yves; Dumont, Serge | 2014 | The progressive disinterest of family medicine residents in home care for the elderly | Canadian Journal on Aging | 33 | 2 | 176-184 |
| Bailey, A. L.; Moe, G.; Moe, J.; Oland, R. | 2009 | Implementation and evaluation of a community-based medication reconciliation (CMR) system at the hospital-community interface of care | Healthcare Quarterly | 13 Spec No |  | 91-7 |
| Boter, H.; Rinkel, G. J.; de Haan, R. J.; Hestia Study Group | 2004 | Outreach nurse support after stroke: a descriptive study on patients' and carers' needs, and applied nursing interventions | Clinical Rehabilitation | 18 | 2 | 156-63 |
| Brookes, K.; Scott, M. G.; McConnell, J. B. | 2000 | The benefits of a hospital-based community services liaison pharmacist | Pharmacy World & Science | 22 | 2 | 33-8 |
| Buurman, B. M.; Parlevliet, J. L.; van Deelen, B. A.; de Haan, R. J.; de Rooij, S. E. | 2010 | A randomised clinical trial on a comprehensive geriatric assessment and intensive home follow-up after hospital discharge: The transitional care bridge | BMC Health Services Research | 10 |  | 296 |
| Day, K.; Millner, S.; Johnson, H. | 2016 | How nurses use telehealth to support health transitions of older adults | Studies in Health Technology & Informatics | 231 |  | 23-30 |
| Delaney, M. C.; Trachtenberg, J. | 1980 | Discharge planning: A quality assurance program in a cancer research hospital | Cancer Nursing | 3 | 2 | 138-44 |
| Dukeshire, S.; Gilmour, D.; MacDonald, N.; MacKenzie, K. | 2012 | Development and evaluation of a web site to improve recovery from hysterectomy | CIN: Computers, Informatics, Nursing | 30 | 3 | 164-75; quiz 176-7 |
| Eggenberger, T.; Garrison, H.; Hilton, N.; Giovengo, K. | 2013 | Discharge phone calls: Using person-centred communication to improve outcomes | Journal of Nursing Management | 21 | 5 | 733-9 |
| Flowers, L. | 2006 | Are your elderly patients safe to go home? | OR Manager | 22 | 1 | 21-25 |
| Heeke, S.; Wood, F.; Schuck, J. | 2014 | Improving care transitions from hospital to home: Standardized orders for home health nursing with remote telemonitoring | Journal of Nursing Care Quality | 29 | 2 | E21-8 |
| Herzig, S. J.; Schnipper, J. L.; Doctoroff, L.; Kim, C. S.; Flanders, S. A.; Robinson, E. J.; Ruhnke, G. W.; Thomas, L.; Kripalani, S.; Lindenauer, P. K.; Williams, M. V.; Metlay, J. P.; Auerbach, A. D. | 2016 | Physician perspectives on factors contributing to readmissions and potential prevention strategies: A multicenter survey | Journal of General Internal Medicine | 31 | 11 | 1287-1293 |
| Hobbs, Joanne Kathleen | 2016 | CNE SERIES. Reducing hospital readmission rates in patients with heart failure | Medsurg Nursing | 25 | 3 | 145-152 |
| Horwitz, L. I.; Moriarty, J. P.; Chen, C.; Fogerty, R. L.; Brewster, U. C.; Kanade, S.; Ziaeian, B.; Jenq, G. Y.; Krumholz, H. M. | 2013 | Quality of discharge practices and patient understanding at an academic medical center | JAMA Internal Medicine | 173 | 18 | 1715-22 |
| Jones, M.; Gassaway, J. | 2016 | Peer-supported self-management to facilitate community re-entry after discharge from spinal cord injury rehabilitation: Engaging peers as change agents and research partners | Annals of Physical & Rehabilitation Medicine | 59S |  | e128-e129 |
| Kasteleyn, M. J.; Gorter, K. J.; Stellato, R. K.; Rijken, M.; Nijpels, G.; Rutten, G. E. | 2014 | Tailored support for type 2 diabetes patients with an acute coronary event after discharge from hospital - design and development of a randomised controlled trial | Diabetology & metabolic syndrome | 6 | 1 | 5 |
| Kelly, Michelle D. | 2011 | Self-management of chronic disease and hospital readmission: A care transition strategy | Journal of Nursing & Healthcare of Chronic Illnesses | 3 | 1 |  |
| Larsen, A. S. | 1988 | Helping patients avoid readmission to hospital: a health behaviour study | Recent Advances in Nursing | 22 |  | 62-88 |
| Lee, Phillip H.; Calhoun, McKenzie L.; Stewart, David W.; Cross, L. Brian | 2014 | Transition of care in patients with heart failure | Home Health Care Management & Practice | 26 | 1 | 39-44 |
| Li, J.; Young, R.; Williams, M. V. | 2014 | Optimizing transitions of care to reduce rehospitalizations | Cleveland Clinic Journal of Medicine | 81 | 5 | 312-20 |
| Marrett, M.; Christerson, M.; Blackburn, A.; Layfield, T.; Masters, J. | 2011 | Data bits. Discharge education to prevent readmissions in older patients | Kentucky Nurse | 59 | 3 | 7 |
| McBryde, M.; Vandiver, J. W.; Onysko, M. | 2016 | Transitions of care in medical education: A compilation of effective teaching methods | Family Medicine | 48 | 4 | 265-72 |
| McCauley, K. M.; Bixby, M. B.; Naylor, M. D. | 2006 | Advanced practice nurse strategies to improve outcomes and reduce cost in elders with heart failure | Disease Management | 9 | 5 | 302-10 |
| Meadows, C.; Camus, S.; Fraser, J. | 2015 | transforming community access services through client- and family-centred homecare transitions | Nursing leadership (Toronto, Ont.) | 28 | 1 | 73-83 |
| Paul, S. | 2008 | Hospital discharge education for patients with heart failure: What really works and what is the evidence? | Critical Care Nurse | 28 | 2 | 66-82 |
| Pedepeyreau, C.; Reznikoff, V. | 2010 | Hospitalization at home | Soins; La Revue de Reference Infirmiere | Gerontologie. | 86 | 43-4 |
| Pickler, R.; Wade-Murphy, S.; Gold, J.; Tubbs-Cooley, H.; White, C. M.; Statile, A.; Hoying, C.; Sauers-Ford, H.; Shah, S. S.; Simmons, J. | 2016 | A nurse transitional home visit following paediatric hospitalizations | Journal of Nursing Administration | 46 | 12 | 642-647 |
| Remy, I. | 2007 | Bretonneau mobile team, a bridge between the hospital and the community | Soins; La Revue de Reference Infirmiere | Gerontologie. | 64 | 35-8 |
| Robinson, L.; Stansbury, L. | 2010 | CMS-funded care transitions health care quality improvement project cuts hospital readmission rate in coached population | Remington Report | 18 | 4 |  |
| Rydeman, I.; TÃ¶rnkvist, L. | 2010 | Getting prepared for life at home in the discharge process - from the perspective of the older persons and their relatives | International Journal of Older People Nursing | 5 | 4 | 254-264 |
| Salim Al-Damluji, M.; Dzara, K.; Hodshon, B.; Punnanithinont, N.; Krumholz, H. M.; Chaudhry, S. I.; Horwitz, L. I. | 2015 | Association of discharge summary quality with readmission risk for patients hospitalized with heart failure exacerbation | Circulation. Cardiovascular Quality & Outcomes | 8 | 1 | 109-11 |
| Savage, J. | 1992 | Advice to take home | Nursing Times | 88 | 38 |  |
| Stewart, K. | 2010 | Bliss relaunches Going Home Pack | Journal of Neonatal Nursing | 16 | 5 | 210-210 |
| Szunyog, C. L. | 1987 | Getting I.V. patients ready to go home | RN | 50 | 10 | 136-8 |
| Vall-Spinosa, A. | 2013 | Carrot or stick? Improving the discharge process | Journal of the American Medical Directors Association | 14 | 2 | 143 |
| VanLeeuwen, Shawna; Leenerts, Mary H.; Moran, Mona | 2012 | Assess patients for depression for a safer discharge | Nursing | 42 | 10 | 50-56 |
| Weaver, C. A. | 2009 | Home sweet home | South Dakota Medicine: The Journal of the South Dakota State Medical Association | 62 | 7 | 264 |
| Werth, S. L.; Schutte, D. L.; Stommel, M. | 2014 | Bridging the gap: Perceived educational needs in the inpatient to home care setting for the person with a new ostomy | Journal of Wound, Ostomy, & Continence Nursing | 41 | 6 | 566-72 |
| Anderson, C.; Deepak, B. V.; Amoateng-Adjepong, Y.; Zarich, S. | 2005 | Benefits of comprehensive inpatient education and discharge planning combined with outpatient support in elderly patients with congestive heart failure | Congestive Heart Failure | 11 | 6 | 315-21 |
| Coleman, E. A.; Smith, J. D.; Frank, J. C.; Min, S. J.; Parry, C.; Kramer, A. M. | 2004 | Preparing patients and caregivers to participate in care delivered across settings: The Care Transitions Intervention | Journal of the American Geriatrics Society | 52 | 11 | 1817-25 |
| Coleman, E. A.; Roman, S. P.; Hall, K. A.; Min, S. J. | 2015 | Enhancing the care transitions intervention protocol to better address the needs of family caregivers | Journal for Healthcare Quality | 37 | 1 |  |
| Ekman, I.; Wolf, A.; Olsson, L. E.; Taft, C.; Dudas, K.; Schaufelberger, M.; Swedberg, K. | 2012 | Effects of person-centred care in patients with chronic heart failure: The PCC-HF study | European Heart Journal | 33 | 9 | 1112-9 |
| Fera, T.; Anderson, C.; Kanel, K. T.; Ramusivich, D. L. | 2014 | Role of a care transition pharmacist in a primary care resource center | American Journal of Health-System Pharmacy | 71 | 18 | 1585-90 |
| Fleming, M. O.; Haney, T. T. | 2013 | Improving patient outcomes with better care transitions: The role for home health | Cleveland Clinic Journal of Medicine | 80 Electronic Suppl 1 |  | eS2-6 |
| Goldstein, N. L.; Snyder, M.; Edin, C.; Lindgren, B.; Finkelstein, S. M. | 1996 | Comparison of two teaching strategies: Adherence to a home monitoring program | Clinical Nursing Research | 5 | 2 | 150-66 |
| Graham, J.; Tomcavage, J.; Salek, D.; Sciandra, J.; Davis, D. E.; Stewart, W. F. | 2012 | Post discharge monitoring using interactive voice response system reduces 30-day readmission rates in a case-managed Medicare population | Medical Care | 50 | 1 | 50-57 |
| Hyrkas, K.; Wiggins, M. | 2014 | A comparison of usual care, a patient-centred education intervention and motivational interviewing to improve medication adherence and readmissions of adults in an acute-care setting | Journal of Nursing Management | 22 | 3 | 350-61 |
| Knier, S.; Stichler, J. F.; Ferber, L.; Catterall, K. | 2015 | Patients' perceptions of the quality of discharge teaching and readiness for discharge | Rehabilitation Nursing Journal | 40 | 1 |  |
| Li, J.; Young, R.; Williams, M. V. | 2014 | Optimizing transitions of care to reduce rehospitalizations | Cleveland Clinic Journal of Medicine | 81 | 5 | 312-20 |
| Missel, Malene; SchÃ¸nau, Mai Nanna; Pedersen, Jesper Holst; Pedersen, Preben Ulrich | 2015 | Transition from hospital to daily life: A pilot study | Rehabilitation Nursing | 40 | 1 | 20-29 |
| Rebello, K. E.; Gosian, J.; Salow, M.; Sweeney, P.; Rudolph, J. L.; Driver, J. A. | 2016 | The Rural PILL Program: A post discharge tele-pharmacy Intervention for Rural Veterans | Journal of Rural Health | 8 |  | 8 |
| Shu, C. C.; Hsu, N. C.; Lin, Y. F.; Wang, J. Y.; Lin, J. W.; Ko, W. J. | 2011 | Integrated post discharge transitional care in a hospitalist system to improve discharge outcome: An experimental study | BMC Medicine | 9 |  | 96 |
| Styrborn, K. | 1995 | Early discharge planning for elderly patients in acute hospitals - an intervention study | Scandinavian Journal of Social Medicine | 23 | 4 | 273-285 |
| Ulin, K.; Olsson, L. E.; Wolf, A.; Ekman, I. | 2016 | Person-centred care - An approach that improves the discharge process | European Journal of Cardiovascular Nursing | 15 | 3 | e19-26 |
| DiGioia, A., 3rd; Greenhouse, P. K.; Levison, T. J. | 2007 | Patient and family-centered collaborative care: An orthopaedic model | Clinical Orthopaedics & Related Research | 463 |  |  |
| Betts, Veronica | 2014 | Implementing a discharge process change using the teach-back method for COPD Patients |  | PhD |  |  |
| Cunningham, R. S. | 2003 | Advanced practice nursing interventions and outcomes: An exploration of transitional care services post prostatectomy |  | PhD |  |  |
| Daly, J. M. | 1992 | Systematic evaluation of a nursing intervention: Discharge planning |  | PhD |  |  |
| Duffy, Lisa Vittoria | 2013 | Testing the efficacy of the creating opportunities for parent empowerment (COPE) intervention during hospital to home transition: Empowering parents of children with epilepsy and other neurological conditions |  | PhD |  |  |
| Haddock, K. S. | 1988 | Characteristics of effective discharge planning programs: The nursing administration perspective |  | PhD |  |  |
| Hill, Brent D. | 2014 | Automated pictograph enhancement of discharge instructions: Impact on recall and satisfaction | ProQuest Dissertations Publishing | Ph.D. |  |  |
| King, T. L. | 2008 | The impact of a nurse-driven evidence-based discharge planning protocol on organizational efficiency and patient satisfaction in patients with cardiac implants |  | PhD |  |  |
| Koshy, Rachel | 2014 | Impact of the discharge education plan on 30-day heart failure hospital readmission rates of elderly patients |  | PhD |  |  |
| Phillips, C. Y. | 1990 | The effect of post discharge follow-up care on selected outcomes of hospitalized surgical patients |  | PhD |  |  |
| Watkins, G. R. | 2001 | Effect of pain education of postoperative pain management |  | PhD |  |  |
